# Supplementary figures and images for: PBX1 and PBX3 transcription factors regulate SHH expression in the Frontonasal Ectodermal Zone through complementary mechanisms
Source: PLoS Genet. 2025 May 21;21(5):e1011315. doi: 10.1371/journal.pgen.1011315 (PMC12140432; doi:10.1371/journal.pgen.1011315)

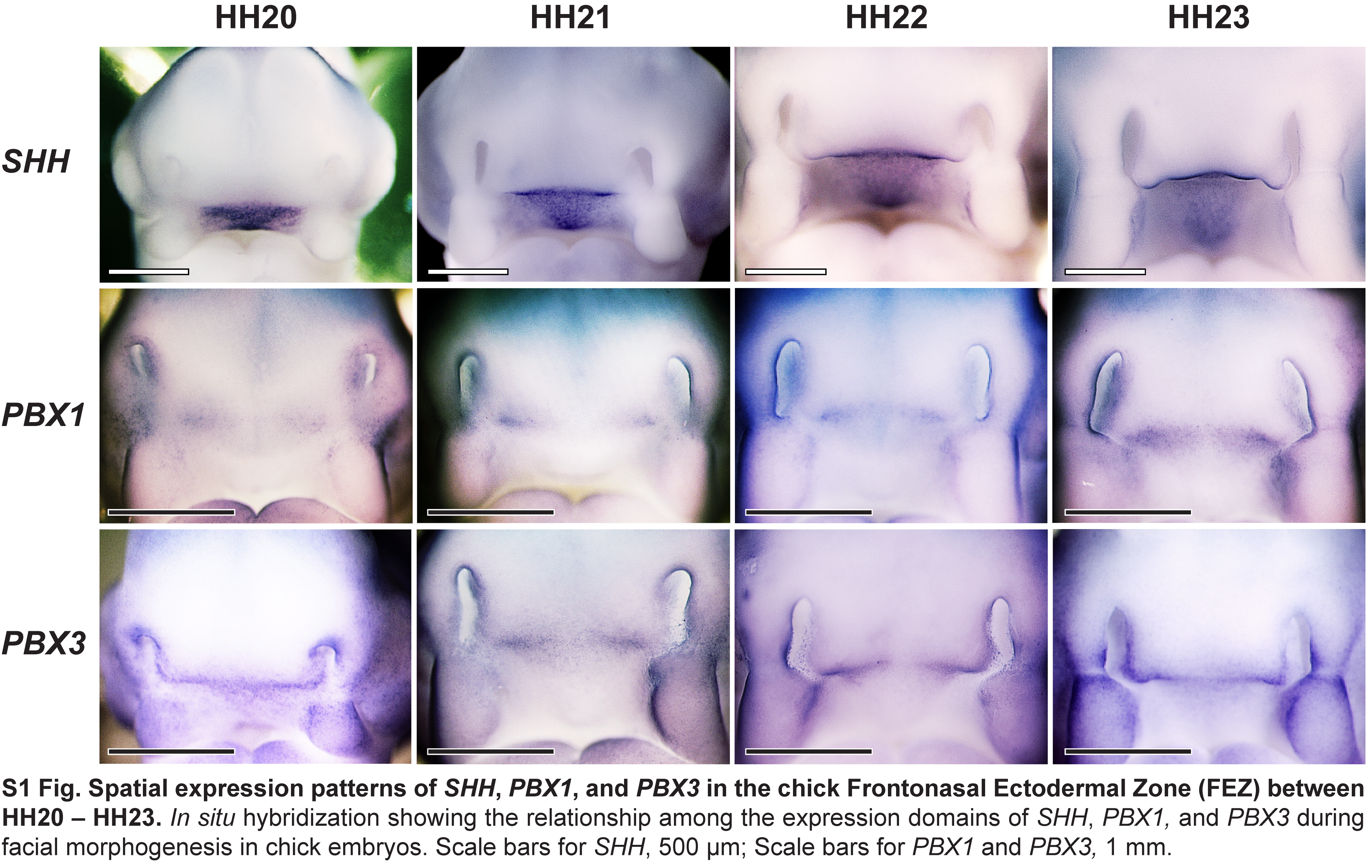

Supplement: S1 Fig — In situ hybridization showing the relationship among the expression domains of SHH, PBX1, and PBX3 during facial morphogenesis in chick embryos. Scale bars for SHH, 500 µm; Scale bars for PBX1 and PBX3, 1 mm. (TIF) [file pgen.1011315.s001.tif]

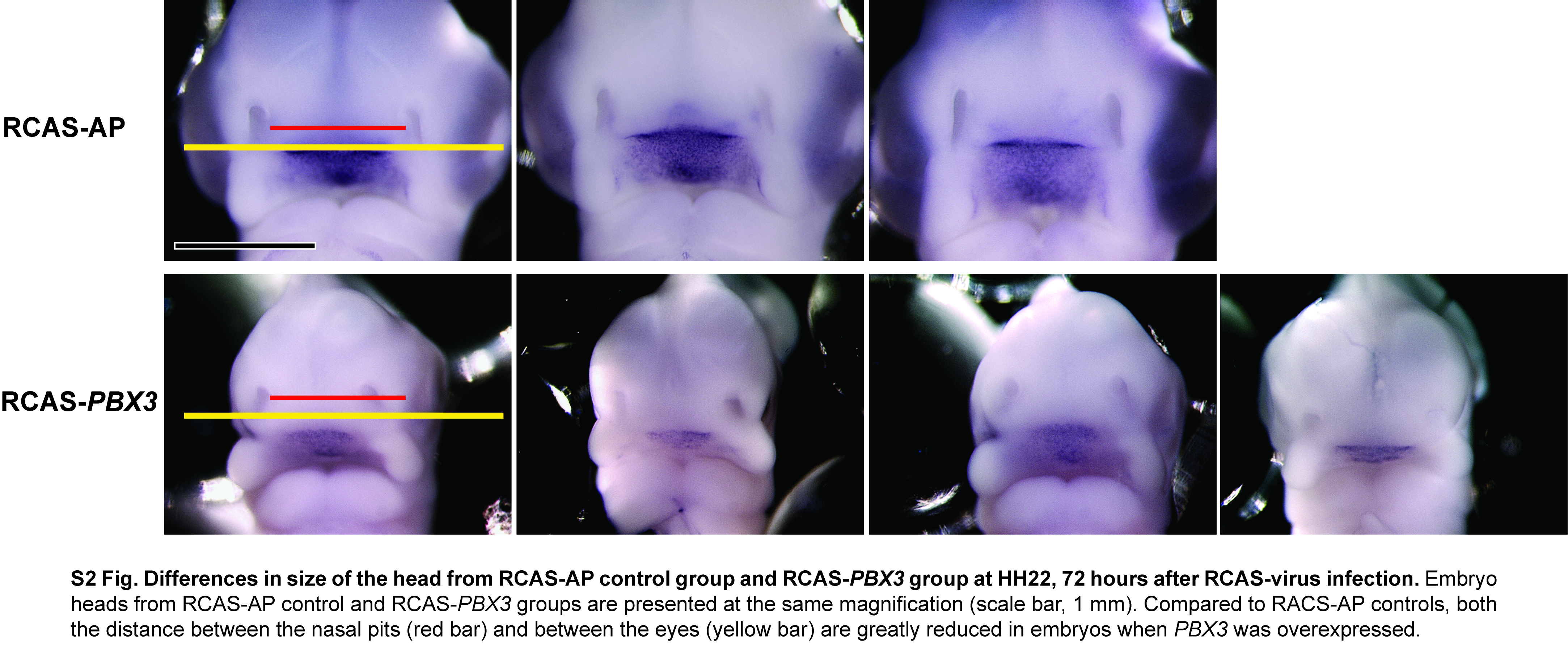

Supplement: S2 Fig — Embryo heads from RCAS-AP control and RCAS-PBX3 groups are presented at the same magnification (scale bar, 1 mm). Compared to RACS-AP controls, both the distance between the nasal pits (red bar) and between the eyes (yellow bar) are greatly reduced in embryos when PBX3 was overexpressed. (TIF) [file pgen.1011315.s002.tif]

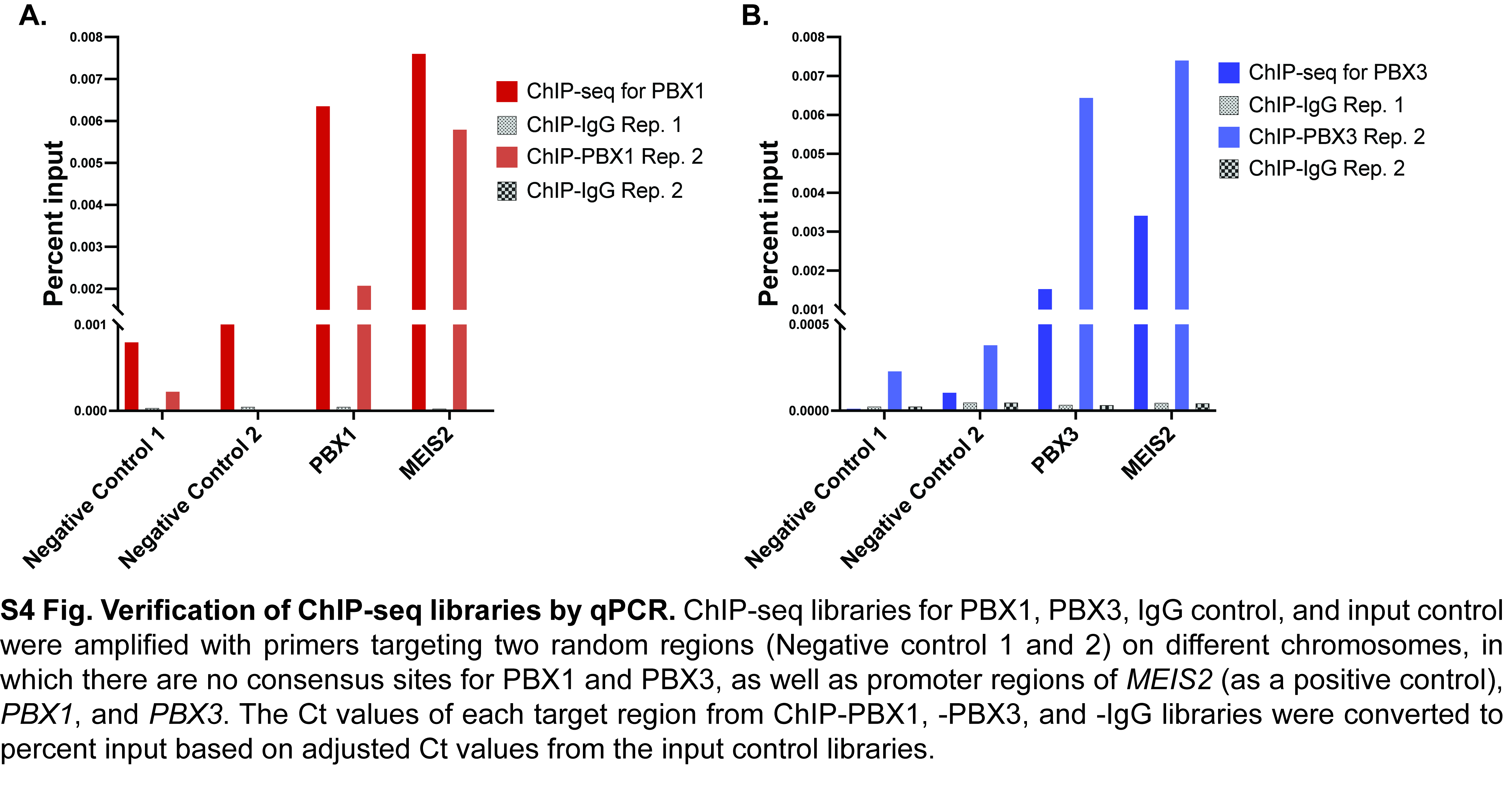

Supplement: S4 Fig — ChIP-seq libraries for PBX1, PBX3, IgG control, and input control were amplified with primers targeting two random regions (Negative control 1 and 2) on different chromosomes, in which there are no consensus sites for PBX1 and PBX3, as well as promoter regions of MEIS2 (as a positive control), PBX1, and PBX3. The Ct values of each target region from ChIP-PBX1, -PBX3, and -IgG libraries were converted to percent input based on adjusted Ct values from the input control libraries. (TIF) [file pgen.1011315.s004.tif]

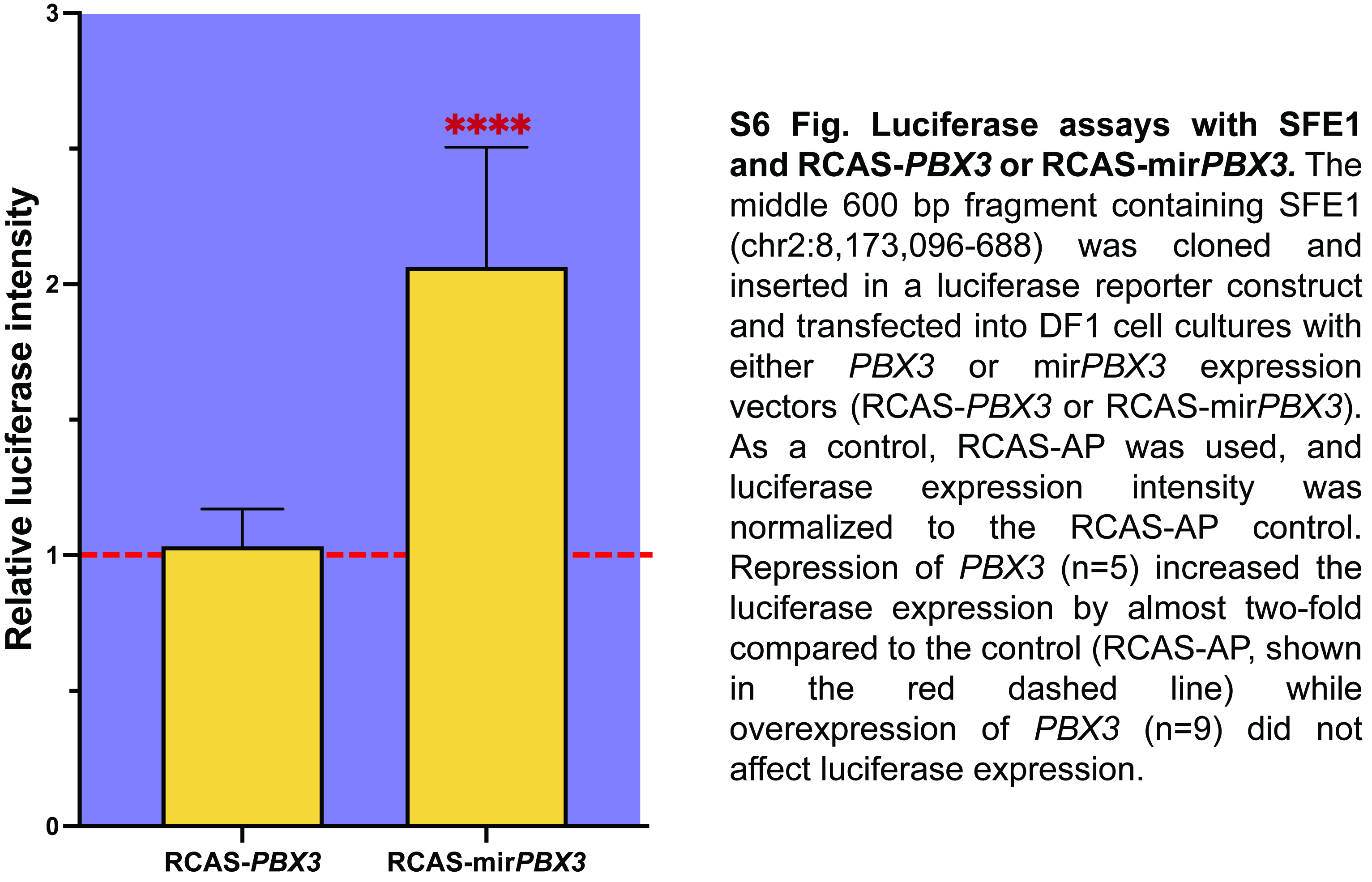

Supplement: S6 Fig — The middle 600 bp fragment containing SFE1 (chr2:8,173,096-688) was cloned and inserted in a luciferase reporter construct and transfected into DF1 cell cultures with either PBX3 or mirPBX3 expression vectors (RCAS-PBX3 or RCAS-mirPBX3). As a control, RCAS-AP was used, and luciferase expression intensity was normalized to the RCAS-AP control. Repression of PBX3 (n = 5) increased the luciferase expression by almost two-fold compared to the control (RCAS-AP, shown in the red dashed line) while overexpression of PBX3 (n = 9) did not affect luciferase expression. (TIF) [file pgen.1011315.s006.tif]
