# Supplementary material for: PBX1 and PBX3 transcription factors regulate SHH expression in the Frontonasal Ectodermal Zone through complementary mechanisms
Source: PLoS Genet. 2025 May 21;21(5):e1011315. doi: 10.1371/journal.pgen.1011315 (PMC12140432; doi:10.1371/journal.pgen.1011315)
Supplement: S1 Table — (PDF) [file pgen.1011315.s007.pdf]

S1 Table. Sequencing results of ATAC-seq and ChIP-seq data

**Sequencing type**	**Sample**	**Mapping rate, %**	**Sequencing depth, reads**	**Peaks of reads**	**Simple overlapping peaks**	**Overlapping peaks (IDR<0.05)**
ATAC-seq	Replicate 1	95.50	585,502,859	246,299	188,247	65,556
	Replicate 2	96.19	707,110,287	286,526		
ChIP-seq for PBX1	Replicate 1	88.56	30,851,196	44,892	32,849	13,430
	Input of Replicate 1	94.20	29,674,869			
	Replicate 2	91.13	36,440,529	37,647		
	Input of Replicate 2	93.25	40,986,372			
ChIP-seq for PBX3	Replicate 1	93.42	80,040,505	138,442	86,512	36,122
	Input of Replicate 1	97.75	90,585,798			
	Replicate 2	95.29	25,146,384	99,061		
	Input of Replicate 2	98.18	46,876,410			

Samples were generated from the chick FEZ at HH22 (n=2).

Sequencing was conducted by HiSeq 4000 (Illumina).

Paired-end reads (2×150 nt) were sequenced for ATAC-seq data, and single-end reads (50 nt) were sequenced for ChIP-seq data.
