# Supplementary material for: PBX1 and PBX3 transcription factors regulate SHH expression in the Frontonasal Ectodermal Zone through complementary mechanisms
Source: PLoS Genet. 2025 May 21;21(5):e1011315. doi: 10.1371/journal.pgen.1011315 (PMC12140432; doi:10.1371/journal.pgen.1011315)
Supplement: S2 Table — (PDF) [file pgen.1011315.s008.pdf]

S2 Table. Full list of known motif discovery from ATAC-seq data.

Homer Known Motif Enrichment Results (/wynton/group/marcucio/2022CHM/Data2022Mar/Motif/HomerATACgiven)

Homer *de novo* Motif Results
Gene Ontology Enrichment Results
Known Motif Enrichment Results (txt file)
Total Target Sequences = 65546, Total Background Sequences = 65439

Rank	Motif	Name	P-value	log P-pvalue	q-value (Benjamini)	# Target Sequences with Motif	% of Targets Sequenc with Motif
1	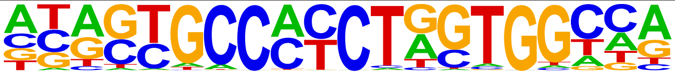	CTCF(Zf)/CD4+-CTCF-ChIP-Seq(Barski_et_al.)/Homer	1e-1698	-3.910e+03	0.0000	6181.0	9.43%
2	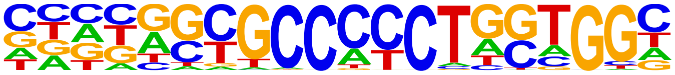	BORIS(Zf)/K562-CTCFL-ChIP-Seq(GSE32465)/Homer	1e-1162	-2.676e+03	0.0000	7937.0	12.11%
3	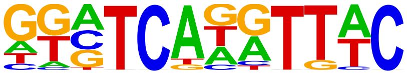	Six1(Homeobox)/Myoblast-Six1-ChIP-Chip(GSE20150)/Homer	1e-1020	-2.349e+03	0.0000	6792.0	10.36%
4	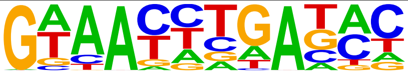	Six2(Homeobox)/NephronProgenitor-Six2-ChIP-Seq(GSE39837)/Homer	1e-926	-2.132e+03	0.0000	18107.0	27.62%
5	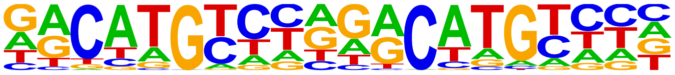	p53(p53)/Saos-p53-ChIP-Seq(GSE15780)/Homer	1e-681	-1.568e+03	0.0000	3107.0	4.74%
6	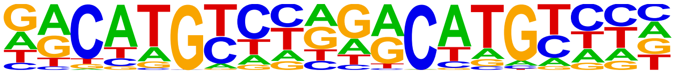	p53(p53)/Saos-p53-ChIP-Seq/Homer	1e-681	-1.568e+03	0.0000	3107.0	4.74%
7	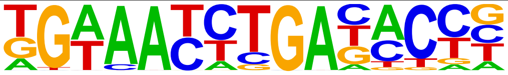	Six4(Homeobox)/MCF7-SIX4-ChIP-Seq(Encode)/Homer	1e-649	-1.497e+03	0.0000	2040.0	3.11%
8	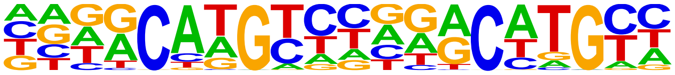	p63(p53)/Keratinocyte-p63-ChIP-Seq(GSE17611)/Homer	1e-647	-1.491e+03	0.0000	8171.0	12.46%
9	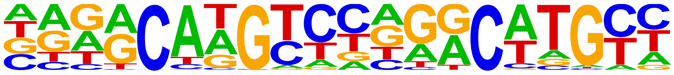	p73(p53)/Trachea-p73-ChIP-Seq(PRJNA310161)/Homer	1e-572	-1.319e+03	0.0000	2037.0	3.11%
10	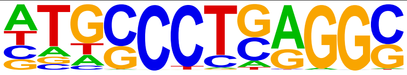	AP-2alpha(AP2)/Hela-AP2alpha-ChIP-Seq(GSE31477)/Homer	1e-520	-1.198e+03	0.0000	16736.0	25.53%
11	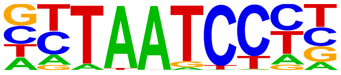	Otx2(Homeobox)/EpiLC-Otx2-ChIP-Seq(GSE56098)/Homer	1e-450	-1.037e+03	0.0000	13393.0	20.43%
12	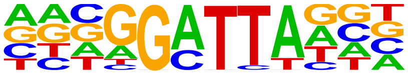	bcd(Homeobox)/Embryo-Bcd-ChIP-Seq(GSE86966)/Homer	1e-426	-9.817e+02	0.0000	18093.0	27.60%
13	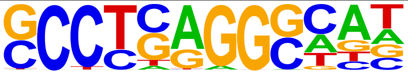	AP-2gamma(AP2)/MCF7-TFAP2C-ChIP-Seq(GSE21234)/Homer	1e-425	-9.805e+02	0.0000	20164.0	30.76%
14	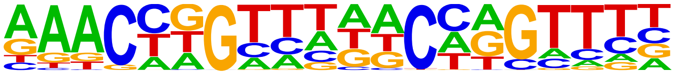	GRHL2(CP2)/HBE-GRHL2-ChIP-Seq(GSE46194)/Homer	1e-400	-9.223e+02	0.0000	7540.0	11.50%
15	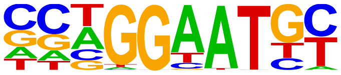	TEAD4(TEA)/Tropoblast-Tead4-ChIP-Seq(GSE37350)/Homer	1e-360	-8.300e+02	0.0000	13905.0	21.21%
16	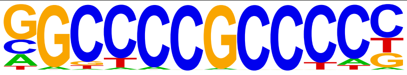	Sp1(Zf)/Promoter/Homer	1e-359	-8.281e+02	0.0000	6284.0	9.59%
17	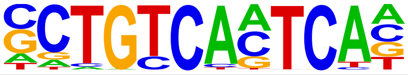	Pbx3(Homeobox)/GM12878-PBX3-ChIP-Seq(GSE32465)/Homer	1e-343	-7.901e+02	0.0000	5100.0	7.78%
18	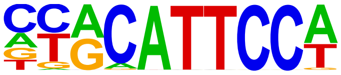	TEAD1(TEAD)/HepG2-TEAD1-ChIP-Seq(Encode)/Homer	1e-331	-7.631e+02	0.0000	14993.0	22.87%
19	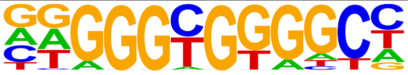	KLF1(Zf)/HUDEP2-KLF1-CutnRun(GSE136251)/Homer	1e-325	-7.486e+02	0.0000	11796.0	17.99%
20	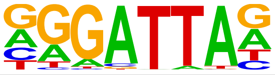	GSC(Homeobox)/FrogEmbryos-GSC-ChIP-Seq(DRA000576)/Homer	1e-319	-7.357e+02	0.0000	17430.0	26.59%

21	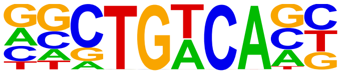	Meis1(Homeobox)/MastCells-Meis1-ChIP-Seq(GSE48085)/Homer	1e-309	-7.116e+02	0.0000	28924.0	44.12%
22	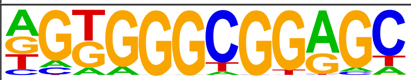	Sp5(Zf)/mES-Sp5.Flag-ChIP-Seq(GSE72989)/Homer	1e-307	-7.071e+02	0.0000	14866.0	22.68%
23	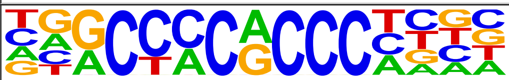	KLF3(Zf)/MEF-Klf3-ChIP-Seq(GSE44748)/Homer	1e-296	-6.822e+02	0.0000	6815.0	10.40%
24	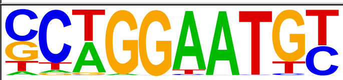	TEAD2(TEA)/Py2T-Tead2-ChIP-Seq(GSE55709)/Homer	1e-293	-6.769e+02	0.0000	8930.0	13.62%
25	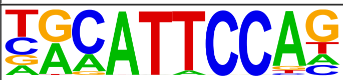	TEAD3(TEA)/HepG2-TEAD3-ChIP-Seq(Encode)/Homer	1e-280	-6.465e+02	0.0000	17088.0	26.07%
26	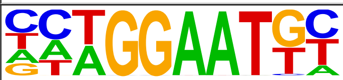	TEAD(TEA)/Fibroblast-PU.1-ChIP-Seq(Unpublished)/Homer	1e-266	-6.147e+02	0.0000	10858.0	16.56%
27	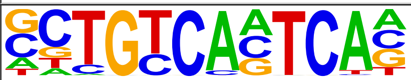	Pknox1(Homeobox)/ES-Prep1-ChIP-Seq(GSE63282)/Homer	1e-260	-6.002e+02	0.0000	4840.0	7.38%
28	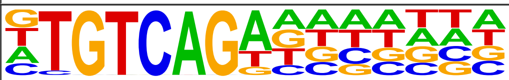	AtGRF6(GRF)/col-AtGRF6-DAP-Seq(GSE60143)/Homer	1e-256	-5.902e+02	0.0000	21327.0	32.53%
29	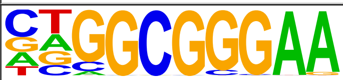	E2F1(E2F)/Hela-E2F1-ChIP-Seq(GSE22478)/Homer	1e-251	-5.788e+02	0.0000	4978.0	7.59%
30	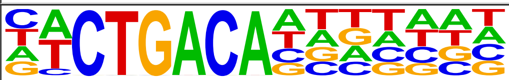	GRF9(GRF)/colamp-GRF9-DAP-Seq(GSE60143)/Homer	1e-248	-5.731e+02	0.0000	18346.0	27.99%
31	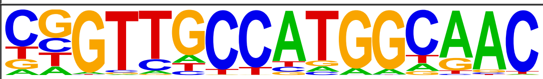	RFX(HTH)/K562-RFX3-ChIP-Seq(SRA012198)/Homer	1e-244	-5.620e+02	0.0000	2160.0	3.29%
32	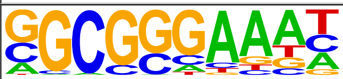	E2F4(E2F)/K562-E2F4-ChIP-Seq(GSE31477)/Homer	1e-228	-5.269e+02	0.0000	7658.0	11.68%
33	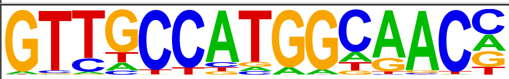	Rfx2(HTH)/LoVo-RFX2-ChIP-Seq(GSE49402)/Homer	1e-214	-4.939e+02	0.0000	2291.0	3.49%
34	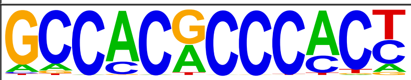	Klf9(Zf)/GBM-Klf9-ChIP-Seq(GSE62211)/Homer	1e-202	-4.661e+02	0.0000	4982.0	7.60%
35	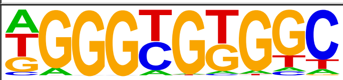	KLF5(Zf)/LoVo-KLF5-ChIP-Seq(GSE49402)/Homer	1e-200	-4.615e+02	0.0000	16131.0	24.61%
36	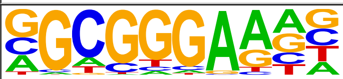	E2F6(E2F)/Hela-E2F6-ChIP-Seq(GSE31477)/Homer	1e-196	-4.515e+02	0.0000	9873.0	15.06%
37	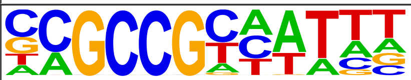	RRTF1(AP2EREBP)/colamp-RRTF1-DAP-Seq(GSE60143)/Homer	1e-188	-4.351e+02	0.0000	5225.0	7.97%
38	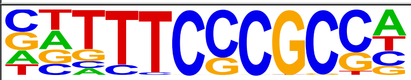	E2F7(E2F)/Hela-E2F7-ChIP-Seq(GSE32673)/Homer	1e-187	-4.315e+02	0.0000	2379.0	3.63%
39	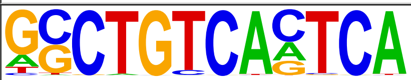	PBX1(Homeobox)/MCF7-PBX1-ChIP-Seq(GSE28007)/Homer	1e-185	-4.269e+02	0.0000	1800.0	2.75%
40	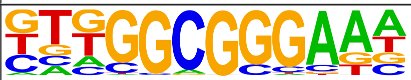	E2F3(E2F)/MEF-E2F3-ChIP-Seq(GSE71376)/Homer	1e-179	-4.130e+02	0.0000	10719.0	16.35%
41	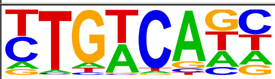	Tgif1(Homeobox)/mES-Tgif1-ChIP-Seq(GSE55404)/Homer	1e-160	-3.687e+02	0.0000	40421.0	61.66%
42	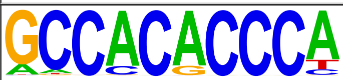	Klf4(Zf)/mES-Klf4-ChIP-Seq(GSE11431)/Homer	1e-152	-3.515e+02	0.0000	3886.0	5.93%
43	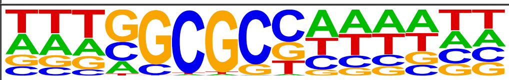	E2FA(E2FDP)/colamp-E2FA-DAP-Seq(GSE60143)/Homer	1e-152	-3.502e+02	0.0000	4833.0	7.37%
44	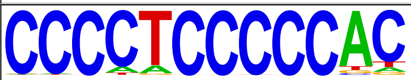	Zfp281(Zf)/ES-Zfp281-ChIP-Seq(GSE81042)/Homer	1e-146	-3.381e+02	0.0000	3370.0	5.14%
45	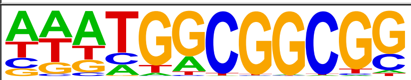	ERF9(AP2EREBP)/colamp-ERF9-DAP-Seq(GSE60143)/Homer	1e-137	-3.174e+02	0.0000	7959.0	12.14%

46	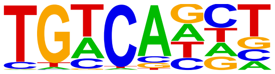	Tgif2(Homeobox)/mES-Tgif2-ChIP-Seq(GSE55404)/Homer	1e-134	-3.106e+02	0.0000	42535.0	64.88%
47	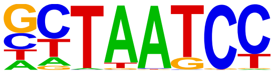	CRX(Homeobox)/Retina-Crx-ChIP-Seq(GSE20012)/Homer	1e-132	-3.043e+02	0.0000	29266.0	44.64%
48	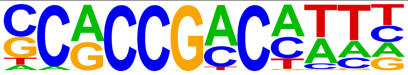	At5g65130(AP2EREBP)/colamp-At5g65130-DAP-Seq(GSE60143)/Homer	1e-127	-2.939e+02	0.0000	5130.0	7.83%
49	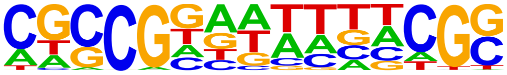	LOB(LOBAS2)/col-LOB-DAP-Seq(GSE60143)/Homer	1e-127	-2.930e+02	0.0000	5221.0	7.96%
50	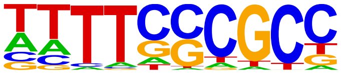	DEL2(E2FDP)/col-DEL2-DAP-Seq(GSE60143)/Homer	1e-118	-2.732e+02	0.0000	5603.0	8.55%
51	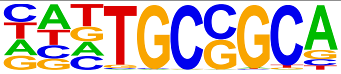	Zfp57(Zf)/H1-ZFP57.HA-ChIP-Seq(GSE115387)/Homer	1e-111	-2.558e+02	0.0000	9810.0	14.96%
52	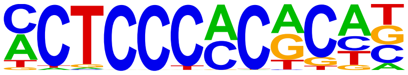	WT1(Zf)/Kidney-WT1-ChIP-Seq(GSE90016)/Homer	1e-109	-2.525e+02	0.0000	10217.0	15.59%
53	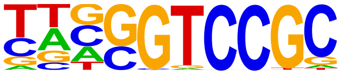	HINFP(Zf)/K562-HINFP.eGFP-ChIP-Seq(Encode)/Homer	1e-108	-2.490e+02	0.0000	5830.0	8.89%
54	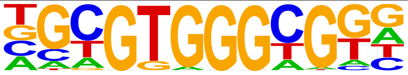	Egr2(Zf)/Thymocytes-Egr2-ChIP-Seq(GSE34254)/Homer	1e-107	-2.476e+02	0.0000	3312.0	5.05%
55	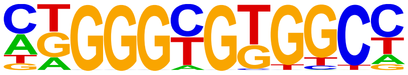	KLF6(Zf)/PDAC-KLF6-ChIP-Seq(GSE64557)/Homer	1e-97	-2.253e+02	0.0000	14737.0	22.48%
56	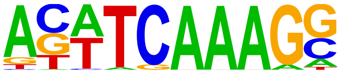	Tcf3(HMG)/mES-Tcf3-ChIP-Seq(GSE11724)/Homer	1e-97	-2.247e+02	0.0000	4561.0	6.96%
57	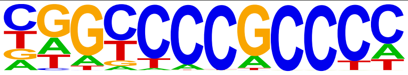	Sp2(Zf)/HEK293-Sp2.eGFP-ChIP-Seq(Encode)/Homer	1e-96	-2.218e+02	0.0000	19513.0	29.77%
58	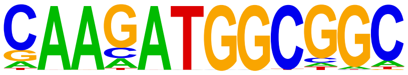	YY1(Zf)/Promoter/Homer	1e-96	-2.216e+02	0.0000	1814.0	2.77%
59	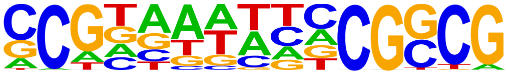	AS2(LOBAS2)/col-AS2-DAP-Seq(GSE60143)/Homer	1e-95	-2.188e+02	0.0000	2387.0	3.64%
60	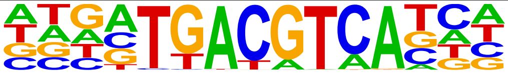	TGA5(bZIP)/col-TGA5-DAP-Seq(GSE60143)/Homer	1e-91	-2.116e+02	0.0000	1631.0	2.49%
61	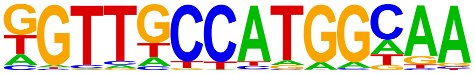	X-box(HTH)/NPC-H3K4me1-ChIP-Seq(GSE16256)/Homer	1e-91	-2.116e+02	0.0000	2083.0	3.18%
62	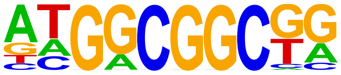	AT4G18450(AP2EREBP)/col-AT4G18450-DAP-Seq(GSE60143)/Homer	1e-88	-2.037e+02	0.0000	9805.0	14.96%
63	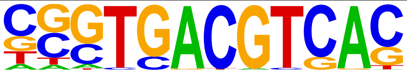	CRE(bZIP)/Promoter/Homer	1e-87	-2.017e+02	0.0000	4204.0	6.41%
64	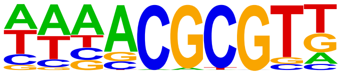	CAMTA1(CAMTA)/col-CAMTA1-DAP-Seq(GSE60143)/Homer	1e-85	-1.958e+02	0.0000	8099.0	12.35%
65	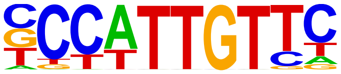	Sox2(HMG)/mES-Sox2-ChIP-Seq(GSE11431)/Homer	1e-83	-1.918e+02	0.0000	13016.0	19.85%
66	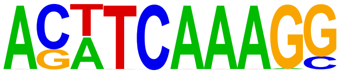	TCFL2(HMG)/K562-TCF7L2-ChIP-Seq(GSE29196)/Homer	1e-81	-1.878e+02	0.0000	1673.0	2.55%
67	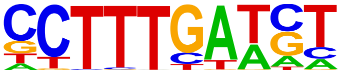	LEF1(HMG)/H1-LEF1-ChIP-Seq(GSE64758)/Homer	1e-80	-1.862e+02	0.0000	10647.0	16.24%
68	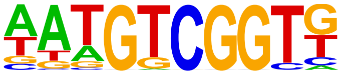	DREB19(AP2EREBP)/colamp-DREB19-DAP-Seq(GSE60143)/Homer	1e-80	-1.849e+02	0.0000	7275.0	11.10%
69	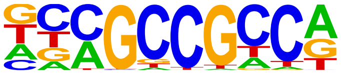	ERF5(AP2EREBP)/colamp-ERF5-DAP-Seq(GSE60143)/Homer	1e-80	-1.845e+02	0.0000	10184.0	15.53%
70	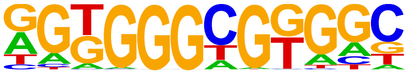	KLF14(Zf)/HEK293-KLF14.GFP-ChIP-Seq(GSE58341)/Homer	1e-79	-1.841e+02	0.0000	21932.0	33.46%

71	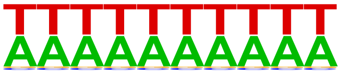	SeqBias: A/T bias	1e-79	-1.829e+02	0.0000	57774.0	88.13%
72	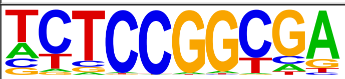	AT3G58630(Trihelix)/col-AT3G58630-DAP-Seq(GSE60143)/Homer	1e-79	-1.820e+02	0.0000	2755.0	4.20%
73	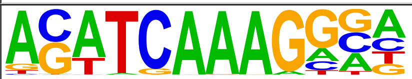	Tcf4(HMG)/Hct116-Tcf4-ChIP-Seq(SRA012054)/Homer	1e-78	-1.802e+02	0.0000	7523.0	11.48%
74	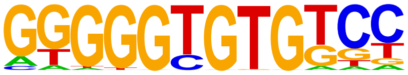	KLF10(Zf)/HEK293-KLF10.GFP-ChIP-Seq(GSE58341)/Homer	1e-77	-1.783e+02	0.0000	6890.0	10.51%
75	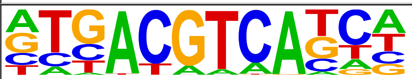	TGA4(bZIP)/colamp-TGA4-DAP-Seq(GSE60143)/Homer	1e-74	-1.707e+02	0.0000	4433.0	6.76%
76	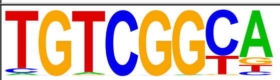	AT1G12630(AP2EREBP)/colamp-AT1G12630-DAP-Seq(GSE60143)/Homer	1e-73	-1.693e+02	0.0000	6241.0	9.52%
77	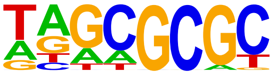	DPL-1(E2F)/cElegans-Adult-ChIP-Seq(modEncode)/Homer	1e-73	-1.687e+02	0.0000	11143.0	17.00%
78	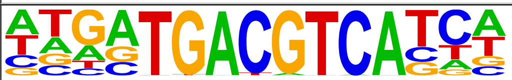	TGA3(bZIP)/colamp-TGA3-DAP-Seq(GSE60143)/Homer	1e-73	-1.684e+02	0.0000	1050.0	1.60%
79	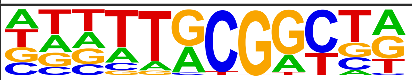	At5g08750(C3H)/col-At5g08750-DAP-Seq(GSE60143)/Homer	1e-73	-1.681e+02	0.0000	6796.0	10.37%
80	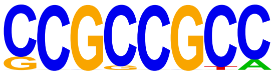	SHN3(AP2EREBP)/col-SHN3-DAP-Seq(GSE60143)/Homer	1e-71	-1.648e+02	0.0000	9682.0	14.77%
81	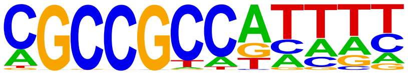	RAP212(AP2EREBP)/col-RAP212-DAP-Seq(GSE60143)/Homer	1e-69	-1.607e+02	0.0000	12162.0	18.55%
82	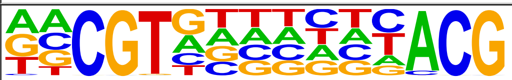	ANAC094(NAC)/col-ANAC094-DAP-Seq(GSE60143)/Homer	1e-67	-1.553e+02	0.0000	6768.0	10.32%
83	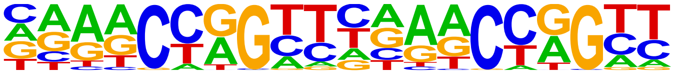	Tcfcp2l1(CP2)/mES-Tcfcp2l1-ChIP-Seq(GSE11431)/Homer	1e-66	-1.521e+02	0.0000	2238.0	3.41%
84	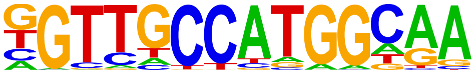	Rfx1(HTH)/NPC-H3K4me1-ChIP-Seq(GSE16256)/Homer	1e-65	-1.513e+02	0.0000	3518.0	5.37%
85	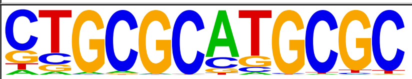	NRF1(NRF)/MCF7-NRF1-ChIP-Seq(Unpublished)/Homer	1e-65	-1.505e+02	0.0000	2523.0	3.85%
86	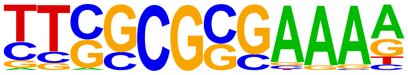	E2F(E2F)/Hela-CellCycle-Expression/Homer	1e-63	-1.471e+02	0.0000	823.0	1.26%
87	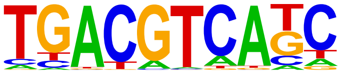	TGA1(bZIP)/colamp-TGA1-DAP-Seq(GSE60143)/Homer	1e-62	-1.435e+02	0.0000	5846.0	8.92%
88	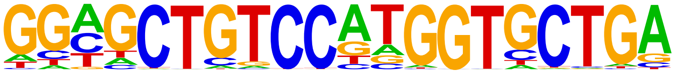	REST-NRSF(Zf)/Jurkat-NRSF-ChIP-Seq/Homer	1e-60	-1.400e+02	0.0000	299.0	0.46%
89	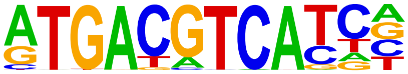	JunD(bZIP)/K562-JunD-ChIP-Seq/Homer	1e-60	-1.385e+02	0.0000	1478.0	2.25%
90	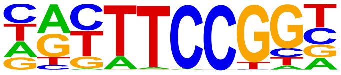	Elk4(ETS)/Hela-Elk4-ChIP-Seq(GSE31477)/Homer	1e-60	-1.382e+02	0.0000	6826.0	10.41%
91	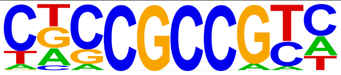	LEP(AP2EREBP)/col-LEP-DAP-Seq(GSE60143)/Homer	1e-59	-1.371e+02	0.0000	9299.0	14.18%
92	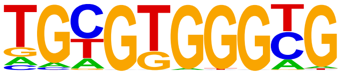	Egr1(Zf)/K562-Egr1-ChIP-Seq(GSE32465)/Homer	1e-59	-1.369e+02	0.0000	10300.0	15.71%
93	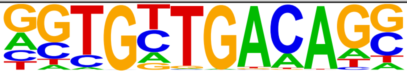	Tbx20(T-box)/Heart-Tbx20-ChIP-Seq(GSE29636)/Homer	1e-57	-1.332e+02	0.0000	5048.0	7.70%
94	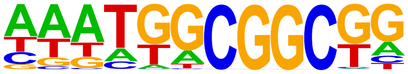	ABR1(AP2EREBP)/colamp-ABR1-DAP-Seq(GSE60143)/Homer	1e-57	-1.325e+02	0.0000	14302.0	21.82%
95	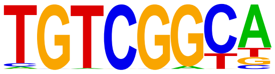	Rap210(AP2EREBP)/col-Rap210-DAP-Seq(GSE60143)/Homer	1e-56	-1.302e+02	0.0000	6646.0	10.14%

96	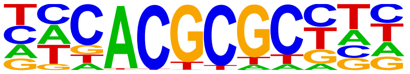	FHY3(FAR1)/Arabidopsis-FHY3-ChIP-Seq(GSE30711)/Homer	1e-56	-1.294e+02	0.0000	5633.0	8.59%
97	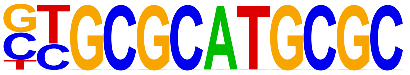	NRF(NRF)/Promoter/Homer	1e-55	-1.281e+02	0.0000	2826.0	4.31%
98	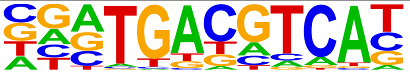	Atf2(bZIP)/3T3L1-Atf2-ChIP-Seq(GSE56872)/Homer	1e-55	-1.272e+02	0.0000	4635.0	7.07%
99	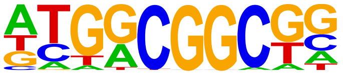	ERF3(AP2EREBP)/colamp-ERF3-DAP-Seq(GSE60143)/Homer	1e-55	-1.267e+02	0.0000	11992.0	18.29%
100	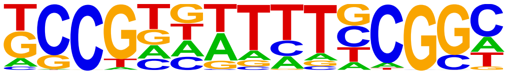	LBD13(LOBAS2)/colamp-LBD13-DAP-Seq(GSE60143)/Homer	1e-54	-1.260e+02	0.0000	12439.0	18.97%
101	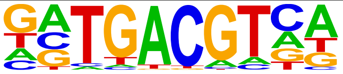	bZIP50(bZIP)/colamp-bZIP50-DAP-Seq(GSE60143)/Homer	1e-54	-1.256e+02	0.0000	13339.0	20.35%
102	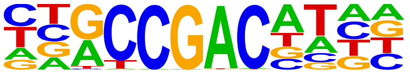	CBF3(AP2EREBP)/colamp-CBF3-DAP-Seq(GSE60143)/Homer	1e-54	-1.246e+02	0.0000	4860.0	7.41%
103	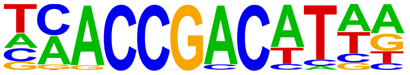	DREB2(AP2EREBP)/col-DREB2-DAP-Seq(GSE60143)/Homer	1e-54	-1.244e+02	0.0000	5507.0	8.40%
104	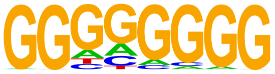	Maz(Zf)/HepG2-Maz-ChIP-Seq(GSE31477)/Homer	1e-53	-1.239e+02	0.0000	19125.0	29.17%
105	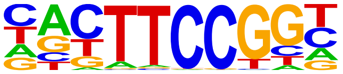	Elk1(ETS)/Hela-Elk1-ChIP-Seq(GSE31477)/Homer	1e-52	-1.219e+02	0.0000	6697.0	10.22%
106	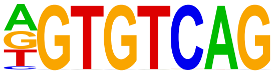	CELF2(RRM)/JSL1-CELF2-CLIP-Seq(GSE71264)/Homer	1e-52	-1.210e+02	0.0000	6494.0	9.91%
107	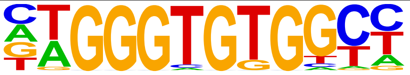	EKLF(Zf)/Erythrocyte-Klf1-ChIP-Seq(GSE20478)/Homer	1e-52	-1.207e+02	0.0000	1634.0	2.49%
108	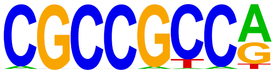	CRF4(AP2EREBP)/colamp-CRF4-DAP-Seq(GSE60143)/Homer	1e-51	-1.178e+02	0.0000	11038.0	16.84%
109	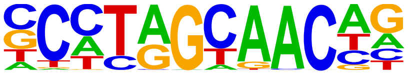	Rfx5(HTH)/GM12878-Rfx5-ChIP-Seq(GSE31477)/Homer	1e-49	-1.144e+02	0.0000	4860.0	7.41%
110	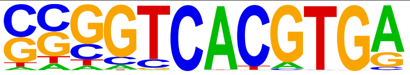	E-box(bHLH)/Promoter/Homer	1e-49	-1.131e+02	0.0000	1314.0	2.00%
111	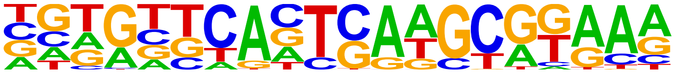	PAX6(Paired,Homeobox)/Forebrain-Pax6-ChIP-Seq(GSE66961)/Homer	1e-47	-1.096e+02	0.0000	1731.0	2.64%
112	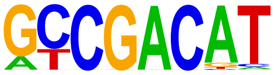	DDF1(AP2EREBP)/col-DDF1-DAP-Seq(GSE60143)/Homer	1e-47	-1.089e+02	0.0000	4199.0	6.41%
113	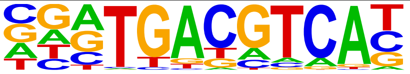	Atf7(bZIP)/3T3L1-Atf7-ChIP-Seq(GSE56872)/Homer	1e-47	-1.087e+02	0.0000	6674.0	10.18%
114	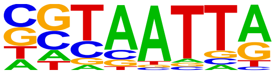	DLX5(Homeobox)/BasalGanglia-Dlx5-ChIP-seq(GSE124936)/Homer	1e-44	-1.033e+02	0.0000	13172.0	20.09%
115	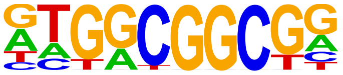	ERF10(AP2EREBP)/col-ERF10-DAP-Seq(GSE60143)/Homer	1e-44	-1.032e+02	0.0000	11969.0	18.26%
116	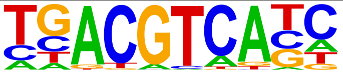	TGA6(bZIP)/colamp-TGA6-DAP-Seq(GSE60143)/Homer	1e-44	-1.018e+02	0.0000	8335.0	12.71%
117	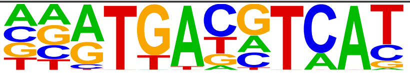	CREB5(bZIP)/LNCaP-CREB5.V5-ChIP-Seq(GSE137775)/Homer	1e-44	-1.015e+02	0.0000	5333.0	8.14%
118	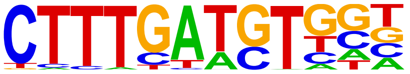	Tcf7(HMG)/GM12878-TCF7-ChIP-Seq(Encode)/Homer	1e-42	-9.812e+01	0.0000	5568.0	8.49%
119	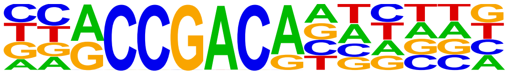	AT3G60490(AP2EREBP)/colamp-AT3G60490-DAP-Seq(GSE60143)/Homer	1e-42	-9.770e+01	0.0000	3581.0	5.46%
120		ERF1(AP2EREBP)/colamp-ERF1-DAP-Seq(GSE60143)/Homer	1e-42	-9.675e+01	0.0000	11945.0	18.22%

	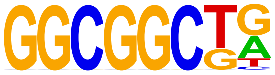						
121	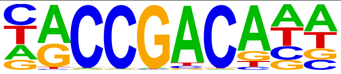	At4g16750(AP2EREBP)/col-At4g16750-DAP-Seq(GSE60143)/Homer	1e-41	-9.449e+01	0.0000	7962.0	12.15%
122	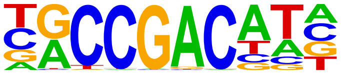	CBF1(AP2EREBP)/colamp-CBF1-DAP-Seq(GSE60143)/Homer	1e-40	-9.317e+01	0.0000	7058.0	10.77%
123	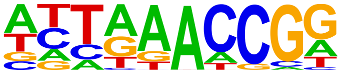	Unknown3/Arabidopsis-Promoters/Homer	1e-40	-9.257e+01	0.0000	2139.0	3.26%
124	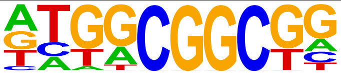	ERF11(AP2EREBP)/col-ERF11-DAP-Seq(GSE60143)/Homer	1e-39	-9.163e+01	0.0000	13574.0	20.71%
125	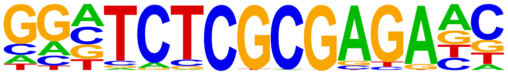	ZBTB33(Zf)/GM12878-ZBTB33-ChIP-Seq(GSE32465)/Homer	1e-39	-8.993e+01	0.0000	767.0	1.17%
126	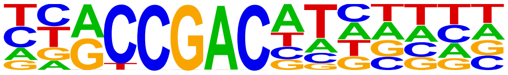	CBF2(AP2EREBP)/colamp-CBF2-DAP-Seq(GSE60143)/Homer	1e-38	-8.969e+01	0.0000	4822.0	7.36%
127	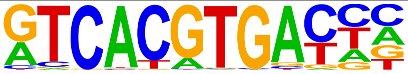	TFE3(bHLH)/MEF-TFE3-ChIP-Seq(GSE75757)/Homer	1e-38	-8.954e+01	0.0000	1018.0	1.55%
128	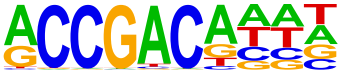	AT1G77200(AP2EREBP)/colamp-AT1G77200-DAP-Seq(GSE60143)/Homer	1e-38	-8.932e+01	0.0000	7957.0	12.14%
129	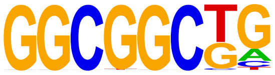	ERF2(AP2EREBP)/colamp-ERF2-DAP-Seq(GSE60143)/Homer	1e-38	-8.773e+01	0.0000	12660.0	19.31%
130	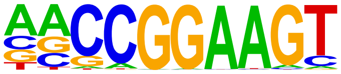	ETS(ETS)/Promoter/Homer	1e-37	-8.710e+01	0.0000	3569.0	5.44%
131	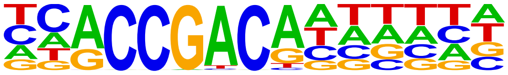	AT3G16280(AP2EREBP)/colamp-AT3G16280-DAP-Seq(GSE60143)/Homer	1e-37	-8.578e+01	0.0000	4029.0	6.15%
132	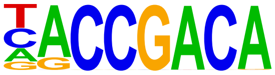	bHLH10(bHLH)/colamp-bHLH10-DAP-Seq(GSE60143)/Homer	1e-36	-8.323e+01	0.0000	3571.0	5.45%
133	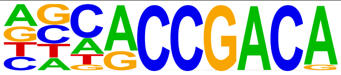	TINY(AP2EREBP)/col-TINY-DAP-Seq(GSE60143)/Homer	1e-35	-8.116e+01	0.0000	3326.0	5.07%
134	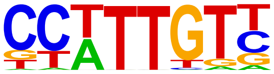	Sox3(HMG)/NPC-Sox3-ChIP-Seq(GSE33059)/Homer	1e-34	-8.029e+01	0.0000	23264.0	35.49%
135	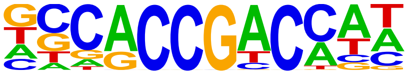	At4g28140(AP2EREBP)/colamp-At4g28140-DAP-Seq(GSE60143)/Homer	1e-34	-7.967e+01	0.0000	4459.0	6.80%
136	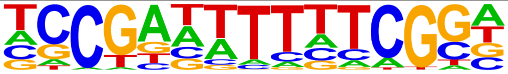	LBD2(LOBAS2)/colamp-LBD2-DAP-Seq(GSE60143)/Homer	1e-34	-7.937e+01	0.0000	3435.0	5.24%
137	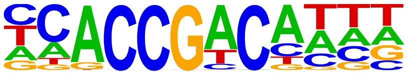	DEAR2(AP2EREBP)/colamp-DEAR2-DAP-Seq(GSE60143)/Homer	1e-34	-7.928e+01	0.0000	12190.0	18.59%
138	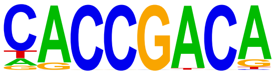	ERF38(AP2EREBP)/col-ERF38-DAP-Seq(GSE60143)/Homer	1e-34	-7.925e+01	0.0000	5828.0	8.89%
139	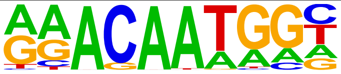	Sox15(HMG)/CPA-Sox15-ChIP-Seq(GSE62909)/Homer	1e-34	-7.902e+01	0.0000	14121.0	21.54%
140	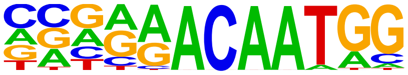	Sox7(HMG)/ESC-Sox7-ChIP-Seq(GSE133899)/Homer	1e-33	-7.737e+01	0.0000	4009.0	6.12%
141	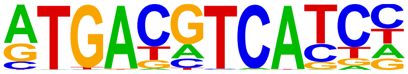	c-Jun-CRE(bZIP)/K562-cJun-ChIP-Seq(GSE31477)/Homer	1e-33	-7.729e+01	0.0000	4250.0	6.48%
142	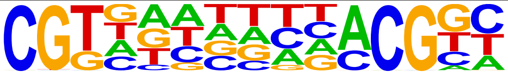	ANAC042(NAC)/col-ANAC042-DAP-Seq(GSE60143)/Homer	1e-33	-7.723e+01	0.0000	11711.0	17.86%
143	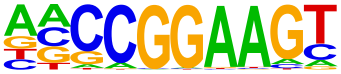	ELF1(ETS)/Jurkat-ELF1-ChIP-Seq(SRA014231)/Homer	1e-33	-7.630e+01	0.0000	5784.0	8.82%
144	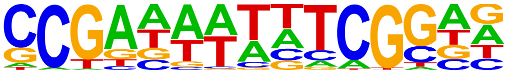	CDM1(C3H)/colamp-CDM1-DAP-Seq(GSE60143)/Homer	1e-33	-7.617e+01	0.0000	1112.0	1.70%

145	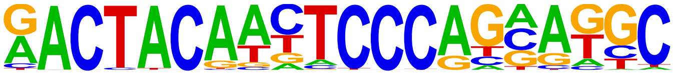	Ronin(THAP)/ES-Thap11-ChIP-Seq(GSE51522)/Homer	1e-32	-7.564e+01	0.0000	569.0	0.87%
146	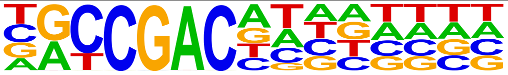	CBF4(AP2EREBP)/colamp-CBF4-DAP-Seq(GSE60143)/Homer	1e-32	-7.511e+01	0.0000	6800.0	10.37%
147	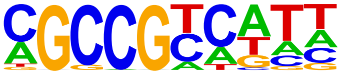	ERF8(AP2EREBP)/colamp-ERF8-DAP-Seq(GSE60143)/Homer	1e-32	-7.460e+01	0.0000	14675.0	22.39%
148	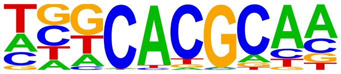	Arnt:Ahr(bHLH)/MCF7-Arnt-ChIP-Seq(Lo_et_al.)/Homer	1e-32	-7.425e+01	0.0000	12813.0	19.55%
149	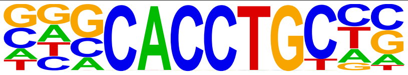	Slug(Zf)/Mesoderm-Snai2-ChIP-Seq(GSE61475)/Homer	1e-31	-7.302e+01	0.0000	9711.0	14.81%
150	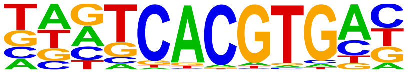	BIM3(bHLH)/col-BIM3-DAP-Seq(GSE60143)/Homer	1e-31	-7.209e+01	0.0000	2038.0	3.11%
151	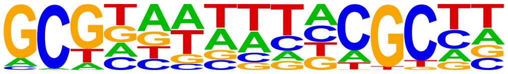	LBD23(LOBAS2)/colamp-LBD23-DAP-Seq(GSE60143)/Homer	1e-31	-7.179e+01	0.0000	15795.0	24.09%
152	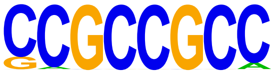	ERF73(AP2EREBP)/col-ERF73-DAP-Seq(GSE60143)/Homer	1e-30	-7.135e+01	0.0000	12933.0	19.73%
153	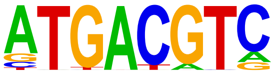	TGA10(bZIP)/colamp-TGA10-DAP-Seq(GSE60143)/Homer	1e-30	-7.032e+01	0.0000	7958.0	12.14%
154	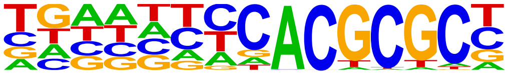	FAR1(FAR1)/col-FAR1-DAP-Seq(GSE60143)/Homer	1e-30	-6.982e+01	0.0000	2323.0	3.54%
155	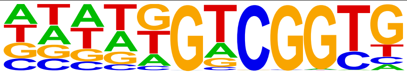	At1g36060(AP2EREBP)/colamp-At1g36060-DAP-Seq(GSE60143)/Homer	1e-29	-6.741e+01	0.0000	8136.0	12.41%
156	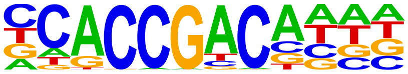	At1g22810(AP2EREBP)/colamp-At1g22810-DAP-Seq(GSE60143)/Homer	1e-28	-6.646e+01	0.0000	5627.0	8.58%
157	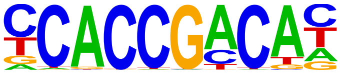	DREB26(AP2EREBP)/col-DREB26-DAP-Seq(GSE60143)/Homer	1e-28	-6.639e+01	0.0000	4115.0	6.28%
158	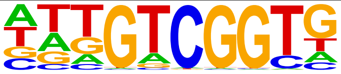	CEJ1(AP2EREBP)/col-CEJ1-DAP-Seq(GSE60143)/Homer	1e-28	-6.629e+01	0.0000	10751.0	16.40%
159	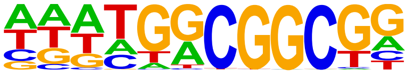	ERF4(AP2EREBP)/colamp-ERF4-DAP-Seq(GSE60143)/Homer	1e-27	-6.431e+01	0.0000	14765.0	22.52%
160	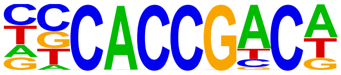	AT1G44830(AP2EREBP)/col-AT1G44830-DAP-Seq(GSE60143)/Homer	1e-27	-6.356e+01	0.0000	5258.0	8.02%
161	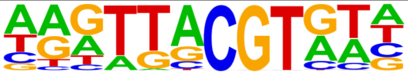	NAP(NAC)/col-NAP-DAP-Seq(GSE60143)/Homer	1e-27	-6.338e+01	0.0000	11852.0	18.08%
162	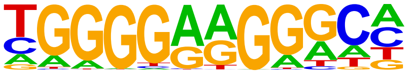	ZNF467(Zf)/HEK293-ZNF467.GFP-ChIP-Seq(GSE58341)/Homer	1e-27	-6.270e+01	0.0000	14572.0	22.23%
163	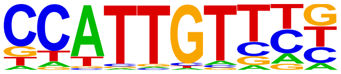	Sox17(HMG)/Endoderm-Sox17-ChIP-Seq(GSE61475)/Homer	1e-27	-6.229e+01	0.0000	9345.0	14.25%
164	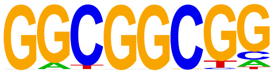	ESE1(AP2EREBP)/col-ESE1-DAP-Seq(GSE60143)/Homer	1e-26	-6.141e+01	0.0000	13116.0	20.01%
165	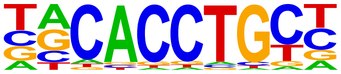	Snail1(Zf)/LS174T-SNAIL1.HA-ChIP-Seq(GSE127183)/Homer	1e-26	-6.075e+01	0.0000	14168.0	21.61%
166	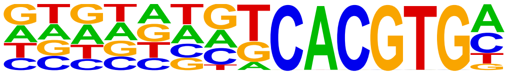	BIM1(bHLH)/colamp-BIM1-DAP-Seq(GSE60143)/Homer	1e-25	-5.970e+01	0.0000	1895.0	2.89%
167	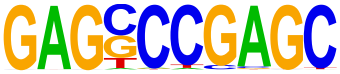	ZNF519(Zf)/HEK293-ZNF519.GFP-ChIP-Seq(GSE58341)/Homer	1e-25	-5.896e+01	0.0000	4176.0	6.37%
168	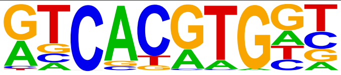	Usf2(bHLH)/C2C12-Usf2-ChIP-Seq(GSE36030)/Homer	1e-25	-5.846e+01	0.0000	4277.0	6.52%
169	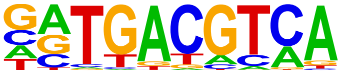	Atf1(bZIP)/K562-ATF1-ChIP-Seq(GSE31477)/Homer	1e-25	-5.846e+01	0.0000	9145.0	13.95%

170	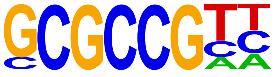	PUCHI(AP2EREBP)/colamp-PUCHI-DAP-Seq(GSE60143)/Homer	1e-25	-5.815e+01	0.0000	12642.0	19.28%
171	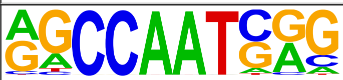	NFY(CCAAT)/Promoter/Homer	1e-24	-5.665e+01	0.0000	9423.0	14.37%
172	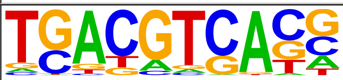	FEA4(bZIP)/Corn-FEA4-ChIP-Seq(GSE61954)/Homer	1e-24	-5.533e+01	0.0000	15186.0	23.16%
173	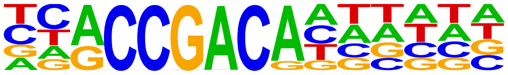	At2g44940(AP2EREBP)/colamp-At2g44940-DAP-Seq(GSE60143)/Homer	1e-24	-5.531e+01	0.0000	2500.0	3.81%
174	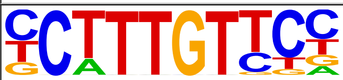	Sox4(HMG)/proB-Sox4-ChIP-Seq(GSE50066)/Homer	1e-23	-5.476e+01	0.0000	12684.0	19.35%
175	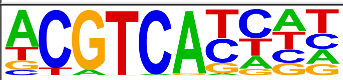	TGA2(bZIP)/colamp-TGA2-DAP-Seq(GSE60143)/Homer	1e-23	-5.410e+01	0.0000	7610.0	11.61%
176	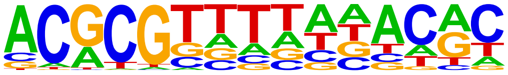	CAMTA5(CAMTA)/col-CAMTA5-DAP-Seq(GSE60143)/Homer	1e-23	-5.396e+01	0.0000	4414.0	6.73%
177	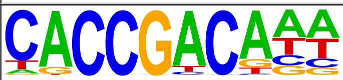	At4g31060(AP2EREBP)/colamp-At4g31060-DAP-Seq(GSE60143)/Homer	1e-23	-5.338e+01	0.0000	4783.0	7.30%
178	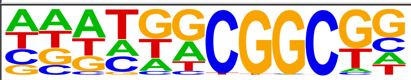	RAP26(AP2EREBP)/colamp-RAP26-DAP-Seq(GSE60143)/Homer	1e-23	-5.317e+01	0.0000	16001.0	24.41%
179	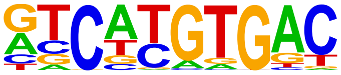	MITF(bHLH)/MastCells-MITF-ChIP-Seq(GSE48085)/Homer	1e-22	-5.194e+01	0.0000	11075.0	16.89%
180	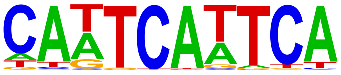	WUS1(Homeobox)/colamp-WUS1-DAP-Seq(GSE60143)/Homer	1e-21	-4.895e+01	0.0000	5556.0	8.48%
181	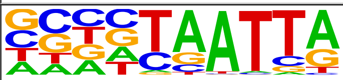	DLX1(Homeobox)/BasalGanglia-Dlx1-ChIP-seq(GSE124936)/Homer	1e-21	-4.860e+01	0.0000	19445.0	29.66%
182	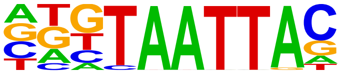	Dlx3(Homeobox)/Kerainocytes-Dlx3-ChIP-Seq(GSE89884)/Homer	1e-20	-4.774e+01	0.0000	11342.0	17.30%
183	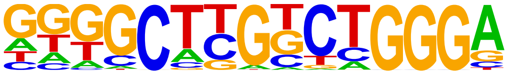	Zfp809(Zf)/ES-Zfp809-ChIP-Seq(GSE70799)/Homer	1e-20	-4.724e+01	0.0000	4059.0	6.19%
184	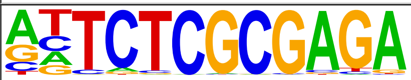	GFX(?)/Promoter/Homer	1e-19	-4.565e+01	0.0000	263.0	0.40%
185	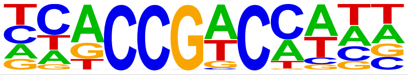	At1g19210(AP2EREBP)/colamp-At1g19210-DAP-Seq(GSE60143)/Homer	1e-19	-4.456e+01	0.0000	14017.0	21.38%
186	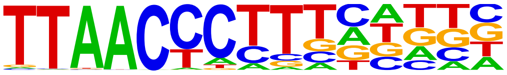	ZNF652/HepG2-ZNF652.Flag-ChIP-Seq(Encode)/Homer	1e-18	-4.365e+01	0.0000	3438.0	5.24%
187	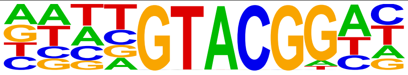	bHLH28(bHLH)/col-bHLH28-DAP-Seq(GSE60143)/Homer	1e-18	-4.158e+01	0.0000	4565.0	6.96%
188	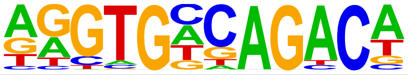	Tbox:Smad(T-box,MAD)/ESCd5-Smad2_3-ChIP-Seq(GSE29422)/Homer	1e-17	-4.112e+01	0.0000	2489.0	3.80%
189	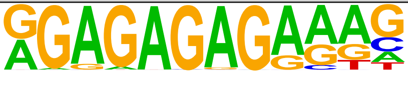	FRS9(ND)/col-FRS9-DAP-Seq(GSE60143)/Homer	1e-17	-4.088e+01	0.0000	3382.0	5.16%
190	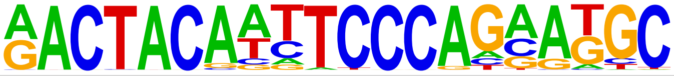	GFY-Staf(?,Zf)/Promoter/Homer	1e-17	-4.066e+01	0.0000	766.0	1.17%
191	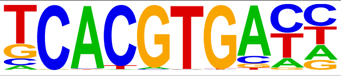	Cbf1(bHLH)/Yeast-Cbf1-ChIP-Seq(GSE29506)/Homer	1e-17	-4.009e+01	0.0000	4254.0	6.49%
192	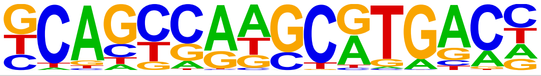	PAX5(Paired,Homeobox)/GM12878-PAX5-ChIP-Seq(GSE32465)/Homer	1e-17	-3.950e+01	0.0000	6786.0	10.35%
193	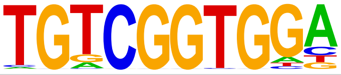	At1g77640(AP2EREBP)/col-At1g77640-DAP-Seq(GSE60143)/Homer	1e-16	-3.861e+01	0.0000	2799.0	4.27%
194	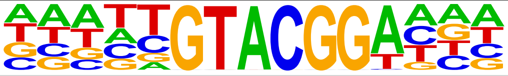	SPL14(SBP)/col-SPL14-DAP-Seq(GSE60143)/Homer	1e-16	-3.810e+01	0.0000	2731.0	4.17%

195	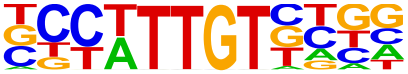	Sox21(HMG)/ESC-SOX21-ChIP-Seq(GSE110505)/Homer	1e-16	-3.746e+01	0.0000	24065.0	36.71%
196	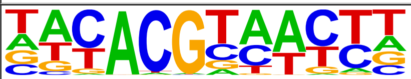	ANAC047(NAC)/colamp-ANAC047-DAP-Seq(GSE60143)/Homer	1e-15	-3.642e+01	0.0000	9314.0	14.21%
197	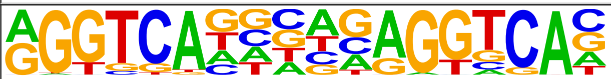	RAR:RXR(NR),DR5/ES-RAR-ChIP-Seq(GSE56893)/Homer	1e-15	-3.462e+01	0.0000	399.0	0.61%
198	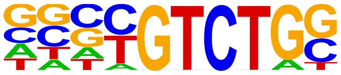	Smad4(MAD)/ESC-SMAD4-ChIP-Seq(GSE29422)/Homer	1e-14	-3.443e+01	0.0000	21865.0	33.35%
199	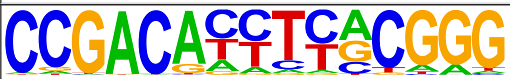	GEI-11(Myb?)/cElegans-L4-GEI11-ChIP-Seq(modEncode)/Homer	1e-14	-3.422e+01	0.0000	1000.0	1.53%
200	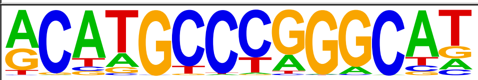	p53(p53)/mES-cMyc-ChIP-Seq(GSE11431)/Homer	1e-14	-3.385e+01	0.0000	275.0	0.42%
201	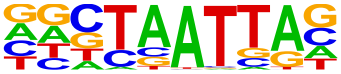	En1(Homeobox)/SUM149-EN1-ChIP-Seq(GSE120957)/Homer	1e-14	-3.355e+01	0.0000	24552.0	37.45%
202	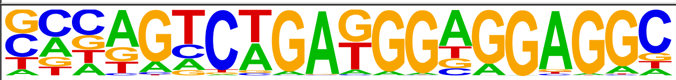	ZSCAN22(Zf)/HEK293-ZSCAN22.GFP-ChIP-Seq(GSE58341)/Homer	1e-14	-3.281e+01	0.0000	1056.0	1.61%
203	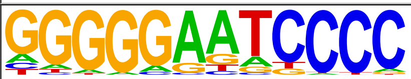	NFkB-p50,p52(RHD)/Monocyte-p50-ChIP-Chip(Schreiber_et_al.)/Homer	1e-13	-3.149e+01	0.0000	2345.0	3.58%
204	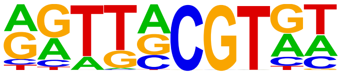	NAM(NAC)/col-NAM-DAP-Seq(GSE60143)/Homer	1e-13	-3.126e+01	0.0000	15540.0	23.70%
205	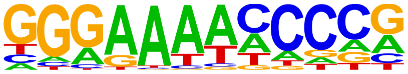	Dorsal(RHD)/Embryo-dl-ChIP-Seq(GSE65441)/Homer	1e-13	-3.036e+01	0.0000	2860.0	4.36%
206	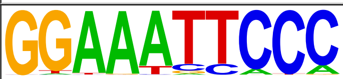	NFkB-p65-Rel(RHD)/ThioMac-LPS-Expression(GSE23622)/Homer	1e-11	-2.745e+01	0.0000	867.0	1.32%
207	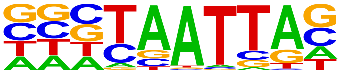	DLX2(Homeobox)/BasalGanglia-Dlx2-ChIP-seq(GSE124936)/Homer	1e-11	-2.719e+01	0.0000	20933.0	31.93%
208	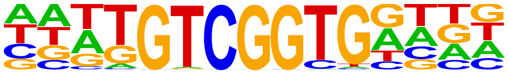	DEAR5(AP2EREBP)/col-DEAR5-DAP-Seq(GSE60143)/Homer	1e-11	-2.617e+01	0.0000	2095.0	3.20%
209	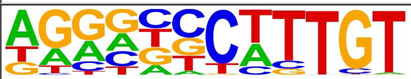	Sox9(HMG)/Limb-SOX9-ChIP-Seq(GSE73225)/Homer	1e-11	-2.595e+01	0.0000	11873.0	18.11%
210	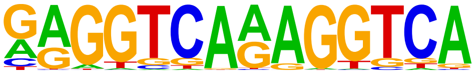	TR4(NR),DR1/Hela-TR4-ChIP-Seq(GSE24685)/Homer	1e-11	-2.560e+01	0.0000	971.0	1.48%
211	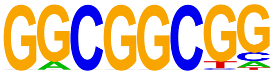	ERF104(AP2EREBP)/col-ERF104-DAP-Seq(GSE60143)/Homer	1e-10	-2.442e+01	0.0000	14638.0	22.33%
212	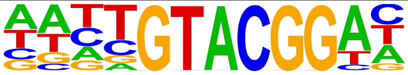	SPL13(SBP)/col-SPL13-DAP-Seq(GSE60143)/Homer	1e-10	-2.392e+01	0.0000	1545.0	2.36%
213	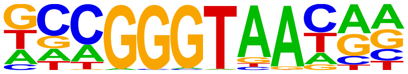	REB1/SacCer-Promoters/Homer	1e-10	-2.365e+01	0.0000	2351.0	3.59%
214	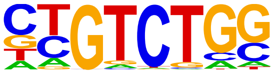	Smad2(MAD)/ES-SMAD2-ChIP-Seq(GSE29422)/Homer	1e-9	-2.281e+01	0.0000	21520.0	32.83%
215	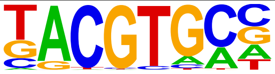	HIF-1a(bHLH)/MCF7-HIF1a-ChIP-Seq(GSE28352)/Homer	1e-9	-2.237e+01	0.0000	5840.0	8.91%
216	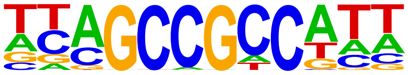	ERF13(AP2EREBP)/colamp-ERF13-DAP-Seq(GSE60143)/Homer	1e-9	-2.209e+01	0.0000	17044.0	26.00%
217	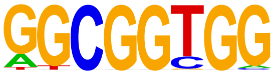	AT3G57600(AP2EREBP)/col-AT3G57600-DAP-Seq(GSE60143)/Homer	1e-9	-2.138e+01	0.0000	13820.0	21.08%
218	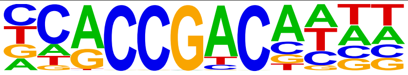	RAP21(AP2EREBP)/colamp-RAP21-DAP-Seq(GSE60143)/Homer	1e-9	-2.088e+01	0.0000	2064.0	3.15%
219	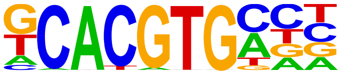	bHLHE40(bHLH)/HepG2-BHLHE40-ChIP-Seq(GSE31477)/Homer	1e-9	-2.084e+01	0.0000	5538.0	8.45%

220	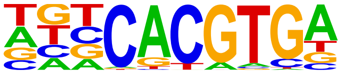	SPCH(bHLH)/Seedling-SPCH-ChIP-Seq(GSE57497)/Homer	1e-8	-2.061e+01	0.0000	15134.0	23.09%
221	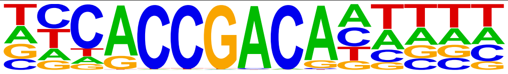	At4g32800(AP2EREBP)/colamp-At4g32800-DAP-Seq(GSE60143)/Homer	1e-8	-2.008e+01	0.0000	1339.0	2.04%
222	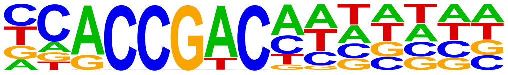	DEAR3(AP2EREBP)/colamp-DEAR3-DAP-Seq(GSE60143)/Homer	1e-8	-2.004e+01	0.0000	3476.0	5.30%
223	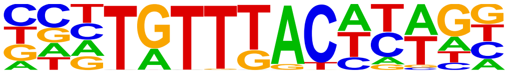	Foxa3(Forkhead)/Liver-Foxa3-ChIP-Seq(GSE77670)/Homer	1e-8	-1.995e+01	0.0000	4315.0	6.58%
224	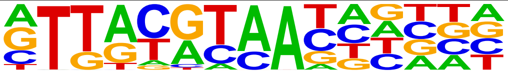	NFIL3(bZIP)/HepG2-NFIL3-ChIP-Seq(Encode)/Homer	1e-8	-1.945e+01	0.0000	8842.0	13.49%
225	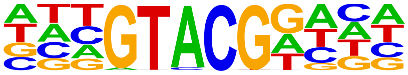	SPL5(SBP)/colamp-SPL5-DAP-Seq(GSE60143)/Homer	1e-8	-1.885e+01	0.0000	7148.0	10.90%
226	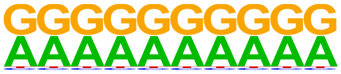	SeqBias: G/A bias	1e-8	-1.855e+01	0.0000	65524.0	99.95%
227	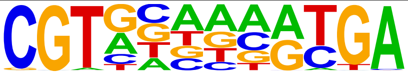	ABF1/SacCer-Promoters/Homer	1e-8	-1.848e+01	0.0000	3266.0	4.98%
228	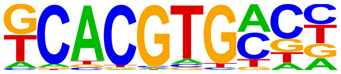	bHLHE41(bHLH)/proB-Bhlhe41-ChIP-Seq(GSE93764)/Homer	1e-8	-1.847e+01	0.0000	17735.0	27.05%
229	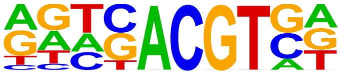	HY5(bZIP)/colamp-HY5-DAP-Seq(GSE60143)/Homer	1e-7	-1.656e+01	0.0000	10980.0	16.75%
230	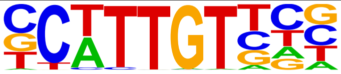	Sox10(HMG)/SciaticNerve-Sox3-ChIP-Seq(GSE35132)/Homer	1e-7	-1.651e+01	0.0000	22613.0	34.49%
231	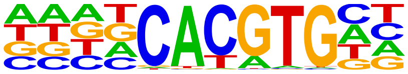	BIM2(bHLH)/col-BIM2-DAP-Seq(GSE60143)/Homer	1e-6	-1.521e+01	0.0000	12508.0	19.08%
232	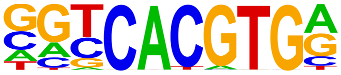	USF1(bHLH)/GM12878-Usf1-ChIP-Seq(GSE32465)/Homer	1e-6	-1.511e+01	0.0000	6887.0	10.51%
233	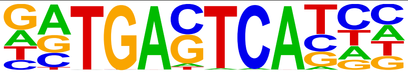	Jun-AP1(bZIP)/K562-cJun-ChIP-Seq(GSE31477)/Homer	1e-6	-1.468e+01	0.0000	2605.0	3.97%
234	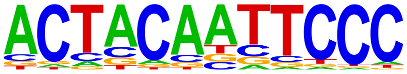	GFY(?)/Promoter/Homer	1e-6	-1.448e+01	0.0000	953.0	1.45%
235	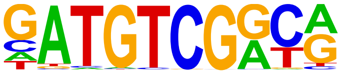	DDF2(AP2EREBP)/col-DDF2-DAP-Seq(GSE60143)/Homer	1e-6	-1.426e+01	0.0000	426.0	0.65%
236	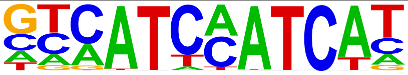	HOXA2(Homeobox)/mES-Hoxa2-ChIP-Seq(Donaldson_et_al.)/Homer	1e-6	-1.419e+01	0.0000	1383.0	2.11%
237	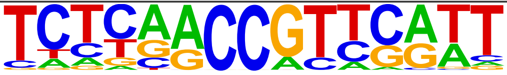	AT5G59990(C2C2COlike)/colamp-AT5G59990-DAP-Seq(GSE60143)/Homer	1e-6	-1.400e+01	0.0000	251.0	0.38%
238	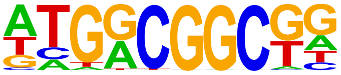	ERF7(AP2EREBP)/col-ERF7-DAP-Seq(GSE60143)/Homer	1e-5	-1.295e+01	0.0000	16565.0	25.27%
239	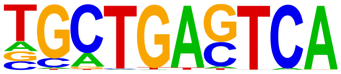	Bach2(bZIP)/OCILy7-Bach2-ChIP-Seq(GSE44420)/Homer	1e-5	-1.291e+01	0.0000	2380.0	3.63%
240	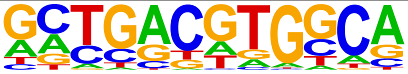	O2(bZIP)/Corn-O2-ChIP-Seq(GSE63991)/Homer	1e-5	-1.284e+01	0.0000	2330.0	3.55%
241	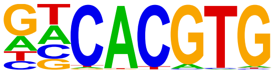	CLOCK(bHLH)/Liver-Clock-ChIP-Seq(GSE39860)/Homer	1e-5	-1.280e+01	0.0000	8301.0	12.66%
242	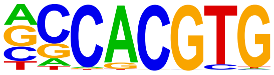	c-Myc(bHLH)/LNCAP-cMyc-ChIP-Seq(Unpublished)/Homer	1e-5	-1.264e+01	0.0000	9046.0	13.80%
243	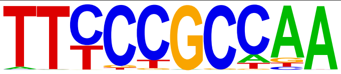	DEL1(E2FDP)/colamp-DEL1-DAP-Seq(GSE60143)/Homer	1e-5	-1.250e+01	0.0000	48.0	0.07%
244		Pax8(Paired,Homeobox)/Thyroid-Pax8-ChIP-Seq(GSE26938)/Homer	1e-5	-1.198e+01	0.0000	4890.0	7.46%

	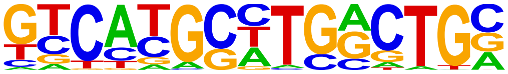						
245	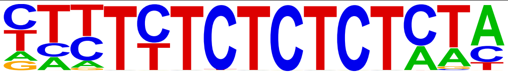	BPC6(BBRBPC)/col-BPC6-DAP-Seq(GSE60143)/Homer	1e-5	-1.193e+01	0.0000	450.0	0.69%
246	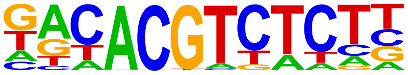	bHLH157(bHLH)/col-bHLH157-DAP-Seq(GSE60143)/Homer	1e-5	-1.164e+01	0.0000	3131.0	4.78%
247	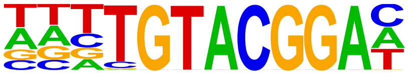	SPL3(SBP)/colamp-SPL3-DAP-Seq(GSE60143)/Homer	1e-4	-1.142e+01	0.0000	647.0	0.99%
248	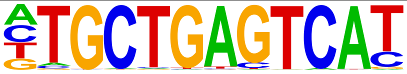	Nrf2(bZIP)/Lymphoblast-Nrf2-ChIP-Seq(GSE37589)/Homer	1e-4	-1.098e+01	0.0001	662.0	1.01%
249	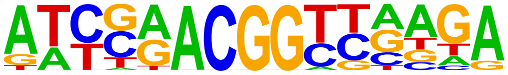	ZIM(C2C2gata)/col-ZIM-DAP-Seq(GSE60143)/Homer	1e-4	-1.080e+01	0.0001	105.0	0.16%
250	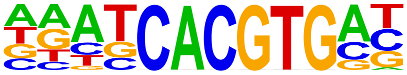	bHLH74(bHLH)/col-bHLH74-DAP-Seq(GSE60143)/Homer	1e-4	-1.075e+01	0.0001	4271.0	6.52%
251	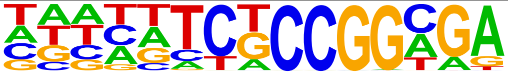	AT5G05550(Trihelix)/col-AT5G05550-DAP-Seq(GSE60143)/Homer	1e-4	-1.048e+01	0.0001	18080.0	27.58%
252	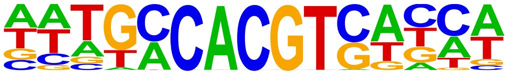	GBF6(bZIP)/colamp-GBF6-DAP-Seq(GSE60143)/Homer	1e-4	-1.044e+01	0.0001	2917.0	4.45%
253	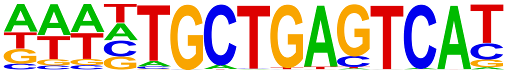	Bach1(bZIP)/K562-Bach1-ChIP-Seq(GSE31477)/Homer	1e-4	-1.042e+01	0.0001	754.0	1.15%
254	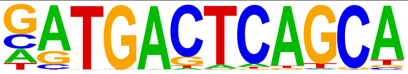	NF-E2(bZIP)/K562-NFE2-ChIP-Seq(GSE31477)/Homer	1e-4	-1.035e+01	0.0001	773.0	1.18%
255	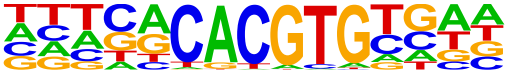	BAM8(BES1)/col-BAM8-DAP-Seq(GSE60143)/Homer	1e-4	-1.028e+01	0.0001	2343.0	3.57%
256	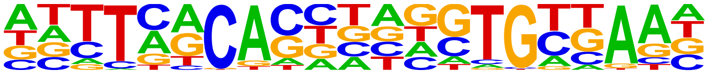	Brachyury(T-box)/Mesoendoderm-Brachyury-ChIP-exo(GSE54963)/Homer	1e-4	-1.015e+01	0.0002	4496.0	6.86%
257	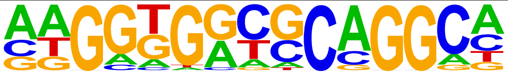	ZNF165(Zf)/WHIM12-ZNF165-ChIP-Seq(GSE65937)/Homer	1e-4	-9.888e+00	0.0002	1990.0	3.04%
258	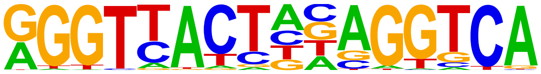	LXRE(NR),DR4/RAW-LXRb.biotin-ChIP-Seq(GSE21512)/Homer	1e-4	-9.849e+00	0.0002	563.0	0.86%
259	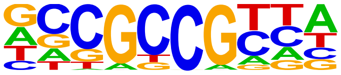	CRF10(AP2EREBP)/col100-CRF10-DAP-Seq(GSE60143)/Homer	1e-4	-9.641e+00	0.0003	15923.0	24.29%
260	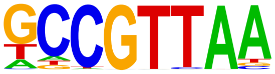	AT3G10030(Trihelix)/colamp-AT3G10030-DAP-Seq(GSE60143)/Homer	1e-4	-9.419e+00	0.0003	9312.0	14.20%
261	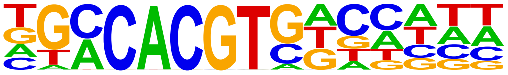	ABF2(bZIP)/col-ABF2-DAP-Seq(GSE60143)/Homer	1e-4	-9.410e+00	0.0003	2991.0	4.56%
262	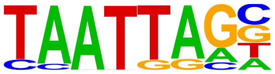	Lhx2(Homeobox)/HFSC-Lhx2-ChIP-Seq(GSE48068)/Homer	1e-4	-9.354e+00	0.0003	15009.0	22.89%
263	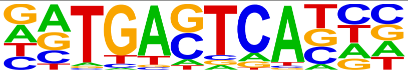	Fosl2(bZIP)/3T3L1-Fosl2-ChIP-Seq(GSE56872)/Homer	1e-3	-8.392e+00	0.0009	3564.0	5.44%
264	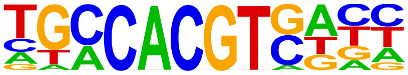	bZIP28(bZIP)/col-bZIP28-DAP-Seq(GSE60143)/Homer	1e-3	-8.342e+00	0.0009	3730.0	5.69%
265	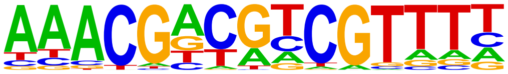	Unknown2/Arabidopsis-Promoters/Homer	1e-3	-7.319e+00	0.0025	99.0	0.15%
266	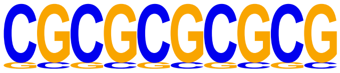	SeqBias: CG-repeat	1e-3	-7.234e+00	0.0027	17509.0	26.71%
267	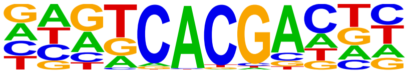	Npas4(bHLH)/Neuron-Npas4-ChIP-Seq(GSE127793)/Homer	1e-3	-7.097e+00	0.0031	13728.0	20.94%
268	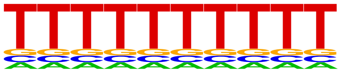	VRN1(ABI3VP1)/col-VRN1-DAP-Seq(GSE60143)/Homer	1e-3	-6.938e+00	0.0036	3793.0	5.79%

269	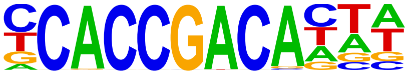	AT1G01250(AP2EREBP)/col-AT1G01250-DAP-Seq(GSE60143)/Homer	1e-2	-6.872e+00	0.0039	1174.0	1.79%
270	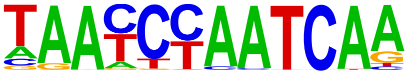	Duxbl(Homeobox)/NIH3T3-Duxbl.HA-ChIP-Seq(GSE119782)/Homer	1e-2	-6.783e+00	0.0042	702.0	1.07%
271	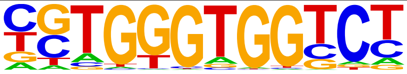	Gli2(Zf)/GM2-Gli2-ChIP-Chip(GSE112702)/Homer	1e-2	-6.683e+00	0.0046	2788.0	4.25%
272	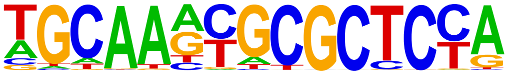	EFL-1(E2F)/cElegans-L1-EFL1-ChIP-Seq(modEncode)/Homer	1e-2	-6.567e+00	0.0052	341.0	0.52%
273	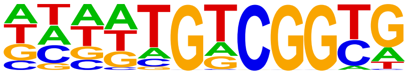	AT1G71450(AP2EREBP)/col-AT1G71450-DAP-Seq(GSE60143)/Homer	1e-2	-6.143e+00	0.0079	17262.0	26.33%
274	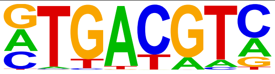	TGA9(bZIP)/colamp-TGA9-DAP-Seq(GSE60143)/Homer	1e-2	-6.065e+00	0.0085	16267.0	24.81%
275	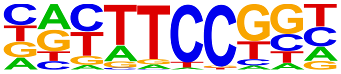	Fli1(ETS)/CD8-FLI-ChIP-Seq(GSE20898)/Homer	1e-2	-5.843e+00	0.0106	13404.0	20.45%
276	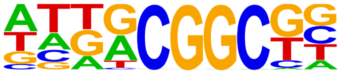	ERF115(AP2EREBP)/colamp-ERF115-DAP-Seq(GSE60143)/Homer	1e-2	-5.671e+00	0.0126	18487.0	28.20%
277	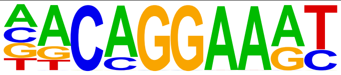	EWS:FLI1-fusion(ETS)/SK_N_MC-EWS:FLI1-ChIP-Seq(SRA014231)/Homer	1e-2	-5.632e+00	0.0130	6765.0	10.32%
278	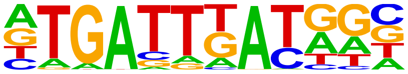	PBX2(Homeobox)/K562-PBX2-ChIP-Seq(Encode)/Homer	1e-2	-5.609e+00	0.0133	10928.0	16.67%
279	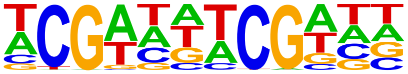	AT5G22990(C2H2)/col-AT5G22990-DAP-Seq(GSE60143)/Homer	1e-2	-5.398e+00	0.0163	956.0	1.46%
280	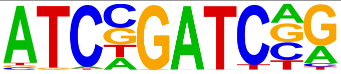	GATA19(C2C2gata)/colamp-GATA19-DAP-Seq(GSE60143)/Homer	1e-2	-5.223e+00	0.0194	974.0	1.49%
281	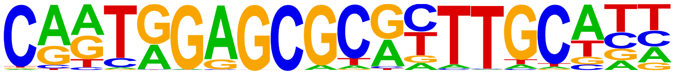	LIN-15B(Zf)/cElegans-L3-LIN15B-ChIP-Seq(modEncode)/Homer	1e-2	-5.098e+00	0.0219	89.0	0.14%
282	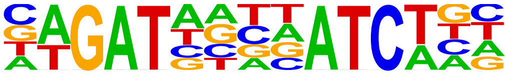	GATA(Zf),IR4/iTreg-Gata3-ChIP-Seq(GSE20898)/Homer	1e-2	-4.970e+00	0.0248	1171.0	1.79%
283	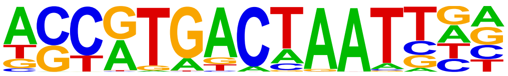	PAX3:FKHR-fusion(Paired,Homeobox)/Rh4-PAX3:FKHR-ChIP-Seq(GSE19063)/Homer	1e-2	-4.911e+00	0.0262	2794.0	4.26%
284	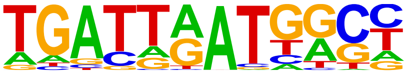	Hoxb4(Homeobox)/ES-Hoxb4-ChIP-Seq(GSE34014)/Homer	1e-2	-4.813e+00	0.0288	2616.0	3.99%
285	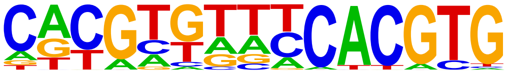	bHLH18(bHLH)/col-bHLH18-DAP-Seq(GSE60143)/Homer	1e-2	-4.644e+00	0.0340	265.0	0.40%
