# Supplementary material for: PBX1 and PBX3 transcription factors regulate SHH expression in the Frontonasal Ectodermal Zone through complementary mechanisms
Source: PLoS Genet. 2025 May 21;21(5):e1011315. doi: 10.1371/journal.pgen.1011315 (PMC12140432; doi:10.1371/journal.pgen.1011315)
Supplement: S3 Table — (PDF) [file pgen.1011315.s009.pdf]

S3 Table. Full list of known motif discovery from ChIP-seq data targeting PBX1.

Homer Known Motif Enrichment Results (/wynton/group/marcucio/2022CHM/Data2022Mar/Motif/HomerPBX1IDR)

Homer *de novo* Motif Results
Gene Ontology Enrichment Results
Known Motif Enrichment Results (txt file)
Total Target Sequences = 13432, Total Background Sequences = 36359

Rank	Motif	Name	P-value	log P-pvalue	q-value (Benjamini)	# Target Sequenc with Motif
1	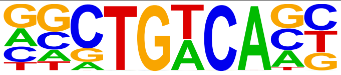	Meis1(Homeobox)/MastCells-Meis1-ChIP-Seq(GSE48085)/Homer	1e-701	-1.614e+03	0.0000	4274.0
2	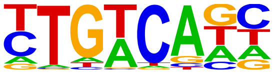	Tgif1(Homeobox)/mES-Tgif1-ChIP-Seq(GSE55404)/Homer	1e-608	-1.402e+03	0.0000	5327.0
3	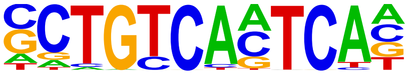	Pbx3(Homeobox)/GM12878-PBX3-ChIP-Seq(GSE32465)/Homer	1e-555	-1.279e+03	0.0000	1184.0
4	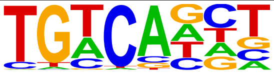	Tgif2(Homeobox)/mES-Tgif2-ChIP-Seq(GSE55404)/Homer	1e-487	-1.122e+03	0.0000	5396.0
5	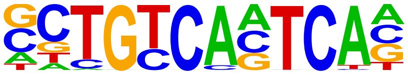	Pknox1(Homeobox)/ES-Prep1-ChIP-Seq(GSE63282)/Homer	1e-404	-9.305e+02	0.0000	1120.0
6	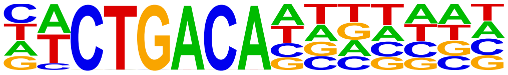	GRF9(GRF)/colamp-GRF9-DAP-Seq(GSE60143)/Homer	1e-395	-9.103e+02	0.0000	2189.0
7	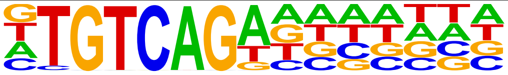	AtGRF6(GRF)/col-AtGRF6-DAP-Seq(GSE60143)/Homer	1e-356	-8.200e+02	0.0000	2646.0
8	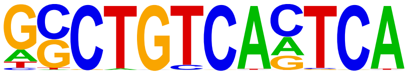	PBX1(Homeobox)/MCF7-PBX1-ChIP-Seq(GSE28007)/Homer	1e-311	-7.181e+02	0.0000	506.0
9	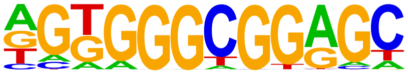	Sp5(Zf)/mES-Sp5.Flag-ChIP-Seq(GSE72989)/Homer	1e-123	-2.833e+02	0.0000	4245.0
10	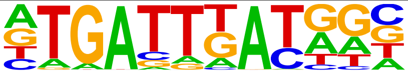	PBX2(Homeobox)/K562-PBX2-ChIP-Seq(Encode)/Homer	1e-119	-2.749e+02	0.0000	1108.0
11	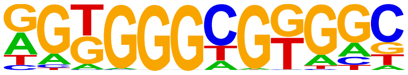	KLF14(Zf)/HEK293-KLF14.GFP-ChIP-Seq(GSE58341)/Homer	1e-115	-2.666e+02	0.0000	6303.0
12	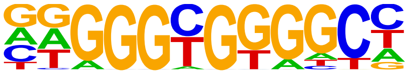	KLF1(Zf)/HUDEP2-KLF1-CutnRun(GSE136251)/Homer	1e-115	-2.662e+02	0.0000	3253.0
13	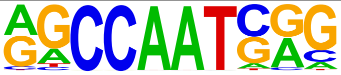	NFY(CCAAT)/Promoter/Homer	1e-108	-2.487e+02	0.0000	1512.0
14	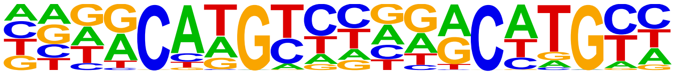	p63(p53)/Keratinocyte-p63-ChIP-Seq(GSE17611)/Homer	1e-106	-2.443e+02	0.0000	653.0
15	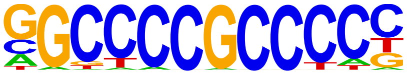	Sp1(Zf)/Promoter/Homer	1e-95	-2.209e+02	0.0000	2341.0
16	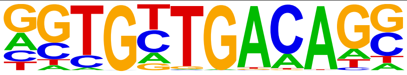	Tbx20(T-box)/Heart-Tbx20-ChIP-Seq(GSE29636)/Homer	1e-94	-2.168e+02	0.0000	738.0
17	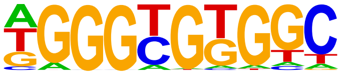	KLF5(Zf)/LoVo-KLF5-ChIP-Seq(GSE49402)/Homer	1e-93	-2.142e+02	0.0000	4106.0
18	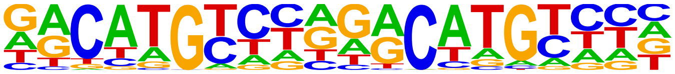	p53(p53)/Saos-p53-ChIP-Seq(GSE15780)/Homer	1e-92	-2.124e+02	0.0000	261.0
19	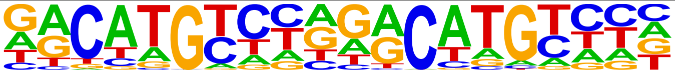	p53(p53)/Saos-p53-ChIP-Seq/Homer	1e-92	-2.124e+02	0.0000	261.0
20	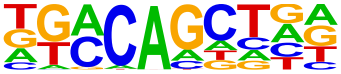	bZIP18(bZIP)/colamp-bZIP18-DAP-Seq(GSE60143)/Homer	1e-91	-2.097e+02	0.0000	7253.0

21	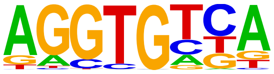	Tbx5(T-box)/HL1-Tbx5.biotin-ChIP-Seq(GSE21529)/Homer	1e-87	-2.025e+02	0.0000	4208.0
22	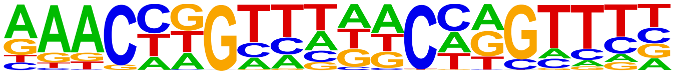	GRHL2(CP2)/HBE-GRHL2-ChIP-Seq(GSE46194)/Homer	1e-87	-2.022e+02	0.0000	520.0
23	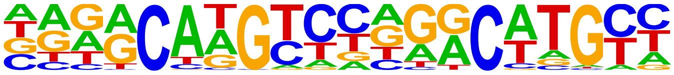	p73(p53)/Trachea-p73-ChIP-Seq(PRJNA310161)/Homer	1e-85	-1.978e+02	0.0000	170.0
24	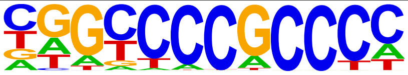	Sp2(Zf)/HEK293-Sp2.eGFP-ChIP-Seq(Encode)/Homer	1e-85	-1.975e+02	0.0000	5298.0
25	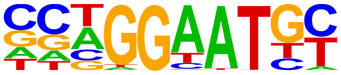	TEAD4(TEA)/Tropoblast-Tead4-ChIP-Seq(GSE37350)/Homer	1e-85	-1.973e+02	0.0000	1080.0
26	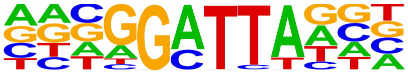	bcd(Homeobox)/Embryo-Bcd-ChIP-Seq(GSE86966)/Homer	1e-84	-1.940e+02	0.0000	1262.0
27	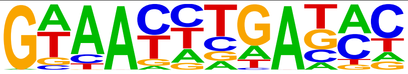	Six2(Homeobox)/NephronProgenitor-Six2-ChIP-Seq(GSE39837)/Homer	1e-82	-1.889e+02	0.0000	1102.0
28	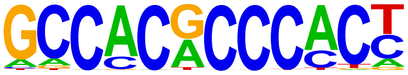	Klf9(Zf)/GBM-Klf9-ChIP-Seq(GSE62211)/Homer	1e-81	-1.883e+02	0.0000	1362.0
29	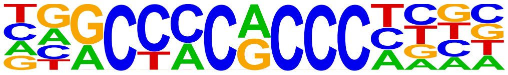	KLF3(Zf)/MEF-Klf3-ChIP-Seq(GSE44748)/Homer	1e-81	-1.865e+02	0.0000	1901.0
30	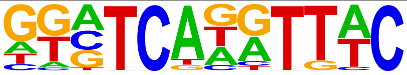	Six1(Homeobox)/Myoblast-Six1-ChIP-Chip(GSE20150)/Homer	1e-80	-1.845e+02	0.0000	388.0
31	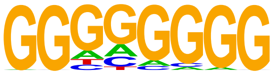	Maz(Zf)/HepG2-Maz-ChIP-Seq(GSE31477)/Homer	1e-78	-1.817e+02	0.0000	5912.0
32	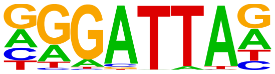	GSC(Homeobox)/FrogEmbryos-GSC-ChIP-Seq(DRA000576)/Homer	1e-74	-1.710e+02	0.0000	1189.0
33	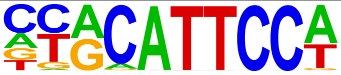	TEAD1(TEAD)/HepG2-TEAD1-ChIP-Seq(Encode)/Homer	1e-73	-1.691e+02	0.0000	1106.0
34	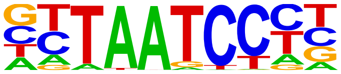	Otx2(Homeobox)/EpiLC-Otx2-ChIP-Seq(GSE56098)/Homer	1e-73	-1.690e+02	0.0000	890.0
35	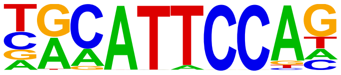	TEAD3(TEA)/HepG2-TEAD3-ChIP-Seq(Encode)/Homer	1e-72	-1.677e+02	0.0000	1226.0
36	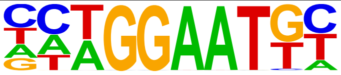	TEAD(TEA)/Fibroblast-PU.1-ChIP-Seq(Unpublished)/Homer	1e-68	-1.584e+02	0.0000	774.0
37	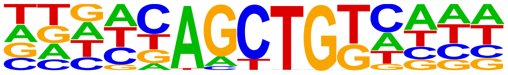	bZIP52(bZIP)/colamp-bZIP52-DAP-Seq(GSE60143)/Homer	1e-65	-1.518e+02	0.0000	1884.0
38	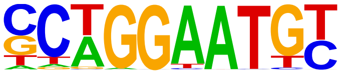	TEAD2(TEA)/Py2T-Tead2-ChIP-Seq(GSE55709)/Homer	1e-64	-1.494e+02	0.0000	684.0
39	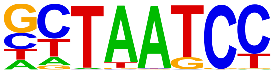	CRX(Homeobox)/Retina-Crx-ChIP-Seq(GSE20012)/Homer	1e-58	-1.356e+02	0.0000	2170.0
40	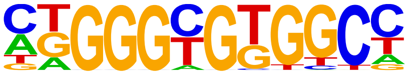	KLF6(Zf)/PDAC-KLF6-ChIP-Seq(GSE64557)/Homer	1e-57	-1.327e+02	0.0000	3653.0
41	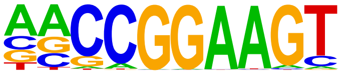	ETS(ETS)/Promoter/Homer	1e-53	-1.237e+02	0.0000	801.0
42	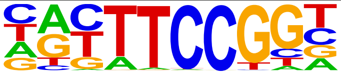	Elk4(ETS)/Hela-Elk4-ChIP-Seq(GSE31477)/Homer	1e-53	-1.235e+02	0.0000	1381.0
43	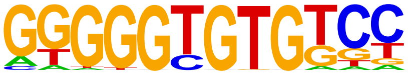	KLF10(Zf)/HEK293-KLF10.GFP-ChIP-Seq(GSE58341)/Homer	1e-52	-1.209e+02	0.0000	1075.0
44	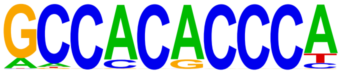	Klf4(Zf)/mES-Klf4-ChIP-Seq(GSE11431)/Homer	1e-51	-1.188e+02	0.0000	783.0
45	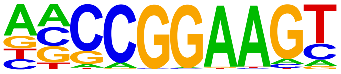	ELF1(ETS)/Jurkat-ELF1-ChIP-Seq(SRA014231)/Homer	1e-49	-1.132e+02	0.0000	1196.0

46	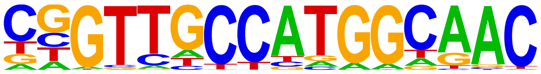	RFX(HTH)/K562-RFX3-ChIP-Seq(SRA012198)/Homer	1e-49	-1.129e+02	0.0000	341.0
47	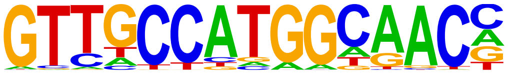	Rfx2(HTH)/LoVo-RFX2-ChIP-Seq(GSE49402)/Homer	1e-48	-1.120e+02	0.0000	346.0
48	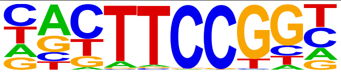	Elk1(ETS)/Hela-Elk1-ChIP-Seq(GSE31477)/Homer	1e-45	-1.054e+02	0.0000	1399.0
49	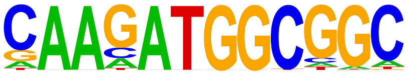	YY1(Zf)/Promoter/Homer	1e-44	-1.021e+02	0.0000	525.0
50	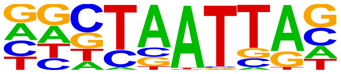	En1(Homeobox)/SUM149-EN1-ChIP-Seq(GSE120957)/Homer	1e-43	-1.003e+02	0.0000	1858.0
51	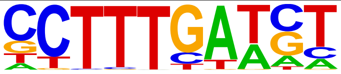	LEF1(HMG)/H1-LEF1-ChIP-Seq(GSE64758)/Homer	1e-42	-9.707e+01	0.0000	774.0
52	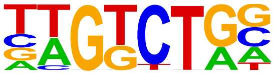	Smad3(MAD)/NPC-Smad3-ChIP-Seq(GSE36673)/Homer	1e-40	-9.394e+01	0.0000	2779.0
53	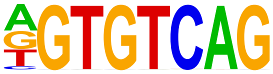	CELF2(RRM)/JSL1-CELF2-CLIP-Seq(GSE71264)/Homer	1e-40	-9.290e+01	0.0000	559.0
54	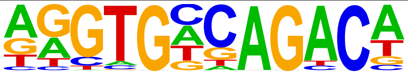	Tbox:Smad(T-box,MAD)/ESCd5-Smad2_3-ChIP-Seq(GSE29422)/Homer	1e-39	-8.981e+01	0.0000	276.0
55	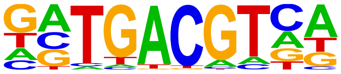	bZIP50(bZIP)/colamp-bZIP50-DAP-Seq(GSE60143)/Homer	1e-38	-8.798e+01	0.0000	1495.0
56	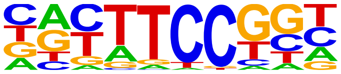	Fli1(ETS)/CD8-FLI-ChIP-Seq(GSE20898)/Homer	1e-37	-8.620e+01	0.0000	1980.0
57	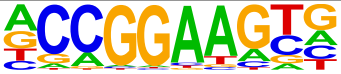	ETV4(ETS)/HepG2-ETV4-ChIP-Seq(ENCODE)/Homer	1e-37	-8.610e+01	0.0000	2156.0
58	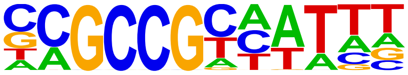	RRTF1(AP2EREBP)/colamp-RRTF1-DAP-Seq(GSE60143)/Homer	1e-37	-8.596e+01	0.0000	1317.0
59	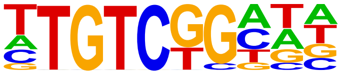	ARF2(ARF)/col-ARF2-DAP-Seq(GSE60143)/Homer	1e-37	-8.583e+01	0.0000	3585.0
60	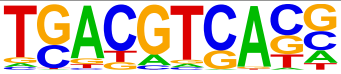	FEA4(bZIP)/Corn-FEA4-ChIP-Seq(GSE61954)/Homer	1e-37	-8.538e+01	0.0000	1759.0
61	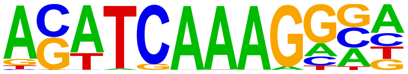	Tcf4(HMG)/Hct116-Tcf4-ChIP-Seq(SRA012054)/Homer	1e-36	-8.445e+01	0.0000	539.0
62	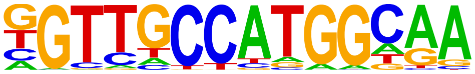	Rfx1(HTH)/NPC-H3K4me1-ChIP-Seq(GSE16256)/Homer	1e-36	-8.419e+01	0.0000	449.0
63	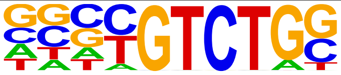	Smad4(MAD)/ESC-SMAD4-ChIP-Seq(GSE29422)/Homer	1e-36	-8.304e+01	0.0000	2074.0
64	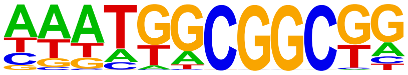	ABR1(AP2EREBP)/colamp-ABR1-DAP-Seq(GSE60143)/Homer	1e-35	-8.227e+01	0.0000	5148.0
65	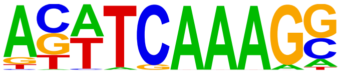	Tcf3(HMG)/mES-Tcf3-ChIP-Seq(GSE11724)/Homer	1e-34	-7.976e+01	0.0000	331.0
66	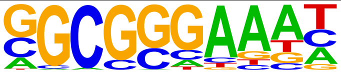	E2F4(E2F)/K562-E2F4-ChIP-Seq(GSE31477)/Homer	1e-34	-7.944e+01	0.0000	1991.0
67	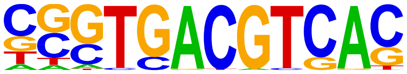	CRE(bZIP)/Promoter/Homer	1e-34	-7.941e+01	0.0000	763.0
68	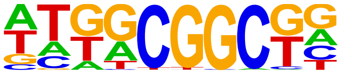	At2g33710(AP2EREBP)/colamp-At2g33710-DAP-Seq(GSE60143)/Homer	1e-34	-7.852e+01	0.0000	7310.0
69	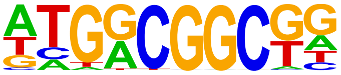	ERF7(AP2EREBP)/col-ERF7-DAP-Seq(GSE60143)/Homer	1e-32	-7.447e+01	0.0000	6148.0
70	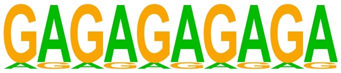	SeqBias: GA-repeat	1e-32	-7.379e+01	0.0000	8836.0

71	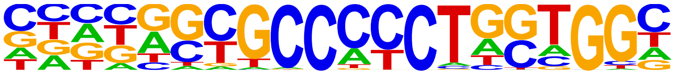	BORIS(Zf)/K562-CTCFL-ChIP-Seq(GSE32465)/Homer	1e-32	-7.375e+01	0.0000	1084.0
72	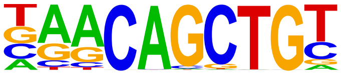	Myf5(bHLH)/GM-Myf5-ChIP-Seq(GSE24852)/Homer	1e-31	-7.300e+01	0.0000	1373.0
73	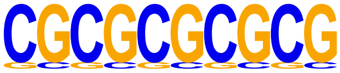	SeqBias: CG-repeat	1e-31	-7.287e+01	0.0000	8741.0
74	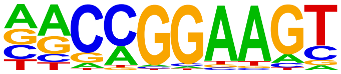	GABPA(ETS)/Jurkat-GABPa-ChIP-Seq(GSE17954)/Homer	1e-31	-7.185e+01	0.0000	1416.0
75	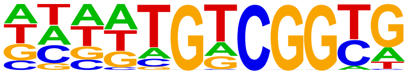	AT1G71450(AP2EREBP)/col-AT1G71450-DAP-Seq(GSE60143)/Homer	1e-31	-7.156e+01	0.0000	5605.0
76	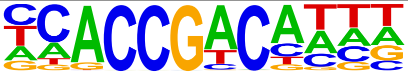	DEAR2(AP2EREBP)/colamp-DEAR2-DAP-Seq(GSE60143)/Homer	1e-30	-7.120e+01	0.0000	2788.0
77	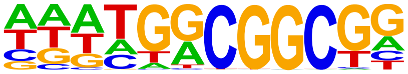	ERF4(AP2EREBP)/colamp-ERF4-DAP-Seq(GSE60143)/Homer	1e-30	-7.102e+01	0.0000	5732.0
78	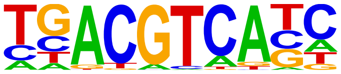	TGA6(bZIP)/colamp-TGA6-DAP-Seq(GSE60143)/Homer	1e-30	-7.017e+01	0.0000	983.0
79	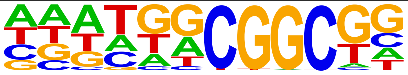	RAP26(AP2EREBP)/colamp-RAP26-DAP-Seq(GSE60143)/Homer	1e-30	-7.010e+01	0.0000	6295.0
80	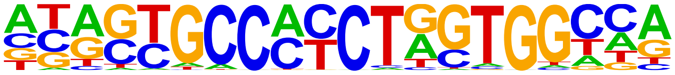	CTCF(Zf)/CD4+-CTCF-ChIP-Seq(Barski_et_al.)/Homer	1e-30	-6.995e+01	0.0000	491.0
81	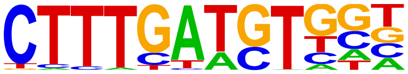	Tcf7(HMG)/GM12878-TCF7-ChIP-Seq(Encode)/Homer	1e-30	-6.957e+01	0.0000	417.0
82	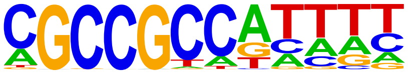	RAP212(AP2EREBP)/col-RAP212-DAP-Seq(GSE60143)/Homer	1e-29	-6.876e+01	0.0000	4669.0
83	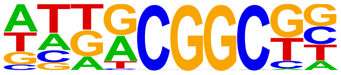	ERF115(AP2EREBP)/colamp-ERF115-DAP-Seq(GSE60143)/Homer	1e-29	-6.800e+01	0.0000	7330.0
84	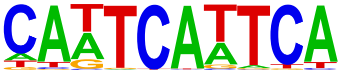	WUS1(Homeobox)/colamp-WUS1-DAP-Seq(GSE60143)/Homer	1e-29	-6.731e+01	0.0000	434.0
85	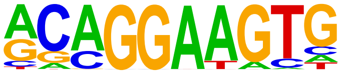	ETS1(ETS)/Jurkat-ETS1-ChIP-Seq(GSE17954)/Homer	1e-28	-6.640e+01	0.0000	1611.0
86	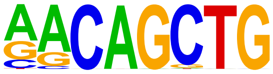	MyoG(bHLH)/C2C12-MyoG-ChIP-Seq(GSE36024)/Homer	1e-28	-6.589e+01	0.0000	1963.0
87	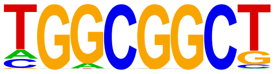	ERF105(AP2EREBP)/colamp-ERF105-DAP-Seq(GSE60143)/Homer	1e-28	-6.492e+01	0.0000	7098.0
88	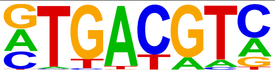	TGA9(bZIP)/colamp-TGA9-DAP-Seq(GSE60143)/Homer	1e-28	-6.485e+01	0.0000	1533.0
89	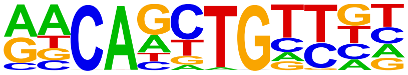	HLH-1(bHLH)/cElegans-Embryo-HLH1-ChIP-Seq(modEncode)/Homer	1e-28	-6.469e+01	0.0000	1023.0
90	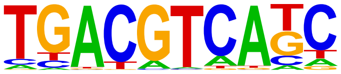	TGA1(bZIP)/colamp-TGA1-DAP-Seq(GSE60143)/Homer	1e-27	-6.410e+01	0.0000	677.0
91	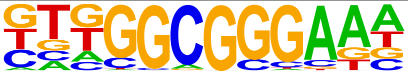	E2F3(E2F)/MEF-E2F3-ChIP-Seq(GSE71376)/Homer	1e-27	-6.391e+01	0.0000	2825.0
92	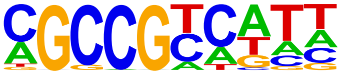	ERF8(AP2EREBP)/colamp-ERF8-DAP-Seq(GSE60143)/Homer	1e-27	-6.366e+01	0.0000	5619.0
93	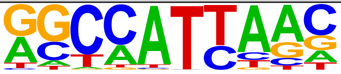	Nanog(Homeobox)/mES-Nanog-ChIP-Seq(GSE11724)/Homer	1e-27	-6.350e+01	0.0000	4240.0
94	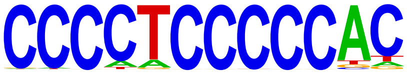	Zfp281(Zf)/ES-Zfp281-ChIP-Seq(GSE81042)/Homer	1e-27	-6.300e+01	0.0000	688.0
95	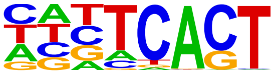	STZ(C2H2)/colamp-STZ-DAP-Seq(GSE60143)/Homer	1e-26	-6.145e+01	0.0000	5336.0

96	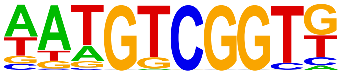	DREB19(AP2EREBP)/colamp-DREB19-DAP-Seq(GSE60143)/Homer	1e-26	-6.115e+01	0.0000	1480.0
97	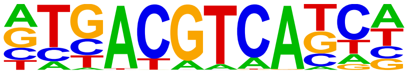	TGA4(bZIP)/colamp-TGA4-DAP-Seq(GSE60143)/Homer	1e-26	-6.045e+01	0.0000	534.0
98	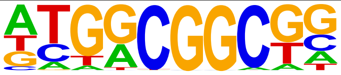	ERF3(AP2EREBP)/colamp-ERF3-DAP-Seq(GSE60143)/Homer	1e-26	-6.012e+01	0.0000	4988.0
99	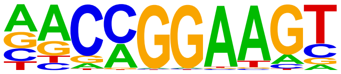	ETV1(ETS)/GIST48-ETV1-ChIP-Seq(GSE22441)/Homer	1e-26	-6.006e+01	0.0000	1922.0
100	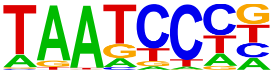	Pitx1(Homeobox)/Chicken-Pitx1-ChIP-Seq(GSE38910)/Homer	1e-26	-5.990e+01	0.0000	3718.0
101	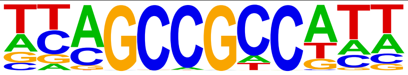	ERF13(AP2EREBP)/colamp-ERF13-DAP-Seq(GSE60143)/Homer	1e-25	-5.945e+01	0.0000	5520.0
102	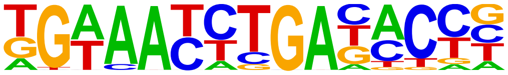	Six4(Homeobox)/MCF7-SIX4-ChIP-Seq(Encode)/Homer	1e-25	-5.803e+01	0.0000	105.0
103	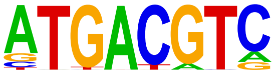	TGA10(bZIP)/colamp-TGA10-DAP-Seq(GSE60143)/Homer	1e-24	-5.694e+01	0.0000	878.0
104	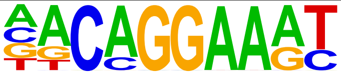	EWS:FLI1-fusion(ETS)/SK_N_MC-EWS:FLI1-ChIP-Seq(SRA014231)/Homer	1e-24	-5.595e+01	0.0000	875.0
105	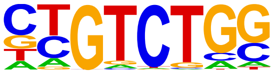	Smad2(MAD)/ES-SMAD2-ChIP-Seq(GSE29422)/Homer	1e-24	-5.578e+01	0.0000	2023.0
106	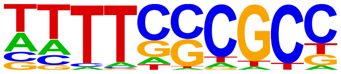	DEL2(E2FDP)/col-DEL2-DAP-Seq(GSE60143)/Homer	1e-24	-5.570e+01	0.0000	947.0
107	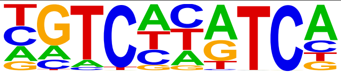	Replumless(BLH)/Arabidopsis-RPL.GFP-ChIP-Seq(GSE78727)/Homer	1e-23	-5.366e+01	0.0000	1220.0
108	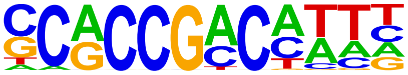	At5g65130(AP2EREBP)/colamp-At5g65130-DAP-Seq(GSE60143)/Homer	1e-23	-5.312e+01	0.0000	1392.0
109	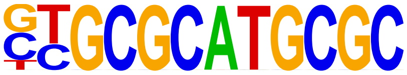	NRF(NRF)/Promoter/Homer	1e-23	-5.305e+01	0.0000	777.0
110	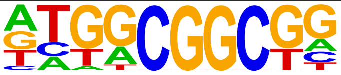	ERF11(AP2EREBP)/col-ERF11-DAP-Seq(GSE60143)/Homer	1e-23	-5.298e+01	0.0000	5499.0
111	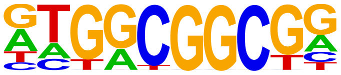	ERF10(AP2EREBP)/col-ERF10-DAP-Seq(GSE60143)/Homer	1e-22	-5.295e+01	0.0000	4957.0
112	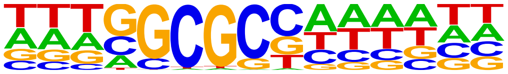	E2FA(E2FDP)/colamp-E2FA-DAP-Seq(GSE60143)/Homer	1e-22	-5.269e+01	0.0000	757.0
113	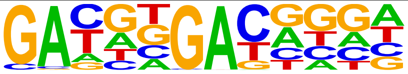	Knotted(Homeobox)/Corn-KN1-ChIP-Seq(GSE39161)/Homer	1e-22	-5.249e+01	0.0000	2474.0
114	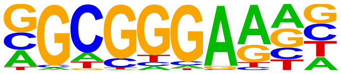	E2F6(E2F)/Hela-E2F6-ChIP-Seq(GSE31477)/Homer	1e-22	-5.127e+01	0.0000	2621.0
115	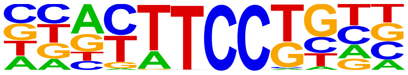	Etv2(ETS)/ES-ER71-ChIP-Seq(GSE59402)/Homer	1e-22	-5.124e+01	0.0000	1233.0
116	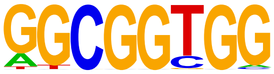	AT3G57600(AP2EREBP)/col-AT3G57600-DAP-Seq(GSE60143)/Homer	1e-22	-5.121e+01	0.0000	5864.0
117	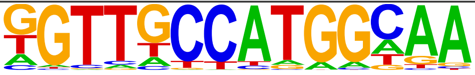	X-box(HTH)/NPC-H3K4me1-ChIP-Seq(GSE16256)/Homer	1e-21	-5.055e+01	0.0000	219.0
118	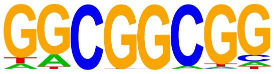	AT1G28160(AP2EREBP)/colamp-AT1G28160-DAP-Seq(GSE60143)/Homer	1e-21	-5.031e+01	0.0000	8242.0
119	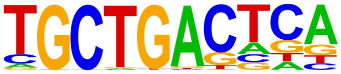	MafA(bZIP)/Islet-MafA-ChIP-Seq(GSE30298)/Homer	1e-21	-4.994e+01	0.0000	1344.0
120		E2F7(E2F)/Hela-E2F7-ChIP-Seq(GSE32673)/Homer	1e-21	-4.978e+01	0.0000	535.0

	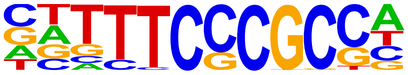					
121	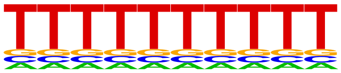	VRN1(ABI3VP1)/col-VRN1-DAP-Seq(GSE60143)/Homer	1e-20	-4.793e+01	0.0000	299.0
122	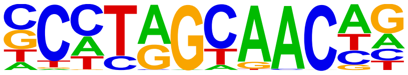	Rfx5(HTH)/GM12878-Rfx5-ChIP-Seq(GSE31477)/Homer	1e-20	-4.740e+01	0.0000	450.0
123	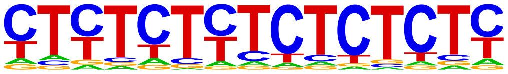	GAGA-repeat/SacCer-Promoters/Homer	1e-20	-4.717e+01	0.0000	4209.0
124	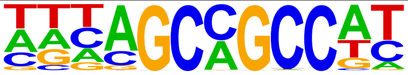	ERF15(AP2EREBP)/colamp-ERF15-DAP-Seq(GSE60143)/Homer	1e-20	-4.688e+01	0.0000	7653.0
125	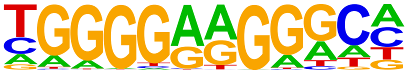	ZNF467(Zf)/HEK293-ZNF467.GFP-ChIP-Seq(GSE58341)/Homer	1e-20	-4.681e+01	0.0000	2628.0
126	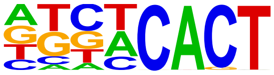	AZF1(C2H2)/colamp-AZF1-DAP-Seq(GSE60143)/Homer	1e-20	-4.670e+01	0.0000	4879.0
127	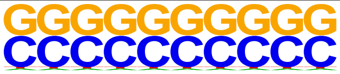	SeqBias: CG bias	1e-20	-4.664e+01	0.0000	11976.0
128	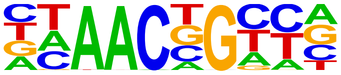	BMYB(HTH)/Hela-BMYB-ChIP-Seq(GSE27030)/Homer	1e-19	-4.522e+01	0.0000	2102.0
129	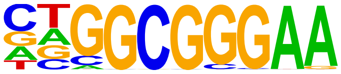	E2F1(E2F)/Hela-E2F1-ChIP-Seq(GSE22478)/Homer	1e-19	-4.468e+01	0.0000	1279.0
130	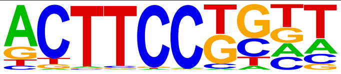	Elf4(ETS)/BMDM-Elf4-ChIP-Seq(GSE88699)/Homer	1e-18	-4.336e+01	0.0000	1236.0
131	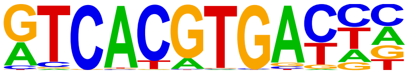	TFE3(bHLH)/MEF-TFE3-ChIP-Seq(GSE75757)/Homer	1e-18	-4.233e+01	0.0000	189.0
132	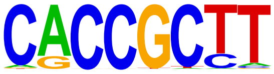	At5g18450(AP2EREBP)/col-At5g18450-DAP-Seq(GSE60143)/Homer	1e-18	-4.204e+01	0.0000	6977.0
133	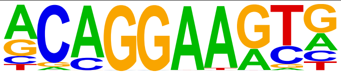	ERG(ETS)/VCaP-ERG-ChIP-Seq(GSE14097)/Homer	1e-18	-4.184e+01	0.0000	1965.0
134	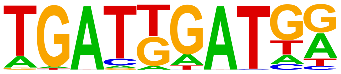	HOXA1(Homeobox)/mES-Hoxa1-ChIP-Seq(SRP084292)/Homer	1e-17	-4.126e+01	0.0000	303.0
135	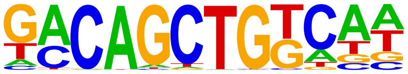	bZIP69(bZIP)/col-bZIP69-DAP-Seq(GSE60143)/Homer	1e-17	-4.056e+01	0.0000	215.0
136	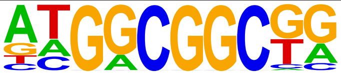	AT4G18450(AP2EREBP)/col-AT4G18450-DAP-Seq(GSE60143)/Homer	1e-17	-4.033e+01	0.0000	4016.0
137	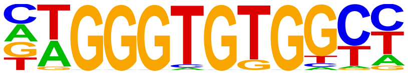	EKLF(Zf)/Erythrocyte-Klf1-ChIP-Seq(GSE20478)/Homer	1e-17	-3.997e+01	0.0000	182.0
138	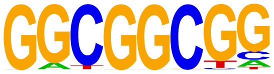	ESE1(AP2EREBP)/col-ESE1-DAP-Seq(GSE60143)/Homer	1e-17	-3.984e+01	0.0000	6283.0
139	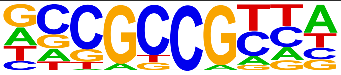	CRF10(AP2EREBP)/col100-CRF10-DAP-Seq(GSE60143)/Homer	1e-17	-3.958e+01	0.0000	7424.0
140	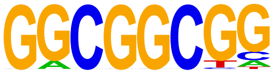	ERF104(AP2EREBP)/col-ERF104-DAP-Seq(GSE60143)/Homer	1e-17	-3.953e+01	0.0000	6848.0
141	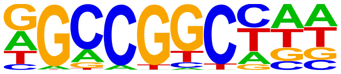	RAP211(AP2EREBP)/colamp-RAP211-DAP-Seq(GSE60143)/Homer	1e-17	-3.950e+01	0.0000	6697.0
142	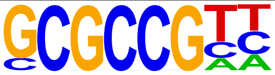	PUCHI(AP2EREBP)/colamp-PUCHI-DAP-Seq(GSE60143)/Homer	1e-17	-3.944e+01	0.0000	6249.0
143	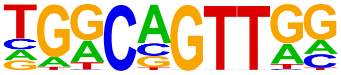	AMYB(HTH)/Testes-AMYB-ChIP-Seq(GSE44588)/Homer	1e-17	-3.940e+01	0.0000	2128.0
144	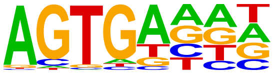	At5g04390(C2H2)/col200-At5g04390-DAP-Seq(GSE60143)/Homer	1e-17	-3.925e+01	0.0000	4922.0

145	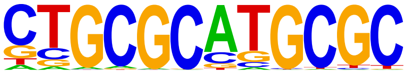	NRF1(NRF)/MCF7-NRF1-ChIP-Seq(Unpublished)/Homer	1e-16	-3.896e+01	0.0000	814.0
146	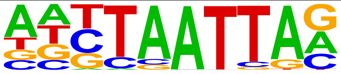	Lhx3(Homeobox)/Neuron-Lhx3-ChIP-Seq(GSE31456)/Homer	1e-16	-3.866e+01	0.0000	1406.0
147	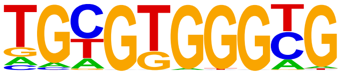	Egr1(Zf)/K562-Egr1-ChIP-Seq(GSE32465)/Homer	1e-16	-3.830e+01	0.0000	2170.0
148	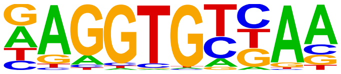	Tbx6(T-box)/ESC-Tbx6-ChIP-Seq(GSE93524)/Homer	1e-16	-3.821e+01	0.0000	1133.0
149	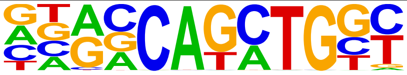	Atoh1(bHLH)/Cerebellum-Atoh1-ChIP-Seq(GSE22111)/Homer	1e-16	-3.798e+01	0.0000	1558.0
150	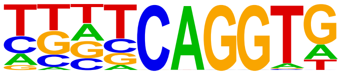	NGA4(ABI3VP1)/col-NGA4-DAP-Seq(GSE60143)/Homer	1e-16	-3.750e+01	0.0000	3258.0
151	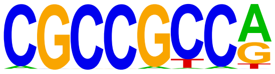	CRF4(AP2EREBP)/colamp-CRF4-DAP-Seq(GSE60143)/Homer	1e-15	-3.669e+01	0.0000	4833.0
152	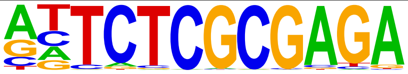	GFX(?)/Promoter/Homer	1e-15	-3.654e+01	0.0000	102.0
153	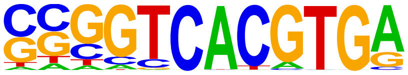	E-box(bHLH)/Promoter/Homer	1e-15	-3.638e+01	0.0000	263.0
154	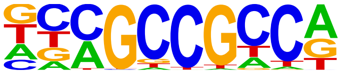	ERF5(AP2EREBP)/colamp-ERF5-DAP-Seq(GSE60143)/Homer	1e-15	-3.586e+01	0.0000	4296.0
155	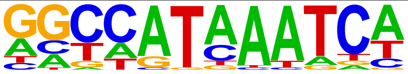	HOXA9(Homeobox)/HSC-Hoxa9-ChIP-Seq(GSE33509)/Homer	1e-15	-3.568e+01	0.0000	598.0
156	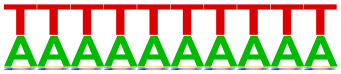	SeqBias: A/T bias	1e-15	-3.558e+01	0.0000	5914.0
157	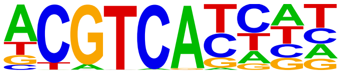	TGA2(bZIP)/colamp-TGA2-DAP-Seq(GSE60143)/Homer	1e-15	-3.554e+01	0.0000	985.0
158	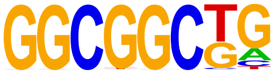	ERF2(AP2EREBP)/colamp-ERF2-DAP-Seq(GSE60143)/Homer	1e-15	-3.502e+01	0.0000	5993.0
159	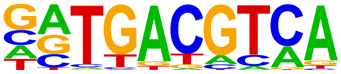	Atf1(bZIP)/K562-ATF1-ChIP-Seq(GSE31477)/Homer	1e-14	-3.449e+01	0.0000	849.0
160	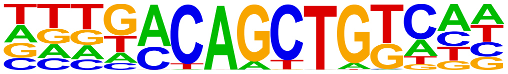	VIP1(bZIP)/col-VIP1-DAP-Seq(GSE60143)/Homer	1e-14	-3.442e+01	0.0000	286.0
161	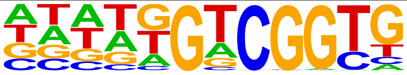	At1g36060(AP2EREBP)/colamp-At1g36060-DAP-Seq(GSE60143)/Homer	1e-14	-3.411e+01	0.0000	1600.0
162	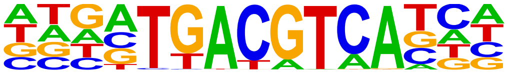	TGA5(bZIP)/col-TGA5-DAP-Seq(GSE60143)/Homer	1e-14	-3.407e+01	0.0000	253.0
163	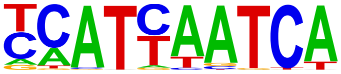	Pdx1(Homeobox)/Islet-Pdx1-ChIP-Seq(SRA008281)/Homer	1e-14	-3.390e+01	0.0000	817.0
164	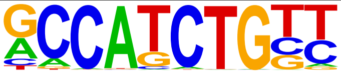	NeuroD1(bHLH)/Islet-NeuroD1-ChIP-Seq(GSE30298)/Homer	1e-14	-3.367e+01	0.0000	1007.0
165	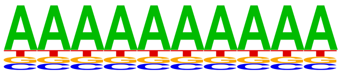	SeqBias: polyA-repeat	1e-14	-3.342e+01	0.0000	10468.0
166	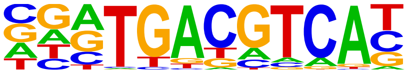	Atf7(bZIP)/3T3L1-Atf7-ChIP-Seq(GSE56872)/Homer	1e-14	-3.342e+01	0.0000	670.0
167	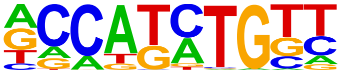	Olig2(bHLH)/Neuron-Olig2-ChIP-Seq(GSE30882)/Homer	1e-14	-3.324e+01	0.0000	2102.0
168	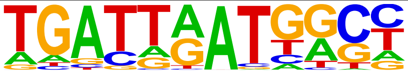	Hoxb4(Homeobox)/ES-Hoxb4-ChIP-Seq(GSE34014)/Homer	1e-14	-3.290e+01	0.0000	220.0
169	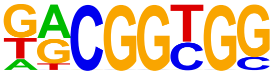	ESE3(AP2EREBP)/col-ESE3-DAP-Seq(GSE60143)/Homer	1e-14	-3.286e+01	0.0000	7119.0

170	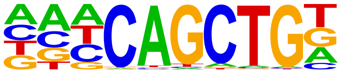	Ap4(bHLH)/AML-Tfap4-ChIP-Seq(GSE45738)/Homer	1e-14	-3.240e+01	0.0000	2047.0
171	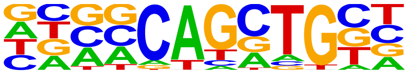	Ascl1(bHLH)/NeuralTubes-Ascl1-ChIP-Seq(GSE55840)/Homer	1e-14	-3.229e+01	0.0000	2475.0
172	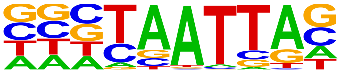	DLX2(Homeobox)/BasalGanglia-Dlx2-ChIP-seq(GSE124936)/Homer	1e-13	-3.222e+01	0.0000	1239.0
173	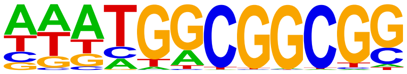	ERF9(AP2EREBP)/colamp-ERF9-DAP-Seq(GSE60143)/Homer	1e-13	-3.208e+01	0.0000	2994.0
174	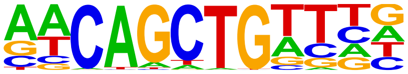	E-box/Drosophila-Promoters/Homer	1e-13	-3.177e+01	0.0000	395.0
175	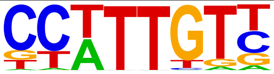	Sox3(HMG)/NPC-Sox3-ChIP-Seq(GSE33059)/Homer	1e-13	-3.174e+01	0.0000	1787.0
176	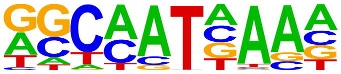	Hoxa9(Homeobox)/ChickenMSG-Hoxa9.Flag-ChIP-Seq(GSE86088)/Homer	1e-13	-3.146e+01	0.0000	2410.0
177	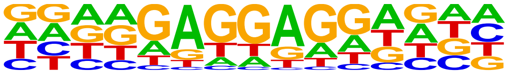	TF3A(C2H2)/col-TF3A-DAP-Seq(GSE60143)/Homer	1e-13	-3.120e+01	0.0000	2294.0
178	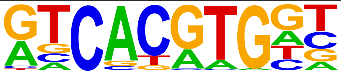	Usf2(bHLH)/C2C12-Usf2-ChIP-Seq(GSE36030)/Homer	1e-13	-3.111e+01	0.0000	469.0
179	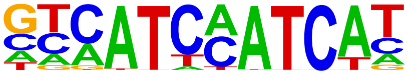	HOXA2(Homeobox)/mES-Hoxa2-ChIP-Seq(Donaldson_et_al.)/Homer	1e-13	-3.043e+01	0.0000	134.0
180	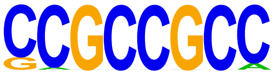	ERF73(AP2EREBP)/col-ERF73-DAP-Seq(GSE60143)/Homer	1e-13	-3.010e+01	0.0000	5840.0
181	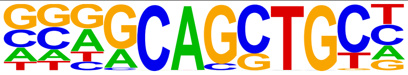	Ascl2(bHLH)/ESC-Ascl2-ChIP-Seq(GSE97712)/Homer	1e-12	-2.950e+01	0.0000	2089.0
182	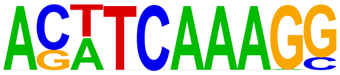	TCFL2(HMG)/K562-TCF7L2-ChIP-Seq(GSE29196)/Homer	1e-12	-2.943e+01	0.0000	124.0
183	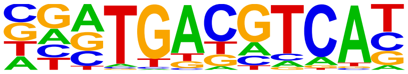	Atf2(bZIP)/3T3L1-Atf2-ChIP-Seq(GSE56872)/Homer	1e-12	-2.925e+01	0.0000	507.0
184	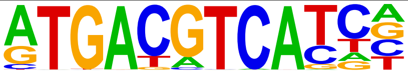	JunD(bZIP)/K562-JunD-ChIP-Seq/Homer	1e-12	-2.884e+01	0.0000	247.0
185	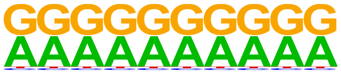	SeqBias: G/A bias	1e-12	-2.867e+01	0.0000	13429.0
186	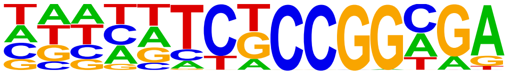	AT5G05550(Trihelix)/col-AT5G05550-DAP-Seq(GSE60143)/Homer	1e-12	-2.855e+01	0.0000	3546.0
187	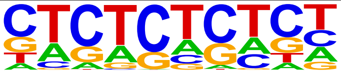	GAGA-repeat/Arabidopsis-Promoters/Homer	1e-12	-2.839e+01	0.0000	1246.0
188	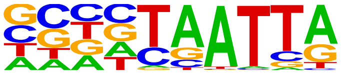	DLX1(Homeobox)/BasalGanglia-Dlx1-ChIP-seq(GSE124936)/Homer	1e-12	-2.826e+01	0.0000	1141.0
189	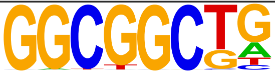	AT5G23930(mTERF)/col-AT5G23930-DAP-Seq(GSE60143)/Homer	1e-12	-2.795e+01	0.0000	7628.0
190	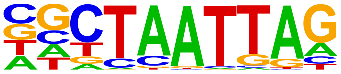	LHX9(Homeobox)/Hct116-LHX9.V5-ChIP-Seq(GSE116822)/Homer	1e-12	-2.789e+01	0.0000	1202.0
191	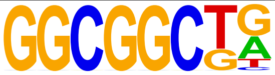	ERF1(AP2EREBP)/colamp-ERF1-DAP-Seq(GSE60143)/Homer	1e-12	-2.775e+01	0.0000	5439.0
192	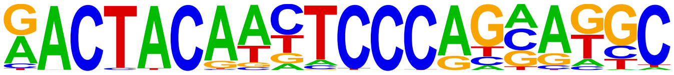	Ronin(THAP)/ES-Thap11-ChIP-Seq(GSE51522)/Homer	1e-11	-2.756e+01	0.0000	158.0
193	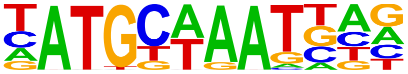	Brn1(POU,Homeobox)/NPC-Brn1-ChIP-Seq(GSE35496)/Homer	1e-11	-2.728e+01	0.0000	233.0
194	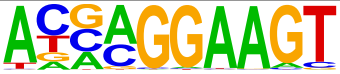	ELF5(ETS)/T47D-ELF5-ChIP-Seq(GSE30407)/Homer	1e-11	-2.713e+01	0.0000	772.0

195	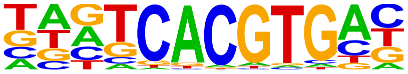	BIM3(bHLH)/col-BIM3-DAP-Seq(GSE60143)/Homer	1e-11	-2.694e+01	0.0000	247.0
196	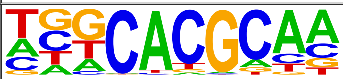	Arnt:Ahr(bHLH)/MCF7-Arnt-ChIP-Seq(Lo_et_al.)/Homer	1e-11	-2.692e+01	0.0000	1451.0
197	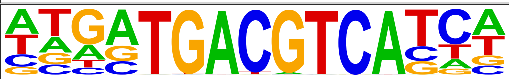	TGA3(bZIP)/colamp-TGA3-DAP-Seq(GSE60143)/Homer	1e-11	-2.679e+01	0.0000	195.0
198	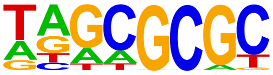	DPL-1(E2F)/cElegans-Adult-ChIP-Seq(modEncode)/Homer	1e-11	-2.637e+01	0.0000	3146.0
199	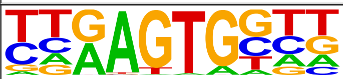	Bapx1(Homeobox)/VertebralCol-Bapx1-ChIP-Seq(GSE36672)/Homer	1e-11	-2.620e+01	0.0000	1894.0
200	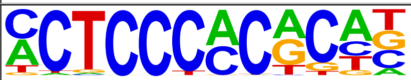	WT1(Zf)/Kidney-WT1-ChIP-Seq(GSE90016)/Homer	1e-11	-2.618e+01	0.0000	1716.0
201	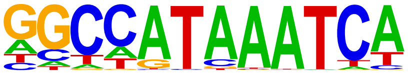	Hoxc9(Homeobox)/Ainv15-Hoxc9-ChIP-Seq(GSE21812)/Homer	1e-11	-2.602e+01	0.0000	445.0
202	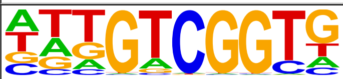	CEJ1(AP2EREBP)/col-CEJ1-DAP-Seq(GSE60143)/Homer	1e-11	-2.555e+01	0.0000	2031.0
203	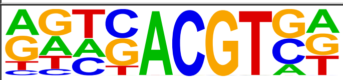	HY5(bZIP)/colamp-HY5-DAP-Seq(GSE60143)/Homer	1e-10	-2.523e+01	0.0000	1258.0
204	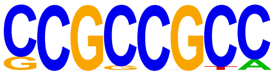	SHN3(AP2EREBP)/col-SHN3-DAP-Seq(GSE60143)/Homer	1e-10	-2.485e+01	0.0000	4558.0
205	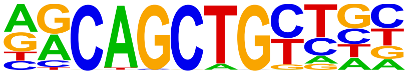	MyoD(bHLH)/Myotube-MyoD-ChIP-Seq(GSE21614)/Homer	1e-10	-2.474e+01	0.0000	1765.0
206	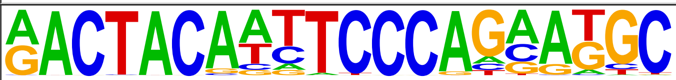	GFY-Staf(?,Zf)/Promoter/Homer	1e-10	-2.466e+01	0.0000	178.0
207	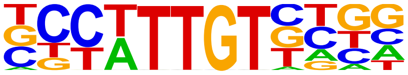	Sox21(HMG)/ESC-SOX21-ChIP-Seq(GSE110505)/Homer	1e-10	-2.458e+01	0.0000	1876.0
208	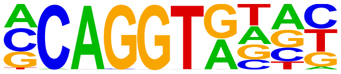	ZEB1(Zf)/PDAC-ZEB1-ChIP-Seq(GSE64557)/Homer	1e-10	-2.451e+01	0.0000	2443.0
209	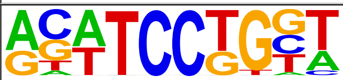	SPDEF(ETS)/VCaP-SPDEF-ChIP-Seq(SRA014231)/Homer	1e-10	-2.447e+01	0.0000	1073.0
210	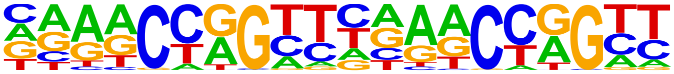	Tcfcp2l1(CP2)/mES-Tcfcp2l1-ChIP-Seq(GSE11431)/Homer	1e-10	-2.381e+01	0.0000	175.0
211	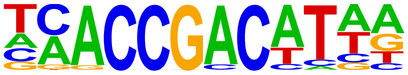	DREB2(AP2EREBP)/col-DREB2-DAP-Seq(GSE60143)/Homer	1e-10	-2.367e+01	0.0000	924.0
212	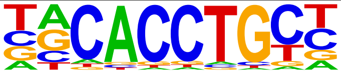	Snail1(Zf)/LS174T-SNAIL1.HA-ChIP-Seq(GSE127183)/Homer	1e-10	-2.360e+01	0.0000	1365.0
213	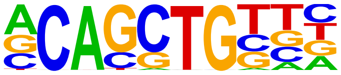	Ptf1a(bHLH)/Panc1-Ptf1a-ChIP-Seq(GSE47459)/Homer	1e-10	-2.354e+01	0.0000	3871.0
214	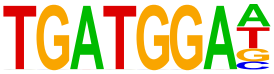	HAP3(CCAATHAP3)/col-HAP3-DAP-Seq(GSE60143)/Homer	1e-10	-2.330e+01	0.0000	394.0
215	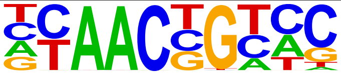	MYB77(MYB)/col-MYB77-DAP-Seq(GSE60143)/Homer	1e-10	-2.303e+01	0.0000	1752.0
216	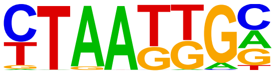	Isl1(Homeobox)/Neuron-Isl1-ChIP-Seq(GSE31456)/Homer	1e-9	-2.297e+01	0.0000	1604.0
217	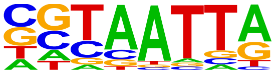	DLX5(Homeobox)/BasalGanglia-Dlx5-ChIP-seq(GSE124936)/Homer	1e-9	-2.235e+01	0.0000	697.0
218	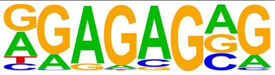	Trl(Zf)/S2-GAGAfactor-ChIP-Seq(GSE40646)/Homer	1e-9	-2.212e+01	0.0000	5249.0
219	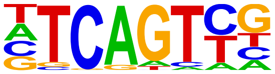	Initiator/Drosophila-Promoters/Homer	1e-9	-2.211e+01	0.0000	1972.0

220	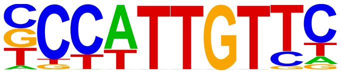	Sox2(HMG)/mES-Sox2-ChIP-Seq(GSE11431)/Homer	1e-9	-2.205e+01	0.0000	945.0
221	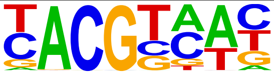	ATAF1(NAC)/col-ATAF1-DAP-Seq(GSE60143)/Homer	1e-9	-2.197e+01	0.0000	4034.0
222	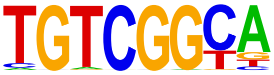	AT1G12630(AP2EREBP)/colamp-AT1G12630-DAP-Seq(GSE60143)/Homer	1e-9	-2.190e+01	0.0000	1111.0
223	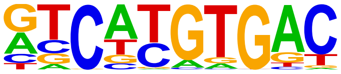	MITF(bHLH)/MastCells-MITF-ChIP-Seq(GSE48085)/Homer	1e-9	-2.180e+01	0.0000	1012.0
224	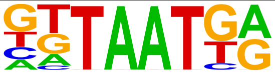	Nkx6.1(Homeobox)/Islet-Nkx6.1-ChIP-Seq(GSE40975)/Homer	1e-9	-2.106e+01	0.0000	2025.0
225	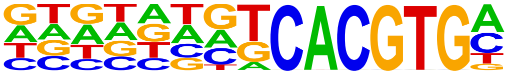	BIM1(bHLH)/colamp-BIM1-DAP-Seq(GSE60143)/Homer	1e-9	-2.079e+01	0.0000	219.0
226	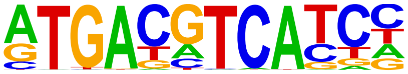	c-Jun-CRE(bZIP)/K562-cJun-ChIP-Seq(GSE31477)/Homer	1e-9	-2.076e+01	0.0000	440.0
227	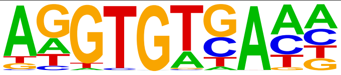	Tbet(T-box)/CD8-Tbet-ChIP-Seq(GSE33802)/Homer	1e-8	-2.072e+01	0.0000	869.0
228	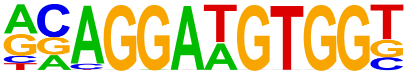	ETS:RUNX(ETS,Runt)/Jurkat-RUNX1-ChIP-Seq(GSE17954)/Homer	1e-8	-2.050e+01	0.0000	150.0
229	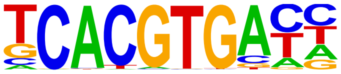	Cbf1(bHLH)/Yeast-Cbf1-ChIP-Seq(GSE29506)/Homer	1e-8	-2.016e+01	0.0000	456.0
230	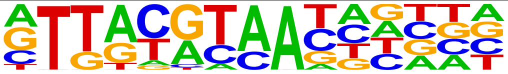	NFIL3(bZIP)/HepG2-NFIL3-ChIP-Seq(Encode)/Homer	1e-8	-2.007e+01	0.0000	620.0
231	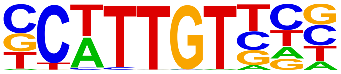	Sox10(HMG)/SciaticNerve-Sox3-ChIP-Seq(GSE35132)/Homer	1e-8	-2.000e+01	0.0000	1739.0
232	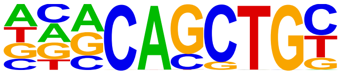	E2A(bHLH)/proBcell-E2A-ChIP-Seq(GSE21978)/Homer	1e-8	-1.989e+01	0.0000	2393.0
233	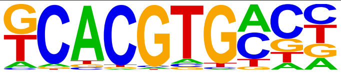	bHLHE41(bHLH)/proB-Bhlhe41-ChIP-Seq(GSE93764)/Homer	1e-8	-1.986e+01	0.0000	2380.0
234	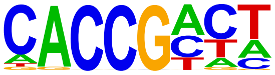	At1g75490(AP2EREBP)/colamp-At1g75490-DAP-Seq(GSE60143)/Homer	1e-8	-1.956e+01	0.0000	5183.0
235	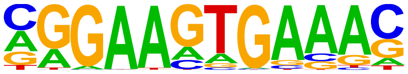	PU.1-IRF(ETS:IRF)/Bcell-PU.1-ChIP-Seq(GSE21512)/Homer	1e-8	-1.944e+01	0.0000	1342.0
236	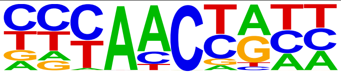	ATY13(MYB)/col-ATY13-DAP-Seq(GSE60143)/Homer	1e-8	-1.934e+01	0.0000	3166.0
237	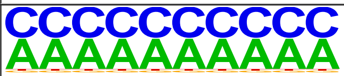	SeqBias: C/A-bias	1e-8	-1.905e+01	0.0000	13419.0
238	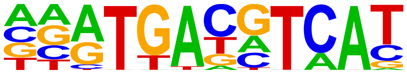	CREB5(bZIP)/LNCaP-CREB5.V5-ChIP-Seq(GSE137775)/Homer	1e-8	-1.879e+01	0.0000	448.0
239	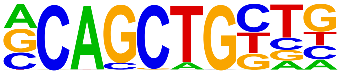	Tcf12(bHLH)/GM12878-Tcf12-ChIP-Seq(GSE32465)/Homer	1e-8	-1.875e+01	0.0000	1774.0
240	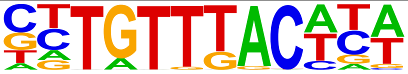	Foxa2(Forkhead)/Liver-Foxa2-ChIP-Seq(GSE25694)/Homer	1e-8	-1.872e+01	0.0000	623.0
241	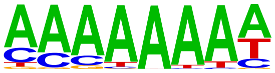	REM19(REM)/colamp-REM19-DAP-Seq(GSE60143)/Homer	1e-7	-1.837e+01	0.0000	629.0
242	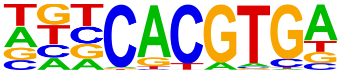	SPCH(bHLH)/Seedling-SPCH-ChIP-Seq(GSE57497)/Homer	1e-7	-1.831e+01	0.0000	1518.0
243	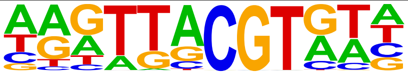	NAP(NAC)/col-NAP-DAP-Seq(GSE60143)/Homer	1e-7	-1.816e+01	0.0000	962.0
244		At3g60580(C2H2)/col-At3g60580-DAP-Seq(GSE60143)/Homer	1e-7	-1.732e+01	0.0000	4075.0

	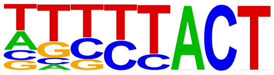					
245	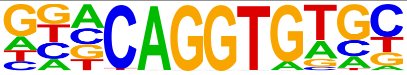	ZEB2(Zf)/SNU398-ZEB2-ChIP-Seq(GSE103048)/Homer	1e-7	-1.714e+01	0.0000	1360.0
246	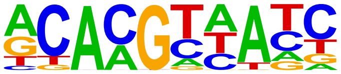	ANAC038(NAC)/col-ANAC038-DAP-Seq(GSE60143)/Homer	1e-7	-1.709e+01	0.0000	1878.0
247	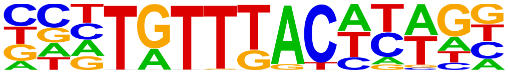	Foxa3(Forkhead)/Liver-Foxa3-ChIP-Seq(GSE77670)/Homer	1e-7	-1.705e+01	0.0000	247.0
248	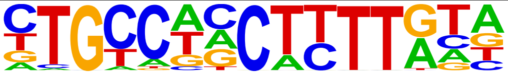	ZNF7(Zf)/HepG2-ZNF7.Flag-ChIP-Seq(Encode)/Homer	1e-7	-1.692e+01	0.0000	457.0
249	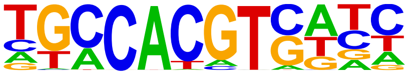	GBF3(bZIP)/Arabidopsis-GBF3-ChIP-Seq(GSE80564)/Homer	1e-7	-1.691e+01	0.0000	480.0
250	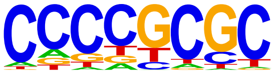	SUT1?/SacCer-Promoters/Homer	1e-7	-1.682e+01	0.0000	11234.0
251	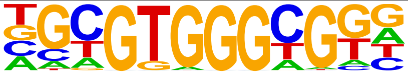	Egr2(Zf)/Thymocytes-Egr2-ChIP-Seq(GSE34254)/Homer	1e-7	-1.652e+01	0.0000	744.0
252	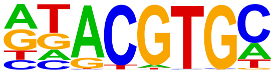	HIF-1b(HLH)/T47D-HIF1b-ChIP-Seq(GSE59937)/Homer	1e-7	-1.646e+01	0.0000	2313.0
253	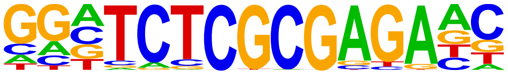	ZBTB33(Zf)/GM12878-ZBTB33-ChIP-Seq(GSE32465)/Homer	1e-7	-1.640e+01	0.0000	215.0
254	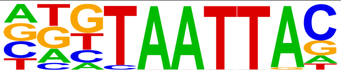	Dlx3(Homeobox)/Kerainocytes-Dlx3-ChIP-Seq(GSE89884)/Homer	1e-7	-1.628e+01	0.0000	582.0
255	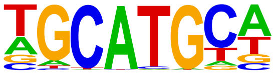	RBFox2(?)/Heart-RBFox2-CLIP-Seq(GSE57926)/Homer	1e-7	-1.624e+01	0.0000	2181.0
256	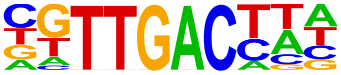	WRKY28(WRKY)/col-WRKY28-DAP-Seq(GSE60143)/Homer	1e-7	-1.622e+01	0.0000	715.0
257	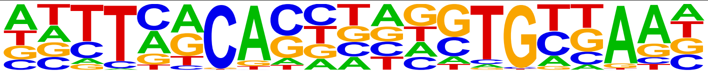	Brachyury(T-box)/Mesoendoderm-Brachyury-ChIP-exo(GSE54963)/Homer	1e-6	-1.609e+01	0.0000	269.0
258	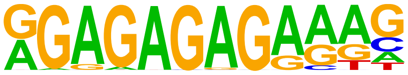	FRS9(ND)/col-FRS9-DAP-Seq(GSE60143)/Homer	1e-6	-1.573e+01	0.0000	354.0
259	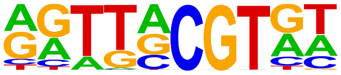	NAM(NAC)/col-NAM-DAP-Seq(GSE60143)/Homer	1e-6	-1.571e+01	0.0000	1598.0
260	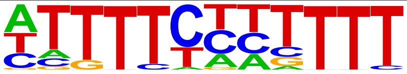	RLR1?/SacCer-Promoters/Homer	1e-6	-1.548e+01	0.0000	516.0
261	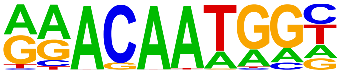	Sox15(HMG)/CPA-Sox15-ChIP-Seq(GSE62909)/Homer	1e-6	-1.504e+01	0.0000	951.0
262	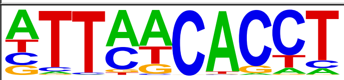	Eomes(T-box)/H9-Eomes-ChIP-Seq(GSE26097)/Homer	1e-6	-1.497e+01	0.0000	1748.0
263	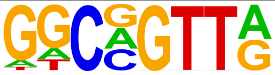	MYB(HTH)/ERMYB-Myb-ChIPSeq(GSE22095)/Homer	1e-6	-1.470e+01	0.0000	2619.0
264	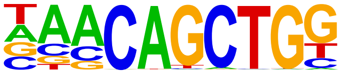	Tcf21(bHLH)/ArterySmoothMuscle-Tcf21-ChIP-Seq(GSE61369)/Homer	1e-6	-1.450e+01	0.0000	1499.0
265	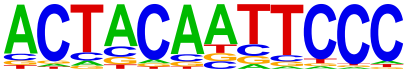	GFY(?)/Promoter/Homer	1e-6	-1.436e+01	0.0000	154.0
266	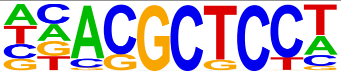	MYB88(MYB)/col-MYB88-DAP-Seq(GSE60143)/Homer	1e-6	-1.412e+01	0.0000	6533.0
267	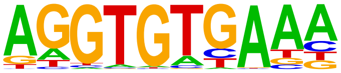	Tbx21(T-box)/GM12878-TBX21-ChIP-Seq(Encode)/Homer	1e-6	-1.410e+01	0.0000	792.0
268	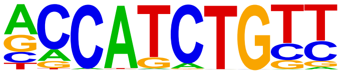	NeuroG2(bHLH)/Fibroblast-NeuroG2-ChIP-Seq(GSE75910)/Homer	1e-6	-1.407e+01	0.0000	1771.0

269	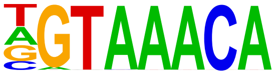	Foxo3(Forkhead)/U2OS-Foxo3-ChIP-Seq(E-MTAB-2701)/Homer	1e-6	-1.390e+01	0.0000	554.0
270	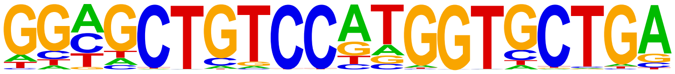	REST-NRSF(Zf)/Jurkat-NRSF-ChIP-Seq/Homer	1e-5	-1.364e+01	0.0000	47.0
271	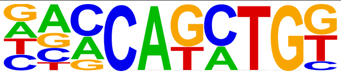	BHLHA15(bHLH)/NIH3T3-BHLHB8.HA-ChIP-Seq(GSE119782)/Homer	1e-5	-1.362e+01	0.0000	1730.0
272	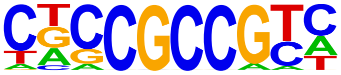	LEP(AP2EREBP)/col-LEP-DAP-Seq(GSE60143)/Homer	1e-5	-1.338e+01	0.0000	3849.0
273	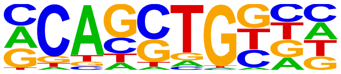	HEB(bHLH)/mES-Heb-ChIP-Seq(GSE53233)/Homer	1e-5	-1.312e+01	0.0000	2902.0
274	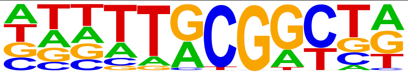	At5g08750(C3H)/col-At5g08750-DAP-Seq(GSE60143)/Homer	1e-5	-1.299e+01	0.0000	845.0
275	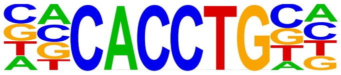	E2A(bHLH),near_PU.1/Bcell-PU.1-ChIP-Seq(GSE21512)/Homer	1e-5	-1.296e+01	0.0000	1994.0
276	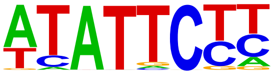	KAN2(G2like)/colamp-KAN2-DAP-Seq(GSE60143)/Homer	1e-5	-1.246e+01	0.0000	882.0
277	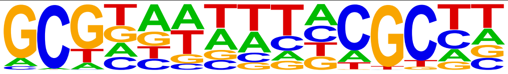	LBD23(LOBAS2)/colamp-LBD23-DAP-Seq(GSE60143)/Homer	1e-5	-1.193e+01	0.0000	2887.0
278	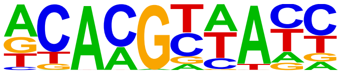	ANAC046(NAC)/colamp-ANAC046-DAP-Seq(GSE60143)/Homer	1e-5	-1.188e+01	0.0000	1680.0
279	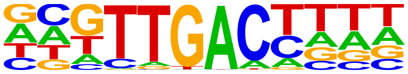	WRKY27(WRKY)/colamp-WRKY27-DAP-Seq(GSE60143)/Homer	1e-5	-1.169e+01	0.0000	475.0
280	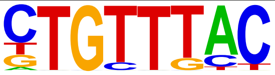	Foxo1(Forkhead)/RAW-Foxo1-ChIP-Seq(Fan_et_al.)/Homer	1e-5	-1.167e+01	0.0000	1669.0
281	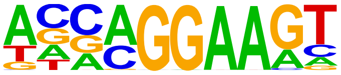	EHF(ETS)/LoVo-EHF-ChIP-Seq(GSE49402)/Homer	1e-5	-1.160e+01	0.0000	1287.0
282	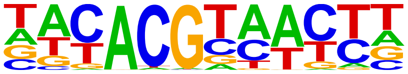	ANAC047(NAC)/colamp-ANAC047-DAP-Seq(GSE60143)/Homer	1e-4	-1.150e+01	0.0000	683.0
283	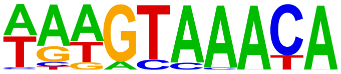	FOXA1(Forkhead)/LNCAP-FOXA1-ChIP-Seq(GSE27824)/Homer	1e-4	-1.134e+01	0.0000	853.0
284	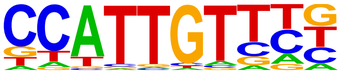	Sox17(HMG)/Endoderm-Sox17-ChIP-Seq(GSE61475)/Homer	1e-4	-1.111e+01	0.0001	616.0
285	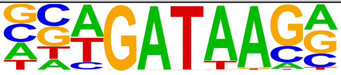	Gata4(Zf)/Heart-Gata4-ChIP-Seq(GSE35151)/Homer	1e-4	-1.102e+01	0.0001	703.0
286	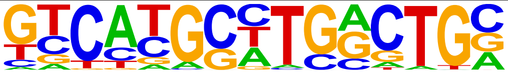	Pax8(Paired,Homeobox)/Thyroid-Pax8-ChIP-Seq(GSE26938)/Homer	1e-4	-1.091e+01	0.0001	420.0
287	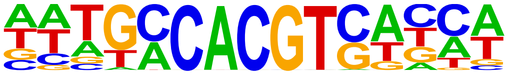	GBF6(bZIP)/colamp-GBF6-DAP-Seq(GSE60143)/Homer	1e-4	-1.083e+01	0.0001	255.0
288	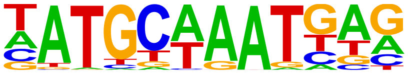	Oct6(POU,Homeobox)/NPC-Pou3f1-ChIP-Seq(GSE35496)/Homer	1e-4	-1.076e+01	0.0001	280.0
289	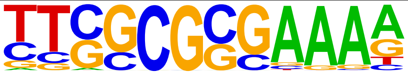	E2F(E2F)/Hela-CellCycle-Expression/Homer	1e-4	-1.073e+01	0.0001	154.0
290	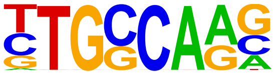	NF1-halfsite(CTF)/LNCaP-NF1-ChIP-Seq(Unpublished)/Homer	1e-4	-1.067e+01	0.0001	2003.0
291	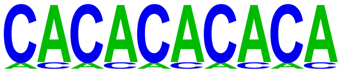	SeqBias: CA-repeat	1e-4	-1.051e+01	0.0001	5835.0
292	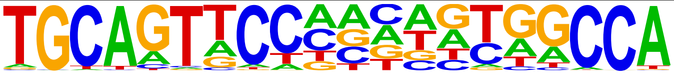	CTCF-SatelliteElement(Zf?)/CD4+-CTCF-ChIP-Seq(Barski_et_al.)/Homer	1e-4	-1.023e+01	0.0001	24.0
293	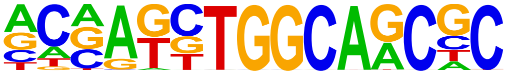	Unknown3/Drosophila-Promoters/Homer	1e-4	-9.783e+00	0.0002	215.0

294	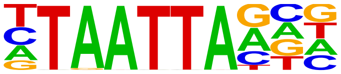	ATHB23(ZFHD)/col-ATHB23-DAP-Seq(GSE60143)/Homer	1e-4	-9.693e+00	0.0002	632.0
295	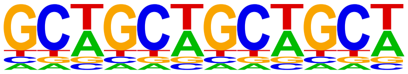	SeqBias: GCW-triplet	1e-4	-9.607e+00	0.0002	13430.0
296	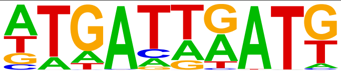	LIN-39(Homeobox)/cElegans.L3-LIN39-ChIP-Seq(modEncode)/Homer	1e-4	-9.579e+00	0.0002	827.0
297	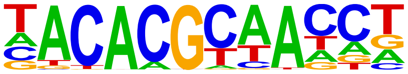	ANAC079(NAC)/colamp-ANAC079-DAP-Seq(GSE60143)/Homer	1e-4	-9.567e+00	0.0002	321.0
298	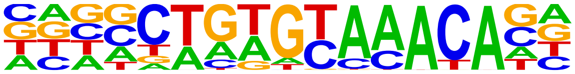	Fox:Ebox(Forkhead,bHLH)/Panc1-Foxa2-ChIP-Seq(GSE47459)/Homer	1e-4	-9.547e+00	0.0002	789.0
299	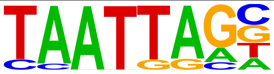	Lhx2(Homeobox)/HFSC-Lhx2-ChIP-Seq(GSE48068)/Homer	1e-4	-9.496e+00	0.0003	817.0
300	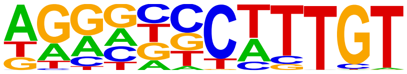	Sox9(HMG)/Limb-SOX9-ChIP-Seq(GSE73225)/Homer	1e-4	-9.451e+00	0.0003	730.0
301	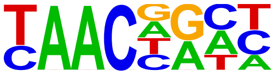	MYB101(MYB)/colamp-MYB101-DAP-Seq(GSE60143)/Homer	1e-3	-9.208e+00	0.0003	2087.0
302	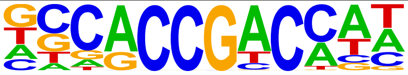	At4g28140(AP2EREBP)/colamp-At4g28140-DAP-Seq(GSE60143)/Homer	1e-3	-9.130e+00	0.0004	898.0
303	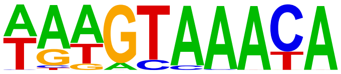	FOXA1(Forkhead)/MCF7-FOXA1-ChIP-Seq(GSE26831)/Homer	1e-3	-9.021e+00	0.0004	683.0
304	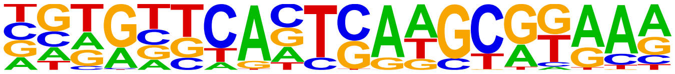	PAX6(Paired,Homeobox)/Forebrain-Pax6-ChIP-Seq(GSE66961)/Homer	1e-3	-9.017e+00	0.0004	97.0
305	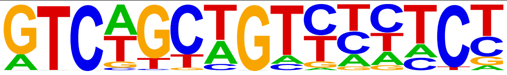	ZNF317(Zf)/HEK293-ZNF317.GFP-ChIP-Seq(GSE58341)/Homer	1e-3	-8.928e+00	0.0004	104.0
306	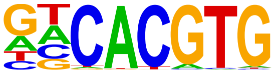	CLOCK(bHLH)/Liver-Clock-ChIP-Seq(GSE39860)/Homer	1e-3	-8.896e+00	0.0005	807.0
307	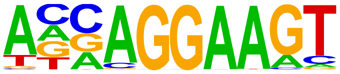	ELF3(ETS)/PDAC-ELF3-ChIP-Seq(GSE64557)/Homer	1e-3	-8.799e+00	0.0005	627.0
308	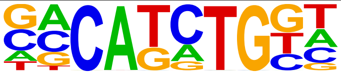	TCF4(bHLH)/SHSY5Y-TCF4-ChIP-Seq(GSE96915)/Homer	1e-3	-8.655e+00	0.0006	1710.0
309	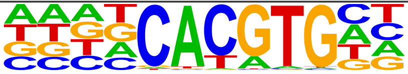	BIM2(bHLH)/col-BIM2-DAP-Seq(GSE60143)/Homer	1e-3	-8.401e+00	0.0007	990.0
310	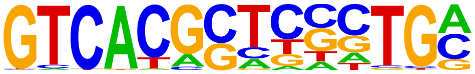	PAX5(Paired,Homeobox),condensed/GM12878-PAX5-ChIP-Seq(GSE32465)/Homer	1e-3	-8.368e+00	0.0008	175.0
311	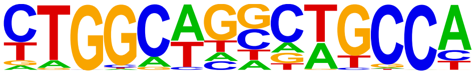	Tlx?(NR)/NPC-H3K4me1-ChIP-Seq(GSE16256)/Homer	1e-3	-8.344e+00	0.0008	398.0
312	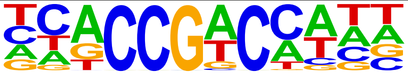	At1g19210(AP2EREBP)/colamp-At1g19210-DAP-Seq(GSE60143)/Homer	1e-3	-8.343e+00	0.0008	2691.0
313	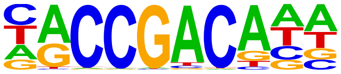	At4g16750(AP2EREBP)/col-At4g16750-DAP-Seq(GSE60143)/Homer	1e-3	-8.103e+00	0.0010	1342.0
314	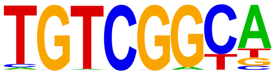	Rap210(AP2EREBP)/col-Rap210-DAP-Seq(GSE60143)/Homer	1e-3	-8.090e+00	0.0010	1207.0
315	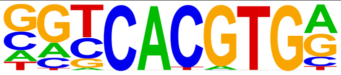	USF1(bHLH)/GM12878-Usf1-ChIP-Seq(GSE32465)/Homer	1e-3	-8.074e+00	0.0010	711.0
316	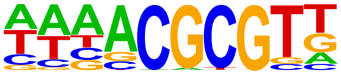	CAMTA1(CAMTA)/col-CAMTA1-DAP-Seq(GSE60143)/Homer	1e-3	-8.061e+00	0.0010	1275.0
317	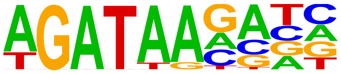	TRPS1(Zf)/MCF7-TRPS1-ChIP-Seq(GSE107013)/Homer	1e-3	-7.945e+00	0.0011	1267.0
318	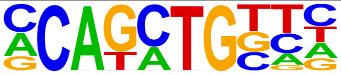	Twist2(bHLH)/Myoblast-Twist2.Ty1-ChIP-Seq(GSE127998)/Homer	1e-3	-7.904e+00	0.0012	2159.0

319	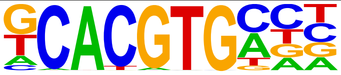	bHLHE40(bHLH)/HepG2-BHLHE40-ChIP-Seq(GSE31477)/Homer	1e-3	-7.806e+00	0.0013	580.0
320	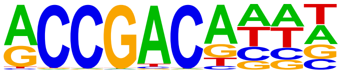	AT1G77200(AP2EREBP)/colamp-AT1G77200-DAP-Seq(GSE60143)/Homer	1e-3	-7.713e+00	0.0014	1356.0
321	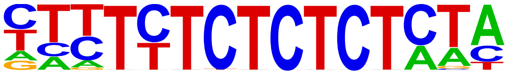	BPC6(BBRBPC)/col-BPC6-DAP-Seq(GSE60143)/Homer	1e-3	-7.578e+00	0.0016	47.0
322	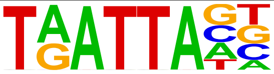	ATHB25(ZFHD)/colamp-ATHB25-DAP-Seq(GSE60143)/Homer	1e-3	-7.556e+00	0.0016	759.0
323	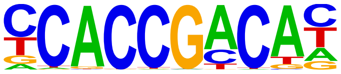	DREB26(AP2EREBP)/col-DREB26-DAP-Seq(GSE60143)/Homer	1e-3	-7.556e+00	0.0016	759.0
324	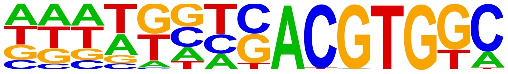	bZIP48(bZIP)/colamp-bZIP48-DAP-Seq(GSE60143)/Homer	1e-3	-7.533e+00	0.0017	362.0
325	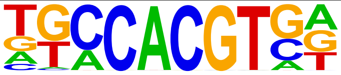	bZIP16(bZIP)/colamp-bZIP16-DAP-Seq(GSE60143)/Homer	1e-3	-7.468e+00	0.0018	391.0
326	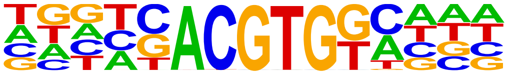	bZIP53(bZIP)/colamp-bZIP53-DAP-Seq(GSE60143)/Homer	1e-3	-7.462e+00	0.0018	411.0
327	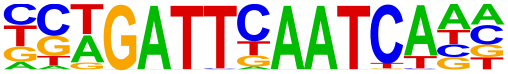	DUX(Homeobox)/C2C12-Dux-ChIP-Seq(GSE87279)/Homer	1e-3	-7.456e+00	0.0018	4.0
328	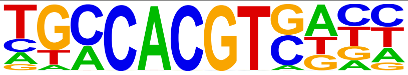	bZIP28(bZIP)/col-bZIP28-DAP-Seq(GSE60143)/Homer	1e-3	-7.330e+00	0.0020	373.0
329	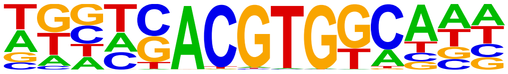	GBF5(bZIP)/colamp-GBF5-DAP-Seq(GSE60143)/Homer	1e-3	-7.289e+00	0.0021	341.0
330	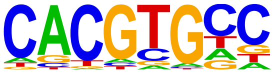	IBL1(bHLH)/Seedling-IBL1-ChIP-Seq(GSE51120)/Homer	1e-3	-7.254e+00	0.0022	4060.0
331	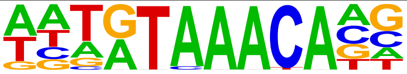	FoxL2(Forkhead)/Ovary-FoxL2-ChIP-Seq(GSE60858)/Homer	1e-3	-7.204e+00	0.0023	554.0
332	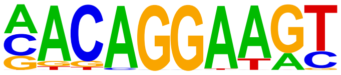	Ets1-distal(ETS)/CD4+-PolII-ChIP-Seq(Barski_et_al.)/Homer	1e-3	-7.153e+00	0.0024	226.0
333	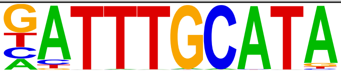	Oct11(POU,Homeobox)/NCIH1048-POU2F3-ChIP-seq(GSE115123)/Homer	1e-3	-7.043e+00	0.0026	214.0
334	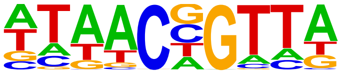	MYB70(MYB)/col-MYB70-DAP-Seq(GSE60143)/Homer	1e-3	-6.926e+00	0.0030	1137.0
335	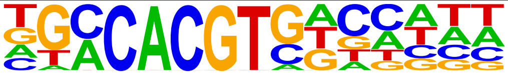	ABF2(bZIP)/col-ABF2-DAP-Seq(GSE60143)/Homer	1e-2	-6.905e+00	0.0030	291.0
336	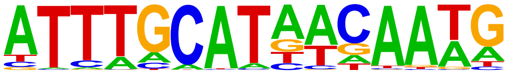	OCT4-SOX2-TCF-NANOG(POU,Homeobox,HMG)/mES-Oct4-ChIP-Seq(GSE11431)/Homer	1e-2	-6.898e+00	0.0030	110.0
337	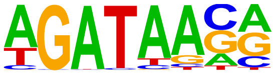	GATA3(Zf)/iTreg-Gata3-ChIP-Seq(GSE20898)/Homer	1e-2	-6.765e+00	0.0034	950.0
338	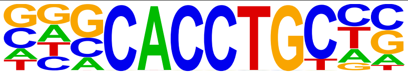	Slug(Zf)/Mesoderm-Snai2-ChIP-Seq(GSE61475)/Homer	1e-2	-6.661e+00	0.0038	869.0
339	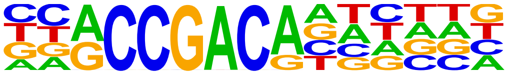	AT3G60490(AP2EREBP)/colamp-AT3G60490-DAP-Seq(GSE60143)/Homer	1e-2	-6.619e+00	0.0040	587.0
340	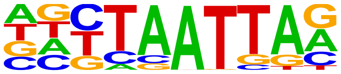	Lhx1(Homeobox)/EmbryoCarcinoma-Lhx1-ChIP-Seq(GSE70957)/Homer	1e-2	-6.607e+00	0.0040	848.0
341	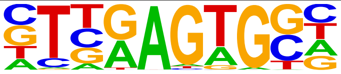	Nkx2.2(Homeobox)/NPC-Nkx2.2-ChIP-Seq(GSE61673)/Homer	1e-2	-6.574e+00	0.0041	1712.0
342	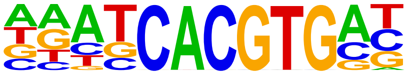	bHLH74(bHLH)/col-bHLH74-DAP-Seq(GSE60143)/Homer	1e-2	-6.564e+00	0.0041	493.0
343		bZIP44(bZIP)/colamp-bZIP44-DAP-Seq(GSE60143)/Homer	1e-2	-6.554e+00	0.0042	34.0

	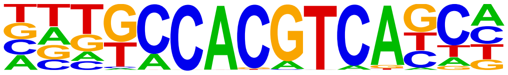					
344	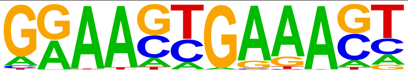	IRF8(IRF)/BMDM-IRF8-ChIP-Seq(GSE77884)/Homer	1e-2	-6.542e+00	0.0042	288.0
345	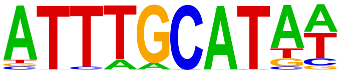	Oct4(POU,Homeobox)/mES-Oct4-ChIP-Seq(GSE11431)/Homer	1e-2	-6.476e+00	0.0045	305.0
346	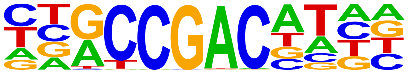	CBF3(AP2EREBP)/colamp-CBF3-DAP-Seq(GSE60143)/Homer	1e-2	-6.419e+00	0.0047	777.0
347	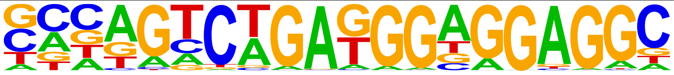	ZSCAN22(Zf)/HEK293-ZSCAN22.GFP-ChIP-Seq(GSE58341)/Homer	1e-2	-6.372e+00	0.0050	139.0
348	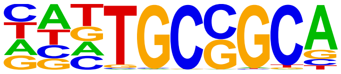	Zfp57(Zf)/H1-ZFP57.HA-ChIP-Seq(GSE115387)/Homer	1e-2	-6.325e+00	0.0052	2135.0
349	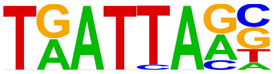	ATHB34(ZFHD)/colamp-ATHB34-DAP-Seq(GSE60143)/Homer	1e-2	-6.116e+00	0.0064	545.0
350	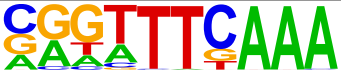	CHR(?)/Hela-CellCycle-Expression/Homer	1e-2	-6.082e+00	0.0066	418.0
351	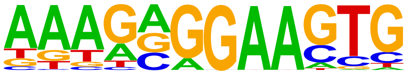	SpiB(ETS)/OCILY3-SPIB-ChIP-Seq(GSE56857)/Homer	1e-2	-6.077e+00	0.0066	227.0
352	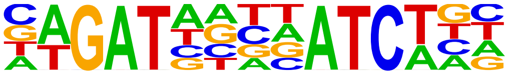	GATA(Zf),IR4/iTreg-Gata3-ChIP-Seq(GSE20898)/Homer	1e-2	-6.072e+00	0.0066	67.0
353	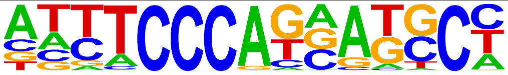	ZNF143|STAF(Zf)/CUTLL-ZNF143-ChIP-Seq(GSE29600)/Homer	1e-2	-5.982e+00	0.0072	322.0
354	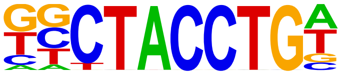	Zelda(Zf)/Embryo-zld-ChIP-Seq(GSE65441)/Homer	1e-2	-5.965e+00	0.0073	540.0
355	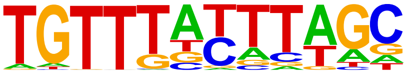	FoxD3(forkhead)/ZebrafishEmbryo-Foxd3.biotin-ChIP-seq(GSE106676)/Homer	1e-2	-5.958e+00	0.0073	622.0
356	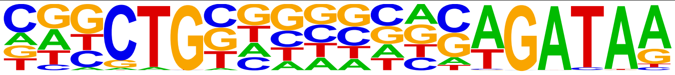	GATA:SCL(Zf,bHLH)/Ter119-SCL-ChIP-Seq(GSE18720)/Homer	1e-2	-5.872e+00	0.0080	106.0
357	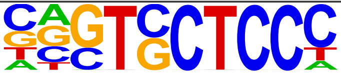	Znf263(Zf)/K562-Znf263-ChIP-Seq(GSE31477)/Homer	1e-2	-5.836e+00	0.0082	3646.0
358	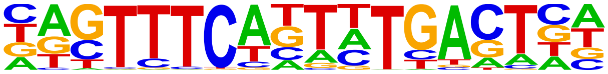	bZIP:IRF(bZIP,IRF)/Th17-BatF-ChIP-Seq(GSE39756)/Homer	1e-2	-5.797e+00	0.0085	254.0
359	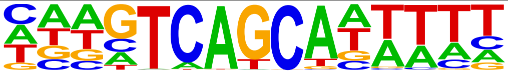	MafF(bZIP)/HepG2-MafF-ChIP-Seq(GSE31477)/Homer	1e-2	-5.796e+00	0.0085	220.0
360	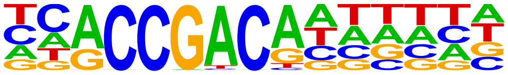	AT3G16280(AP2EREBP)/colamp-AT3G16280-DAP-Seq(GSE60143)/Homer	1e-2	-5.750e+00	0.0089	597.0
361	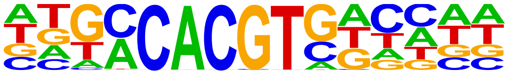	AREB3(bZIP)/col-AREB3-DAP-Seq(GSE60143)/Homer	1e-2	-5.682e+00	0.0095	387.0
362	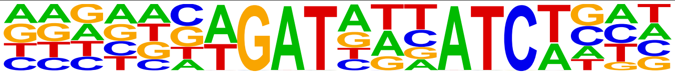	GATA(Zf),IR3/iTreg-Gata3-ChIP-Seq(GSE20898)/Homer	1e-2	-5.655e+00	0.0097	107.0
363	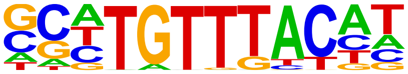	FOXK2(Forkhead)/U2OS-FOXK2-ChIP-Seq(E-MTAB-2204)/Homer	1e-2	-5.635e+00	0.0099	460.0
364	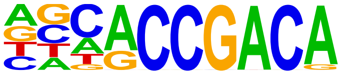	TINY(AP2EREBP)/col-TINY-DAP-Seq(GSE60143)/Homer	1e-2	-5.618e+00	0.0100	548.0
365	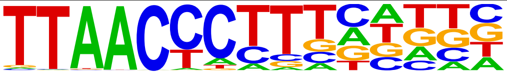	ZNF652/HepG2-ZNF652.Flag-ChIP-Seq(Encode)/Homer	1e-2	-5.585e+00	0.0103	184.0
366	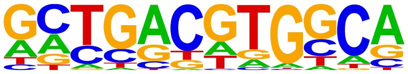	O2(bZIP)/Corn-O2-ChIP-Seq(GSE63991)/Homer	1e-2	-5.514e+00	0.0111	263.0
367		IRF4(IRF)/GM12878-IRF4-ChIP-	1e-2	-5.476e+00	0.0115	337.0

	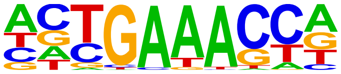	Seq(GSE32465)/Homer				
368	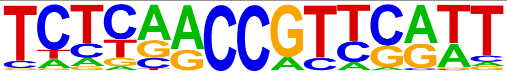	AT5G59990(C2C2COlike)/colamp-AT5G59990-DAP-Seq(GSE60143)/Homer	1e-2	-5.425e+00	0.0120	34.0
369	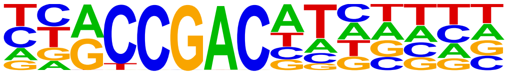	CBF2(AP2EREBP)/colamp-CBF2-DAP-Seq(GSE60143)/Homer	1e-2	-5.420e+00	0.0121	722.0
370	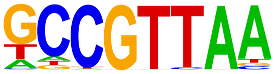	AT3G10030(Trihelix)/colamp-AT3G10030-DAP-Seq(GSE60143)/Homer	1e-2	-5.364e+00	0.0127	713.0
371	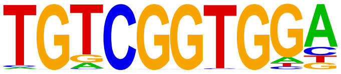	At1g77640(AP2EREBP)/col-At1g77640-DAP-Seq(GSE60143)/Homer	1e-2	-5.357e+00	0.0128	451.0
372	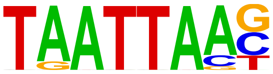	ATHB24(ZFHD)/colamp-ATHB24-DAP-Seq(GSE60143)/Homer	1e-2	-5.347e+00	0.0129	503.0
373	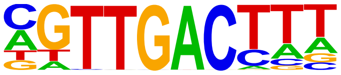	WRKY29(WRKY)/colamp-WRKY29-DAP-Seq(GSE60143)/Homer	1e-2	-5.324e+00	0.0131	504.0
374	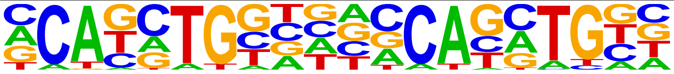	Twist(bHLH)/HMLE-TWIST1-ChIP-Seq(Chang_et_al)/Homer	1e-2	-5.255e+00	0.0141	158.0
375	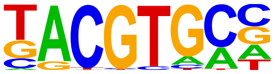	HIF-1a(bHLH)/MCF7-HIF1a-ChIP-Seq(GSE28352)/Homer	1e-2	-5.238e+00	0.0142	584.0
376	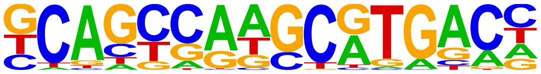	PAX5(Paired,Homeobox)/GM12878-PAX5-ChIP-Seq(GSE32465)/Homer	1e-2	-5.124e+00	0.0159	690.0
377	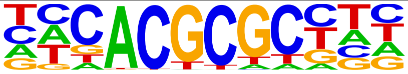	FHY3(FAR1)/Arabidopsis-FHY3-ChIP-Seq(GSE30711)/Homer	1e-2	-5.104e+00	0.0162	1035.0
378	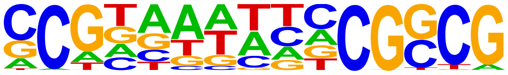	AS2(LOBAS2)/col-AS2-DAP-Seq(GSE60143)/Homer	1e-2	-5.077e+00	0.0166	592.0
379	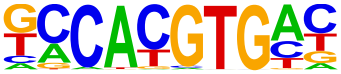	NPAS2(bHLH)/Liver-NPAS2-ChIP-Seq(GSE39860)/Homer	1e-2	-5.043e+00	0.0171	1250.0
380	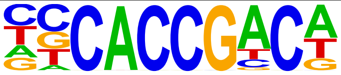	AT1G44830(AP2EREBP)/col-AT1G44830-DAP-Seq(GSE60143)/Homer	1e-2	-5.040e+00	0.0171	924.0
381	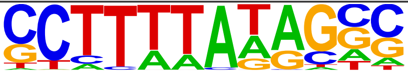	TATA-Box(TBP)/Promoter/Homer	1e-2	-4.952e+00	0.0187	966.0
382	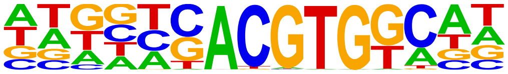	bZIP3(bZIP)/col-bZIP3-DAP-Seq(GSE60143)/Homer	1e-2	-4.949e+00	0.0187	514.0
383	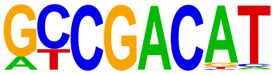	DDF1(AP2EREBP)/col-DDF1-DAP-Seq(GSE60143)/Homer	1e-2	-4.931e+00	0.0190	633.0
384	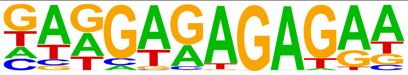	BPC1(BBRBPC)/colamp-BPC1-DAP-Seq(GSE60143)/Homer	1e-2	-4.887e+00	0.0198	503.0
385	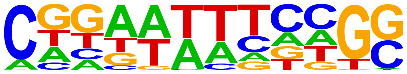	LBD18(LOBAS2)/colamp-LBD18-DAP-Seq(GSE60143)/Homer	1e-2	-4.843e+00	0.0206	2302.0
386	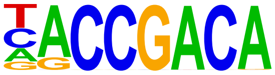	bHLH10(bHLH)/colamp-bHLH10-DAP-Seq(GSE60143)/Homer	1e-2	-4.822e+00	0.0210	579.0
387	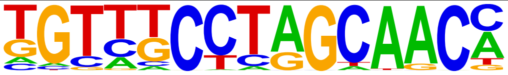	Rfx6(HTH)/Min6b1-Rfx6.HA-ChIP-Seq(GSE62844)/Homer	1e-2	-4.820e+00	0.0210	1517.0
388	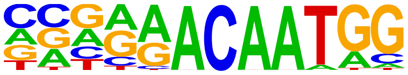	Sox7(HMG)/ESC-Sox7-ChIP-Seq(GSE133899)/Homer	1e-2	-4.733e+00	0.0228	275.0
389	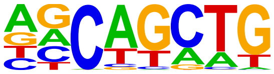	SCL(bHLH)/HPC7-Scl-ChIP-Seq(GSE13511)/Homer	1e-2	-4.643e+00	0.0249	6486.0
