# Supplementary material for: PBX1 and PBX3 transcription factors regulate SHH expression in the Frontonasal Ectodermal Zone through complementary mechanisms
Source: PLoS Genet. 2025 May 21;21(5):e1011315. doi: 10.1371/journal.pgen.1011315 (PMC12140432; doi:10.1371/journal.pgen.1011315)
Supplement: S4 Table — (PDF) [file pgen.1011315.s010.pdf]

S4 Table. Full list of de novo motif discovery from ChIP-seq data targeting PBX1.

Homer *de novo* Motif Results (/wynton/group/marcucio/2022CHM/Data2022Mar/Motif/HomerPBX1IDR/)

Known Motif Enrichment Results
Gene Ontology Enrichment Results
If Homer is having trouble matching a motif to a known motif, try copy/pasting the matrix file into STAMP
More information on motif finding results: HOMER | Description of Results | Tips
Total target sequences = 13431
Total background sequences = 36373
* - possible false positive

Rank	Motif	P-value	log P-pvalue	% of Targets	% of Background	STD(Bg STD)	Best Match/Details	Motif File
1	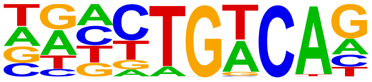	1e-823	-1.896e+03	29.90%	10.54%	49.0bp (62.6bp)	Tgif1(Homeobox)/mES-Tgif1-ChIP-Seq(GSE55404)/Homer(0.955) More Information | Similar Motifs Found	motif file (matrix)
2	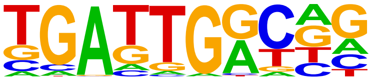	1e-480	-1.107e+03	18.00%	6.18%	53.0bp (64.4bp)	Pknox1(Homeobox)/ES-Prep1-ChIP-Seq(GSE63282)/Homer(0.903) More Information | Similar Motifs Found	motif file (matrix)
3	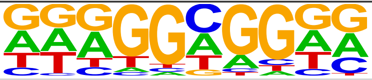	1e-158	-3.646e+02	44.58%	33.40%	54.8bp (62.0bp)	POL003.1_GC-box/Jaspar(0.867) More Information | Similar Motifs Found	motif file (matrix)
4	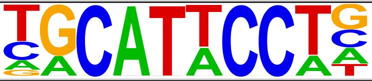	1e-121	-2.793e+02	4.85%	1.67%	55.2bp (59.7bp)	TEAD3/MA0808.1/Jaspar(0.930) More Information | Similar Motifs Found	motif file (matrix)
5	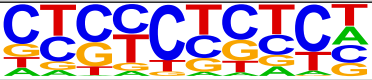	1e-105	-2.425e+02	42.83%	33.75%	55.9bp (62.4bp)	SeqBias: GA-repeat(0.797) More Information | Similar Motifs Found	motif file (matrix)
6	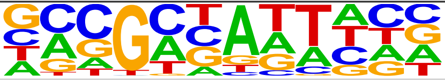	1e-86	-1.998e+02	42.19%	33.96%	55.5bp (63.5bp)	pho/dmmpmm(Bergman)/fly(0.716) More Information | Similar Motifs Found	motif file (matrix)
7	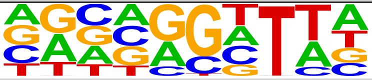	1e-82	-1.905e+02	18.13%	12.32%	56.8bp (61.4bp)	bcd/dmmpmm(Bigfoot)/fly(0.794) More Information | Similar Motifs Found	motif file (matrix)
8	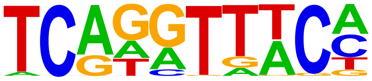	1e-75	-1.742e+02	4.10%	1.68%	55.3bp (61.2bp)	Six1(Homeobox)/Myoblast-Six1-ChIP-Chip(GSE20150)/Homer(0.935) More Information | Similar Motifs Found	motif file (matrix)
9	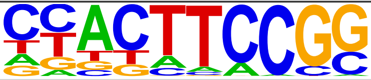	1e-65	-1.512e+02	8.83%	5.22%	53.0bp (59.3bp)	ELK4/MA0076.2/Jaspar(0.949) More Information | Similar Motifs Found	motif file (matrix)
10	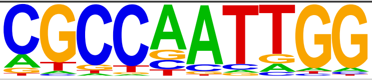	1e-65	-1.497e+02	5.36%	2.67%	54.0bp (64.5bp)	NFY(CCAAT)/Promoter/Homer(0.766) More Information | Similar Motifs Found	motif file (matrix)
11	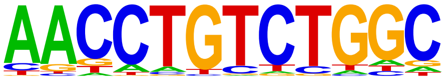	1e-50	-1.158e+02	1.33%	0.34%	53.3bp (53.7bp)	Smad2(MAD)/ES-SMAD2-ChIP-Seq(GSE29422)/Homer(0.772) More Information | Similar Motifs Found	motif file (matrix)
12	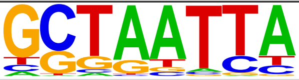	1e-48	-1.106e+02	21.64%	16.75%	57.5bp (62.1bp)	Ro/dmmpmm(Noyes_hd)/fly(0.910) More Information | Similar Motifs Found	motif file (matrix)
13	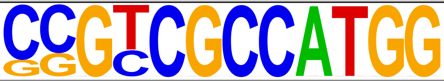	1e-45	-1.039e+02	10.92%	7.50%	52.0bp (60.4bp)	YY2/MA0748.2/Jaspar(0.799) More Information | Similar Motifs Found	motif file (matrix)
14	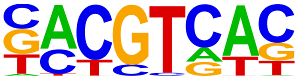	1e-43	-9.922e+01	10.28%	7.03%	55.0bp (64.2bp)	TGA9(bZIP)/colamp-TGA9-DAP-Seq(GSE60143)/Homer(0.908) More Information | Similar Motifs Found	motif file (matrix)
15	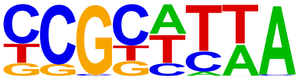	1e-41	-9.549e+01	21.90%	17.33%	55.9bp (65.0bp)	Unknown3/Arabidopsis-Promoters/Homer(0.702) More Information | Similar Motifs Found	motif file (matrix)
16	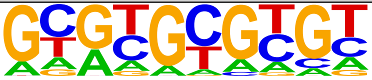	1e-30	-7.091e+01	13.58%	10.39%	55.7bp (67.4bp)	TCFL5/MA0632.2/Jaspar(0.775) More Information | Similar Motifs Found	motif file (matrix)
17	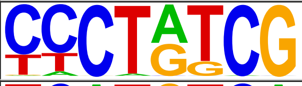	1e-29	-6.742e+01	8.82%	6.31%	57.3bp (64.6bp)	Dref/dmmpmm(Bigfoot)/fly(0.701) More Information | Similar Motifs Found	motif file (matrix)
18	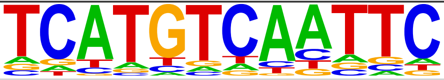	1e-28	-6.666e+01	0.16%	0.00%	46.3bp (1.7bp)	CG11617/MA0173.1/Jaspar(0.697) More Information | Similar Motifs Found	motif file (matrix)
							VRN1(ABI3VP1)/col-VRN1-DAP-	

19	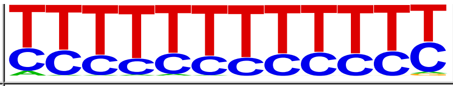	1e-25	-5.766e+01	2.61%	1.42%	57.2bp (72.3bp)	Seq(GSE60143)/Homer(0.853) More Information | Similar Motifs Found	motif file (matrix)
20	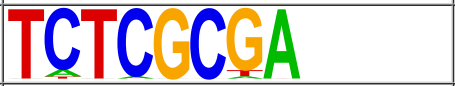	1e-23	-5.340e+01	1.46%	0.65%	54.7bp (64.8bp)	ZBTB33/MA0527.1/Jaspar(0.901) More Information | Similar Motifs Found	motif file (matrix)
21	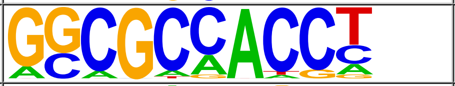	1e-14	-3.438e+01	3.61%	2.48%	53.9bp (59.4bp)	RPN4/MA0373.1/Jaspar(0.861) More Information | Similar Motifs Found	motif file (matrix)
22 *	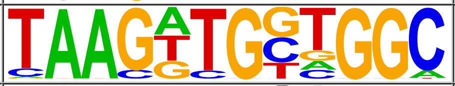	1e-10	-2.458e+01	0.29%	0.08%	62.3bp (7.2bp)	YY1(Zf)/Promoter/Homer(0.751) More Information | Similar Motifs Found	motif file (matrix)
23 *	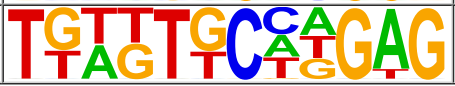	1e-4	-1.076e+01	0.06%	0.01%	66.4bp (44.7bp)	slbo/dmmpmm(Down)/fly(0.645) More Information | Similar Motifs Found	motif file (matrix)
