# Supplementary material for: PBX1 and PBX3 transcription factors regulate SHH expression in the Frontonasal Ectodermal Zone through complementary mechanisms
Source: PLoS Genet. 2025 May 21;21(5):e1011315. doi: 10.1371/journal.pgen.1011315 (PMC12140432; doi:10.1371/journal.pgen.1011315)
Supplement: S5 Table — (PDF) [file pgen.1011315.s011.pdf]

S5 Table. Full list of known motif discovery from ChIP-seq data targeting PBX3.

Homer Known Motif Enrichment Results (/wynton/group/marcucio/2022CHM/Data2022Mar/Motif/HomerPBX3IDR)

Homer *de novo* Motif Results
Gene Ontology Enrichment Results
Known Motif Enrichment Results (txt file)

Total Target Sequences = 36119, Total Background Sequences = 35379

Rank	Motif	Name	P-value	log P-pvalue	q-value (Benjamini)	# Target Sequences with Motif	% o Targ Sequ with Mot
1	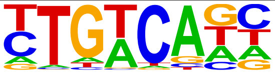	Tgif1(Homeobox)/mES-Tgif1-ChIP-Seq(GSE55404)/Homer	1e-2802	-6.454e+03	0.0000	23203.0	64.2
2	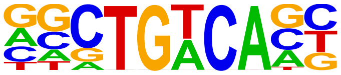	Meis1(Homeobox)/MastCells-Meis1-ChIP-Seq(GSE48085)/Homer	1e-2776	-6.394e+03	0.0000	16533.0	45.7
3	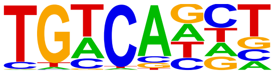	Tgif2(Homeobox)/mES-Tgif2-ChIP-Seq(GSE55404)/Homer	1e-2403	-5.535e+03	0.0000	23110.0	63.9
4	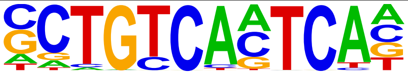	Pbx3(Homeobox)/GM12878-PBX3-ChIP-Seq(GSE32465)/Homer	1e-1661	-3.825e+03	0.0000	4067.0	11.2
5	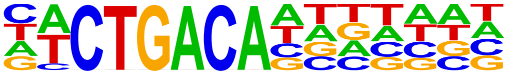	GRF9(GRF)/colamp-GRF9-DAP-Seq(GSE60143)/Homer	1e-1358	-3.129e+03	0.0000	9651.0	26.7
6	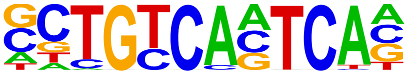	Pknox1(Homeobox)/ES-Prep1-ChIP-Seq(GSE63282)/Homer	1e-1346	-3.100e+03	0.0000	3558.0	9.85
7	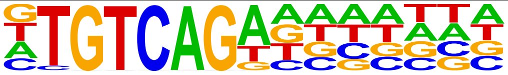	AtGRF6(GRF)/col-AtGRF6-DAP-Seq(GSE60143)/Homer	1e-1335	-3.076e+03	0.0000	10601.0	29.3
8	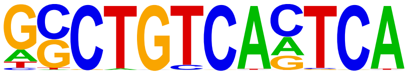	PBX1(Homeobox)/MCF7-PBX1-ChIP-Seq(GSE28007)/Homer	1e-807	-1.860e+03	0.0000	1628.0	4.51
9	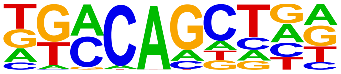	bZIP18(bZIP)/colamp-bZIP18-DAP-Seq(GSE60143)/Homer	1e-490	-1.129e+03	0.0000	25460.0	70.4
10	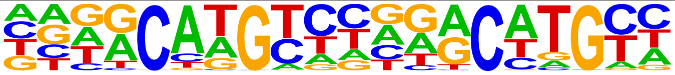	p63(p53)/Keratinocyte-p63-ChIP-Seq(GSE17611)/Homer	1e-481	-1.108e+03	0.0000	2822.0	7.81
11	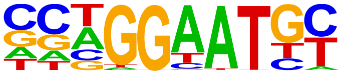	TEAD4(TEA)/Tropoblast-Tead4-ChIP-Seq(GSE37350)/Homer	1e-452	-1.043e+03	0.0000	4926.0	13.6
12	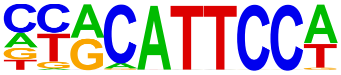	TEAD1(TEAD)/HepG2-TEAD1-ChIP-Seq(Encode)/Homer	1e-433	-9.985e+02	0.0000	5416.0	14.9
13	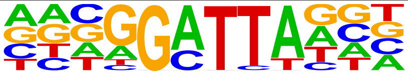	bcd(Homeobox)/Embryo-Bcd-ChIP-Seq(GSE86966)/Homer	1e-413	-9.524e+02	0.0000	6120.0	16.9
14	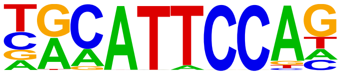	TEAD3(TEA)/HepG2-TEAD3-ChIP-Seq(Encode)/Homer	1e-400	-9.219e+02	0.0000	6105.0	16.9
15	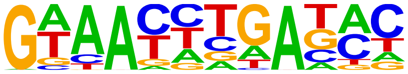	Six2(Homeobox)/NephronProgenitor-Six2-ChIP-Seq(GSE39837)/Homer	1e-393	-9.052e+02	0.0000	5459.0	15.1
16	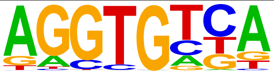	Tbx5(T-box)/HL1-Tbx5.biotin-ChIP-Seq(GSE21529)/Homer	1e-388	-8.941e+02	0.0000	15503.0	42.9
17	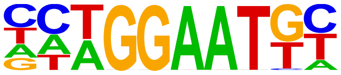	TEAD(TEA)/Fibroblast-PU.1-ChIP-Seq(Unpublished)/Homer	1e-365	-8.418e+02	0.0000	4086.0	11.3
18	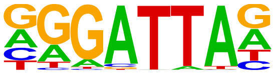	GSC(Homeobox)/FrogEmbryos-GSC-ChIP-Seq(DRA000576)/Homer	1e-362	-8.337e+02	0.0000	6170.0	17.0
19	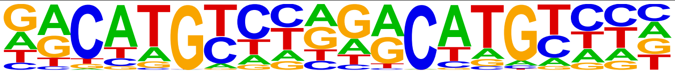	p53(p53)/Saos-p53-ChIP-Seq(GSE15780)/Homer	1e-339	-7.817e+02	0.0000	1122.0	3.11
20	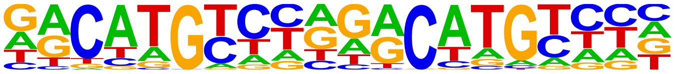	p53(p53)/Saos-p53-ChIP-Seq/Homer	1e-339	-7.817e+02	0.0000	1122.0	3.11

21	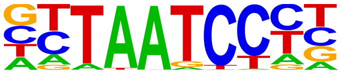	Otx2(Homeobox)/EpiLC-Otx2-ChIP-Seq(GSE56098)/Homer	1e-337	-7.764e+02	0.0000	4493.0	12.4
22	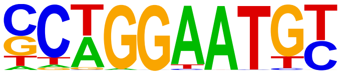	TEAD2(TEA)/Py2T-Tead2-ChIP-Seq(GSE55709)/Homer	1e-331	-7.637e+02	0.0000	3213.0	8.89
23	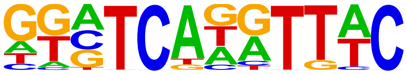	Six1(Homeobox)/Myoblast-Six1-ChIP-Chip(GSE20150)/Homer	1e-323	-7.441e+02	0.0000	1903.0	5.27
24	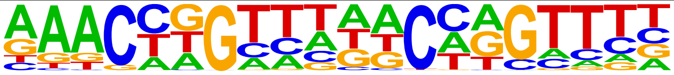	GRHL2(CP2)/HBE-GRHL2-ChIP-Seq(GSE46194)/Homer	1e-319	-7.359e+02	0.0000	2512.0	6.95
25	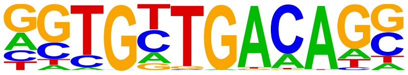	Tbx20(T-box)/Heart-Tbx20-ChIP-Seq(GSE29636)/Homer	1e-298	-6.867e+02	0.0000	2425.0	6.71
26	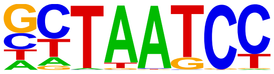	CRX(Homeobox)/Retina-Crx-ChIP-Seq(GSE20012)/Homer	1e-295	-6.797e+02	0.0000	10523.0	29.1
27	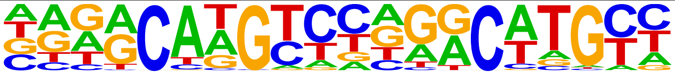	p73(p53)/Trachea-p73-ChIP-Seq(PRJNA310161)/Homer	1e-293	-6.747e+02	0.0000	705.0	1.95
28	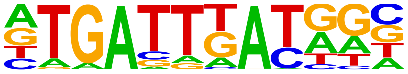	PBX2(Homeobox)/K562-PBX2-ChIP-Seq(Encode)/Homer	1e-281	-6.481e+02	0.0000	4956.0	13.7
29	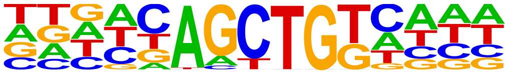	bZIP52(bZIP)/colamp-bZIP52-DAP-Seq(GSE60143)/Homer	1e-216	-4.992e+02	0.0000	8842.0	24.4
30	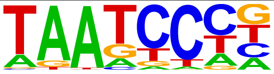	Pitx1(Homeobox)/Chicken-Pitx1-ChIP-Seq(GSE38910)/Homer	1e-197	-4.536e+02	0.0000	17625.0	48.7
31	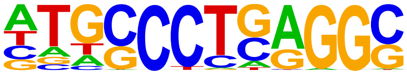	AP-2alpha(AP2)/Hela-AP2alpha-ChIP-Seq(GSE31477)/Homer	1e-189	-4.367e+02	0.0000	4148.0	11.4
32	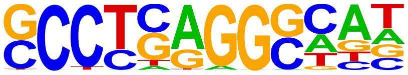	AP-2gamma(AP2)/MCF7-TFAP2C-ChIP-Seq(GSE21234)/Homer	1e-188	-4.345e+02	0.0000	5259.0	14.5
33	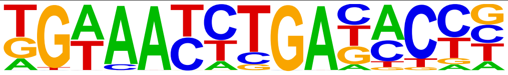	Six4(Homeobox)/MCF7-SIX4-ChIP-Seq(Encode)/Homer	1e-173	-3.992e+02	0.0000	503.0	1.39
34	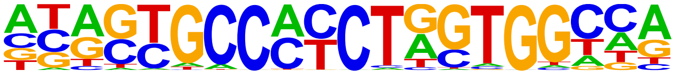	CTCF(Zf)/CD4+-CTCF-ChIP-Seq(Barski_et_al.)/Homer	1e-171	-3.944e+02	0.0000	970.0	2.69
35	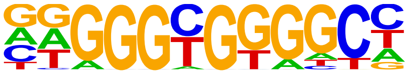	KLF1(Zf)/HUDEP2-KLF1-CutnRun(GSE136251)/Homer	1e-154	-3.569e+02	0.0000	3241.0	8.97
36	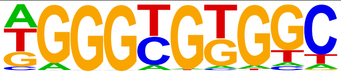	KLF5(Zf)/LoVo-KLF5-ChIP-Seq(GSE49402)/Homer	1e-150	-3.470e+02	0.0000	4594.0	12.7
37	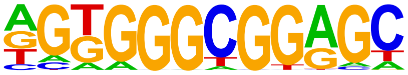	Sp5(Zf)/mES-Sp5.Flag-ChIP-Seq(GSE72989)/Homer	1e-150	-3.466e+02	0.0000	4064.0	11.2
38	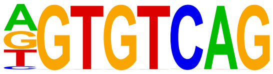	CELF2(RRM)/JSL1-CELF2-CLIP-Seq(GSE71264)/Homer	1e-150	-3.457e+02	0.0000	2478.0	6.86
39	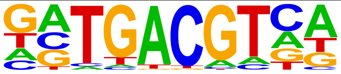	bZIP50(bZIP)/colamp-bZIP50-DAP-Seq(GSE60143)/Homer	1e-149	-3.448e+02	0.0000	4584.0	12.6
40	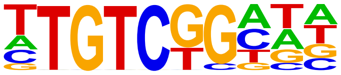	ARF2(ARF)/col-ARF2-DAP-Seq(GSE60143)/Homer	1e-139	-3.217e+02	0.0000	12355.0	34.2
41	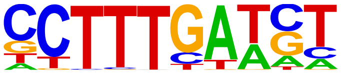	LEF1(HMG)/H1-LEF1-ChIP-Seq(GSE64758)/Homer	1e-126	-2.909e+02	0.0000	3720.0	10.3
42	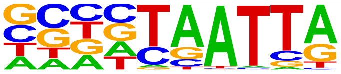	DLX1(Homeobox)/BasalGanglia-Dlx1-ChIP-seq(GSE124936)/Homer	1e-124	-2.874e+02	0.0000	6866.0	19.0
43	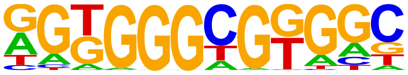	KLF14(Zf)/HEK293-KLF14.GFP-ChIP-Seq(GSE58341)/Homer	1e-124	-2.864e+02	0.0000	6592.0	18.2
44	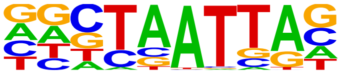	En1(Homeobox)/SUM149-EN1-ChIP-Seq(GSE120957)/Homer	1e-123	-2.842e+02	0.0000	8838.0	24.4
45	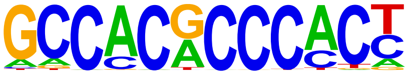	Klf9(Zf)/GBM-Klf9-ChIP-Seq(GSE62211)/Homer	1e-123	-2.833e+02	0.0000	1336.0	3.70

46	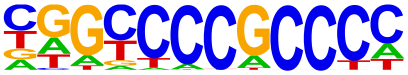	Sp2(Zf)/HEK293-Sp2.eGFP-ChIP-Seq(Encode)/Homer	1e-119	-2.753e+02	0.0000	5633.0	15.5
47	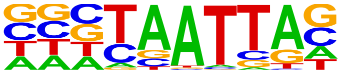	DLX2(Homeobox)/BasalGanglia-Dlx2-ChIP-seq(GSE124936)/Homer	1e-115	-2.671e+02	0.0000	7436.0	20.5
48	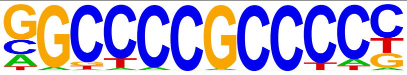	Sp1(Zf)/Promoter/Homer	1e-113	-2.606e+02	0.0000	1766.0	4.89
49	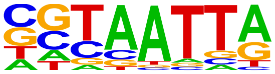	DLX5(Homeobox)/BasalGanglia-Dlx5-ChIP-seq(GSE124936)/Homer	1e-112	-2.590e+02	0.0000	4401.0	12.1
50	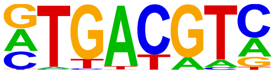	TGA9(bZIP)/colamp-TGA9-DAP-Seq(GSE60143)/Homer	1e-110	-2.536e+02	0.0000	5677.0	15.7
51	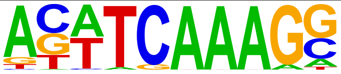	Tcf3(HMG)/mES-Tcf3-ChIP-Seq(GSE11724)/Homer	1e-109	-2.514e+02	0.0000	1627.0	4.50
52	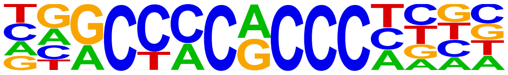	KLF3(Zf)/MEF-Klf3-ChIP-Seq(GSE44748)/Homer	1e-106	-2.444e+02	0.0000	1766.0	4.89
53	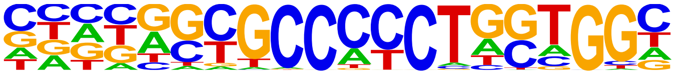	BORIS(Zf)/K562-CTCFL-ChIP-Seq(GSE32465)/Homer	1e-103	-2.380e+02	0.0000	1291.0	3.57
54	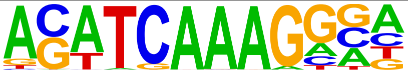	Tcf4(HMG)/Hct116-Tcf4-ChIP-Seq(SRA012054)/Homer	1e-99	-2.294e+02	0.0000	2596.0	7.19
55	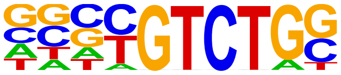	Smad4(MAD)/ESC-SMAD4-ChIP-Seq(GSE29422)/Homer	1e-98	-2.276e+02	0.0000	7191.0	19.9
56	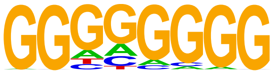	Maz(Zf)/HepG2-Maz-ChIP-Seq(GSE31477)/Homer	1e-96	-2.231e+02	0.0000	5625.0	15.5
57	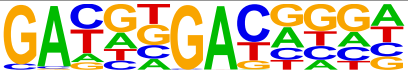	Knotted(Homeobox)/Corn-KN1-ChIP-Seq(GSE39161)/Homer	1e-89	-2.070e+02	0.0000	6418.0	17.7
58	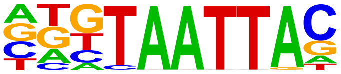	Dlx3(Homeobox)/Kerainocytes-Dlx3-ChIP-Seq(GSE89884)/Homer	1e-86	-2.002e+02	0.0000	3769.0	10.4
59	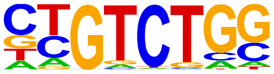	Smad2(MAD)/ES-SMAD2-ChIP-Seq(GSE29422)/Homer	1e-85	-1.980e+02	0.0000	7060.0	19.5
60	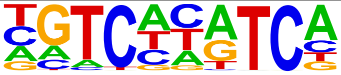	Replumless(BLH)/Arabidopsis-RPL.GFP-ChIP-Seq(GSE78727)/Homer	1e-84	-1.954e+02	0.0000	5015.0	13.8
61	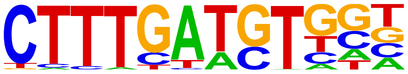	Tcf7(HMG)/GM12878-TCF7-ChIP-Seq(Encode)/Homer	1e-83	-1.933e+02	0.0000	1896.0	5.25
62	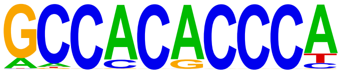	Klf4(Zf)/mES-Klf4-ChIP-Seq(GSE11431)/Homer	1e-83	-1.924e+02	0.0000	1008.0	2.79
63	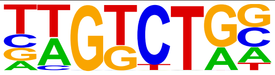	Smad3(MAD)/NPC-Smad3-ChIP-Seq(GSE36673)/Homer	1e-81	-1.868e+02	0.0000	11352.0	31.4
64	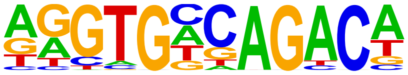	Tbox:Smad(T-box,MAD)/ESCd5-Smad2_3-ChIP-Seq(GSE29422)/Homer	1e-79	-1.825e+02	0.0000	1045.0	2.89
65	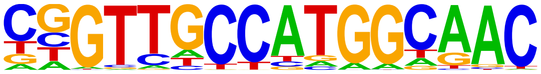	RFX(HTH)/K562-RFX3-ChIP-Seq(SRA012198)/Homer	1e-78	-1.811e+02	0.0000	533.0	1.48
66	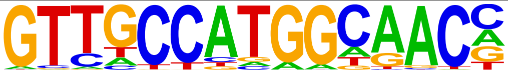	Rfx2(HTH)/LoVo-RFX2-ChIP-Seq(GSE49402)/Homer	1e-78	-1.810e+02	0.0000	553.0	1.53
67	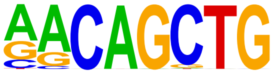	MyoG(bHLH)/C2C12-MyoG-ChIP-Seq(GSE36024)/Homer	1e-76	-1.760e+02	0.0000	6493.0	17.9
68	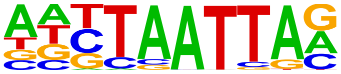	Lhx3(Homeobox)/Neuron-Lhx3-ChIP-Seq(GSE31456)/Homer	1e-76	-1.753e+02	0.0000	7897.0	21.8
69	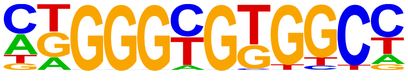	KLF6(Zf)/PDAC-KLF6-ChIP-Seq(GSE64557)/Homer	1e-75	-1.740e+02	0.0000	3988.0	11.0
70	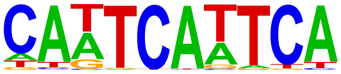	WUS1(Homeobox)/colamp-WUS1-DAP-Seq(GSE60143)/Homer	1e-74	-1.713e+02	0.0000	2141.0	5.93

71	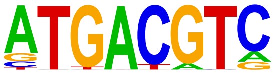	TGA10(bZIP)/colamp-TGA10-DAP-Seq(GSE60143)/Homer	1e-73	-1.684e+02	0.0000	2607.0	7.22
72	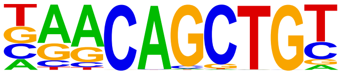	Myf5(bHLH)/GM-Myf5-ChIP-Seq(GSE24852)/Homer	1e-70	-1.628e+02	0.0000	4902.0	13.5
73	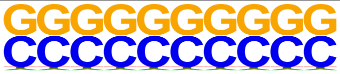	SeqBias: CG bias	1e-67	-1.556e+02	0.0000	25456.0	70.4
74	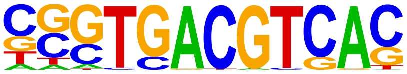	CRE(bZIP)/Promoter/Homer	1e-67	-1.551e+02	0.0000	1303.0	3.61
75	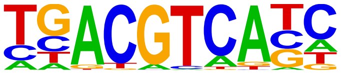	TGA6(bZIP)/colamp-TGA6-DAP-Seq(GSE60143)/Homer	1e-66	-1.537e+02	0.0000	2548.0	7.05
76	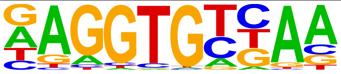	Tbx6(T-box)/ESC-Tbx6-ChIP-Seq(GSE93524)/Homer	1e-65	-1.498e+02	0.0000	4551.0	12.6
77	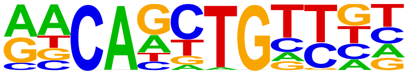	HLH-1(bHLH)/cElegans-Embryo-HLH1-ChIP-Seq(modEncode)/Homer	1e-64	-1.489e+02	0.0000	4296.0	11.8
78	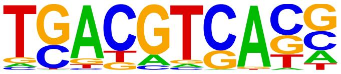	FEA4(bZIP)/Corn-FEA4-ChIP-Seq(GSE61954)/Homer	1e-64	-1.486e+02	0.0000	4827.0	13.3
79	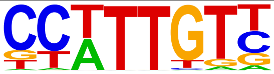	Sox3(HMG)/NPC-Sox3-ChIP-Seq(GSE33059)/Homer	1e-62	-1.434e+02	0.0000	7509.0	20.7
80	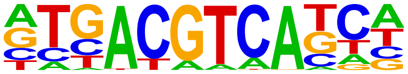	TGA4(bZIP)/colamp-TGA4-DAP-Seq(GSE60143)/Homer	1e-61	-1.426e+02	0.0000	1372.0	3.80
81	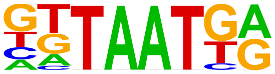	Nkx6.1(Homeobox)/Islet-Nkx6.1-ChIP-Seq(GSE40975)/Homer	1e-61	-1.421e+02	0.0000	11474.0	31.7
82	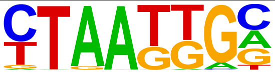	Isl1(Homeobox)/Neuron-Isl1-ChIP-Seq(GSE31456)/Homer	1e-61	-1.416e+02	0.0000	8315.0	23.0
83	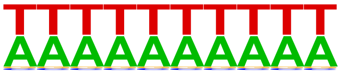	SeqBias: A/T bias	1e-60	-1.382e+02	0.0000	29583.0	81.9
84	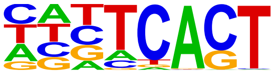	STZ(C2H2)/colamp-STZ-DAP-Seq(GSE60143)/Homer	1e-57	-1.323e+02	0.0000	23216.0	64.2
85	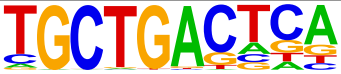	MafA(bZIP)/Islet-MafA-ChIP-Seq(GSE30298)/Homer	1e-55	-1.288e+02	0.0000	4885.0	13.5
86	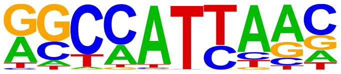	Nanog(Homeobox)/mES-Nanog-ChIP-Seq(GSE11724)/Homer	1e-55	-1.286e+02	0.0000	17174.0	47.5
87	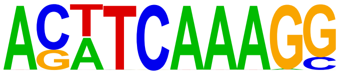	TCFL2(HMG)/K562-TCF7L2-ChIP-Seq(GSE29196)/Homer	1e-55	-1.284e+02	0.0000	595.0	1.65
88	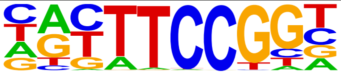	Elk4(ETS)/Hela-Elk4-ChIP-Seq(GSE31477)/Homer	1e-55	-1.283e+02	0.0000	1869.0	5.17
89	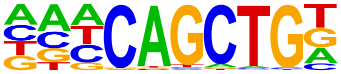	Ap4(bHLH)/AML-Tfap4-ChIP-Seq(GSE45738)/Homer	1e-54	-1.257e+02	0.0000	7498.0	20.7
90	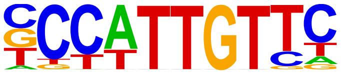	Sox2(HMG)/mES-Sox2-ChIP-Seq(GSE11431)/Homer	1e-53	-1.243e+02	0.0000	3733.0	10.3
91	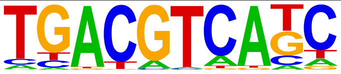	TGA1(bZIP)/colamp-TGA1-DAP-Seq(GSE60143)/Homer	1e-53	-1.243e+02	0.0000	1822.0	5.04
92	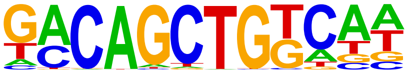	bZIP69(bZIP)/col-bZIP69-DAP-Seq(GSE60143)/Homer	1e-52	-1.220e+02	0.0000	952.0	2.64
93	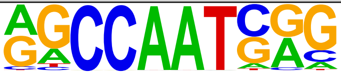	NFY(CCAAT)/Promoter/Homer	1e-52	-1.210e+02	0.0000	2758.0	7.64
94	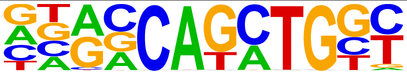	Atoh1(bHLH)/Cerebellum-Atoh1-ChIP-Seq(GSE22111)/Homer	1e-50	-1.160e+02	0.0000	5583.0	15.4
95	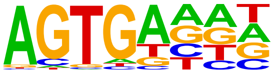	At5g04390(C2H2)/col200-At5g04390-DAP-Seq(GSE60143)/Homer	1e-49	-1.139e+02	0.0000	21818.0	60.4

96	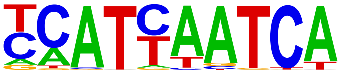	Pdx1(Homeobox)/Islet-Pdx1-ChIP-Seq(SRA008281)/Homer	1e-48	-1.119e+02	0.0000	4407.0	12.2
97	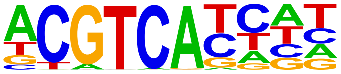	TGA2(bZIP)/colamp-TGA2-DAP-Seq(GSE60143)/Homer	1e-47	-1.095e+02	0.0000	2316.0	6.41
98	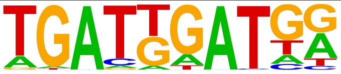	HOXA1(Homeobox)/mES-Hoxa1-ChIP-Seq(SRP084292)/Homer	1e-47	-1.089e+02	0.0000	1368.0	3.79
99	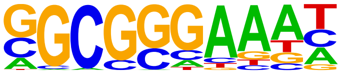	E2F4(E2F)/K562-E2F4-ChIP-Seq(GSE31477)/Homer	1e-45	-1.047e+02	0.0000	1858.0	5.14
100	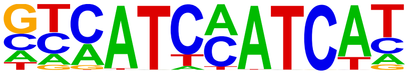	HOXA2(Homeobox)/mES-Hoxa2-ChIP-Seq(Donaldson_et_al.)/Homer	1e-44	-1.033e+02	0.0000	605.0	1.67
101	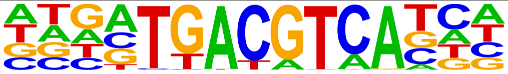	TGA5(bZIP)/col-TGA5-DAP-Seq(GSE60143)/Homer	1e-44	-1.021e+02	0.0000	450.0	1.25
102	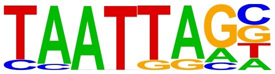	Lhx2(Homeobox)/HFSC-Lhx2-ChIP-Seq(GSE48068)/Homer	1e-44	-1.019e+02	0.0000	4926.0	13.6
103	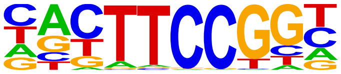	Elk1(ETS)/Hela-Elk1-ChIP-Seq(GSE31477)/Homer	1e-43	-1.004e+02	0.0000	1876.0	5.19
104	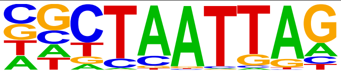	LHX9(Homeobox)/Hct116-LHX9.V5-ChIP-Seq(GSE116822)/Homer	1e-42	-9.748e+01	0.0000	6169.0	17.0
105	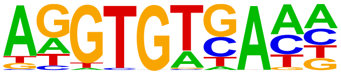	Tbet(T-box)/CD8-Tbet-ChIP-Seq(GSE33802)/Homer	1e-42	-9.699e+01	0.0000	4427.0	12.2
106	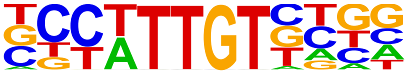	Sox21(HMG)/ESC-SOX21-ChIP-Seq(GSE110505)/Homer	1e-41	-9.658e+01	0.0000	7662.0	21.2
107	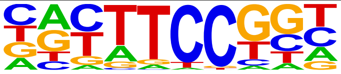	Fli1(ETS)/CD8-FLI-ChIP-Seq(GSE20898)/Homer	1e-41	-9.551e+01	0.0000	3925.0	10.8
108	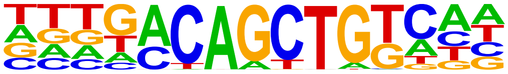	VIP1(bZIP)/col-VIP1-DAP-Seq(GSE60143)/Homer	1e-40	-9.430e+01	0.0000	1318.0	3.65
109	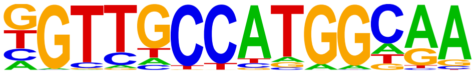	Rfx1(HTH)/NPC-H3K4me1-ChIP-Seq(GSE16256)/Homer	1e-40	-9.318e+01	0.0000	864.0	2.39
110	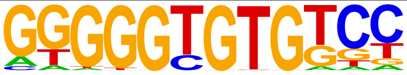	KLF10(Zf)/HEK293-KLF10.GFP-ChIP-Seq(GSE58341)/Homer	1e-40	-9.228e+01	0.0000	1596.0	4.42
111	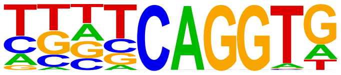	NGA4(ABI3VP1)/col-NGA4-DAP-Seq(GSE60143)/Homer	1e-39	-9.172e+01	0.0000	10590.0	29.3
112	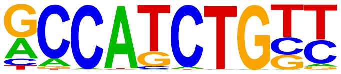	NeuroD1(bHLH)/Islet-NeuroD1-ChIP-Seq(GSE30298)/Homer	1e-38	-8.883e+01	0.0000	3783.0	10.4
113	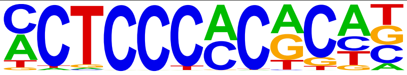	WT1(Zf)/Kidney-WT1-ChIP-Seq(GSE90016)/Homer	1e-38	-8.872e+01	0.0000	2142.0	5.93
114	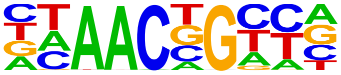	BMYB(HTH)/Hela-BMYB-ChIP-Seq(GSE27030)/Homer	1e-37	-8.690e+01	0.0000	7346.0	20.3
115	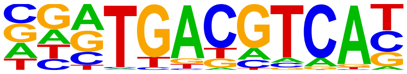	Atf7(bZIP)/3T3L1-Atf7-ChIP-Seq(GSE56872)/Homer	1e-35	-8.240e+01	0.0000	1860.0	5.15
116	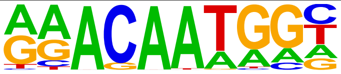	Sox15(HMG)/CPA-Sox15-ChIP-Seq(GSE62909)/Homer	1e-35	-8.131e+01	0.0000	4115.0	11.3
117	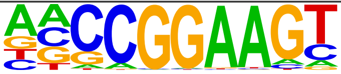	ELF1(ETS)/Jurkat-ELF1-ChIP-Seq(SRA014231)/Homer	1e-34	-8.050e+01	0.0000	1590.0	4.40
118	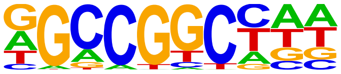	RAP211(AP2EREBP)/colamp-RAP211-DAP-Seq(GSE60143)/Homer	1e-34	-8.016e+01	0.0000	8739.0	24.1
119	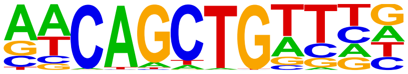	E-box/Drosophila-Promoters/Homer	1e-34	-7.967e+01	0.0000	1683.0	4.66
120		AMYB(HTH)/Testes-AMYB-ChIP-Seq(GSE44588)/Homer	1e-34	-7.962e+01	0.0000	7289.0	20.1

	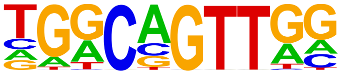						
121	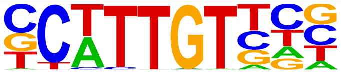	Sox10(HMG)/SciaticNerve-Sox3-ChIP-Seq(GSE35132)/Homer	1e-34	-7.944e+01	0.0000	7121.0	19.7
122	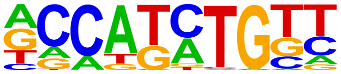	Olig2(bHLH)/Neuron-Olig2-ChIP-Seq(GSE30882)/Homer	1e-34	-7.925e+01	0.0000	8862.0	24.5
123	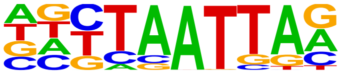	Lhx1(Homeobox)/EmbryoCarcinoma-Lhx1-ChIP-Seq(GSE70957)/Homer	1e-33	-7.802e+01	0.0000	5189.0	14.3
124	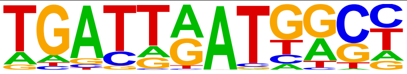	Hoxb4(Homeobox)/ES-Hoxb4-ChIP-Seq(GSE34014)/Homer	1e-33	-7.784e+01	0.0000	1021.0	2.83
125	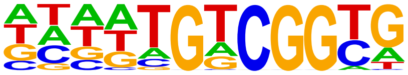	AT1G71450(AP2EREBP)/col-AT1G71450-DAP-Seq(GSE60143)/Homer	1e-33	-7.783e+01	0.0000	5587.0	15.4
126	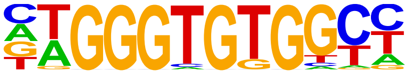	EKLF(Zf)/Erythrocyte-Klf1-ChIP-Seq(GSE20478)/Homer	1e-33	-7.759e+01	0.0000	417.0	1.15
127	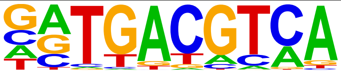	Atf1(bZIP)/K562-ATF1-ChIP-Seq(GSE31477)/Homer	1e-33	-7.754e+01	0.0000	2731.0	7.56
128	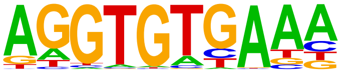	Tbx21(T-box)/GM12878-TBX21-ChIP-Seq(Encode)/Homer	1e-33	-7.681e+01	0.0000	4057.0	11.2
129	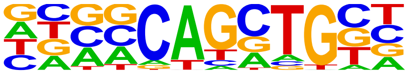	Ascl1(bHLH)/NeuralTubes-Ascl1-ChIP-Seq(GSE55840)/Homer	1e-33	-7.628e+01	0.0000	7581.0	20.9
130	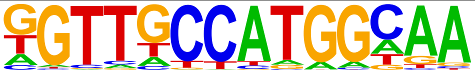	X-box(HTH)/NPC-H3K4me1-ChIP-Seq(GSE16256)/Homer	1e-32	-7.578e+01	0.0000	521.0	1.44
131	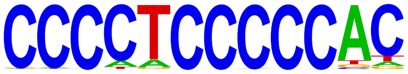	Zfp281(Zf)/ES-Zfp281-ChIP-Seq(GSE81042)/Homer	1e-32	-7.567e+01	0.0000	605.0	1.67
132	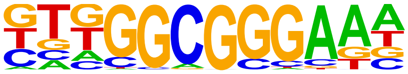	E2F3(E2F)/MEF-E2F3-ChIP-Seq(GSE71376)/Homer	1e-32	-7.513e+01	0.0000	2605.0	7.21
133	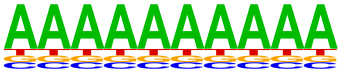	SeqBias: polyA-repeat	1e-31	-7.356e+01	0.0000	34471.0	95.4
134	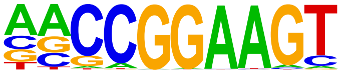	ETS(ETS)/Promoter/Homer	1e-31	-7.270e+01	0.0000	1037.0	2.87
135	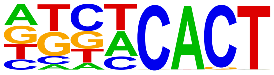	AZF1(C2H2)/colamp-AZF1-DAP-Seq(GSE60143)/Homer	1e-31	-7.245e+01	0.0000	19774.0	54.7
136	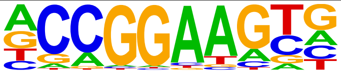	ETV4(ETS)/HepG2-ETV4-ChIP-Seq(ENCODE)/Homer	1e-31	-7.152e+01	0.0000	3826.0	10.5
137	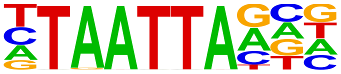	ATHB23(ZFHD)/col-ATHB23-DAP-Seq(GSE60143)/Homer	1e-31	-7.141e+01	0.0000	3993.0	11.0
138	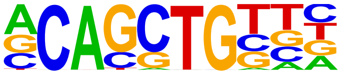	Ptf1a(bHLH)/Panc1-Ptf1a-ChIP-Seq(GSE47459)/Homer	1e-29	-6.870e+01	0.0000	12657.0	35.0
139	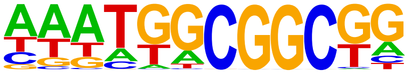	ABR1(AP2EREBP)/colamp-ABR1-DAP-Seq(GSE60143)/Homer	1e-29	-6.864e+01	0.0000	4538.0	12.5
140	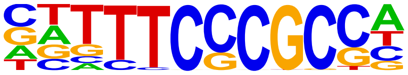	E2F7(E2F)/Hela-E2F7-ChIP-Seq(GSE32673)/Homer	1e-29	-6.832e+01	0.0000	493.0	1.36
141	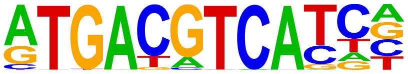	JunD(bZIP)/K562-JunD-ChIP-Seq/Homer	1e-29	-6.824e+01	0.0000	397.0	1.10
142	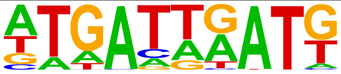	LIN-39(Homeobox)/cElegans.L3-LIN39-ChIP-Seq(modEncode)/Homer	1e-28	-6.465e+01	0.0000	4886.0	13.5
143	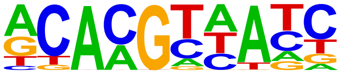	ANAC038(NAC)/col-ANAC038-DAP-Seq(GSE60143)/Homer	1e-27	-6.403e+01	0.0000	8625.0	23.8
144	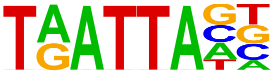	ATHB25(ZFHD)/colamp-ATHB25-DAP-Seq(GSE60143)/Homer	1e-27	-6.275e+01	0.0000	4790.0	13.2

145	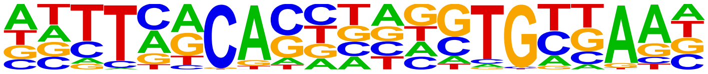	Brachyury(T-box)/Mesoendoderm-Brachyury-ChIP-exo(GSE54963)/Homer	1e-27	-6.261e+01	0.0000	1469.0	4.07
146	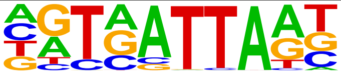	ATHB33(ZFHD)/col-ATHB33-DAP-Seq(GSE60143)/Homer	1e-27	-6.219e+01	0.0000	5753.0	15.9
147	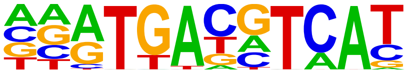	CREB5(bZIP)/LNCaP-CREB5.V5-ChIP-Seq(GSE137775)/Homer	1e-26	-6.196e+01	0.0000	1448.0	4.01
148	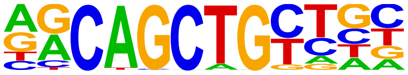	MyoD(bHLH)/Myotube-MyoD-ChIP-Seq(GSE21614)/Homer	1e-26	-6.194e+01	0.0000	5225.0	14.4
149	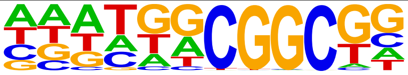	RAP26(AP2EREBP)/colamp-RAP26-DAP-Seq(GSE60143)/Homer	1e-26	-6.126e+01	0.0000	5585.0	15.4
150	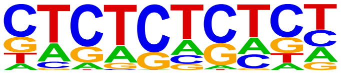	GAGA-repeat/Arabidopsis-Promoters/Homer	1e-26	-6.121e+01	0.0000	4064.0	11.2
151	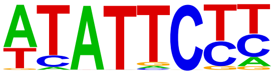	KAN2(G2like)/colamp-KAN2-DAP-Seq(GSE60143)/Homer	1e-26	-6.038e+01	0.0000	5007.0	13.8
152	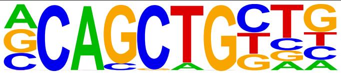	Tcf12(bHLH)/GM12878-Tcf12-ChIP-Seq(GSE32465)/Homer	1e-26	-6.010e+01	0.0000	5755.0	15.9
153	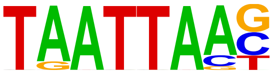	ATHB24(ZFHD)/colamp-ATHB24-DAP-Seq(GSE60143)/Homer	1e-26	-5.991e+01	0.0000	3220.0	8.91
154	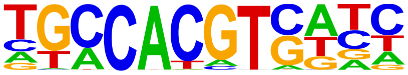	GBF3(bZIP)/Arabidopsis-GBF3-ChIP-Seq(GSE80564)/Homer	1e-25	-5.926e+01	0.0000	1609.0	4.45
155	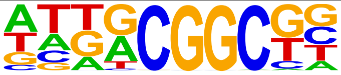	ERF115(AP2EREBP)/colamp-ERF115-DAP-Seq(GSE60143)/Homer	1e-25	-5.911e+01	0.0000	6863.0	19.0
156	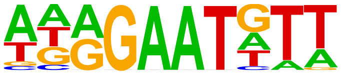	AT2G38300(G2like)/col-AT2G38300-DAP-Seq(GSE60143)/Homer	1e-25	-5.878e+01	0.0000	4942.0	13.6
157	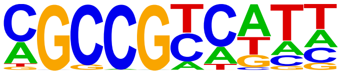	ERF8(AP2EREBP)/colamp-ERF8-DAP-Seq(GSE60143)/Homer	1e-25	-5.771e+01	0.0000	4929.0	13.6
158	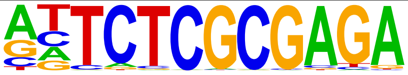	GFX(?)/Promoter/Homer	1e-24	-5.700e+01	0.0000	94.0	0.26
159	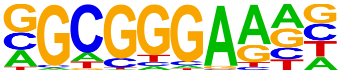	E2F6(E2F)/Hela-E2F6-ChIP-Seq(GSE31477)/Homer	1e-24	-5.629e+01	0.0000	2378.0	6.58
160	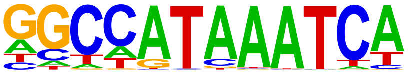	Hoxc9(Homeobox)/Ainv15-Hoxc9-ChIP-Seq(GSE21812)/Homer	1e-24	-5.588e+01	0.0000	2280.0	6.31
161	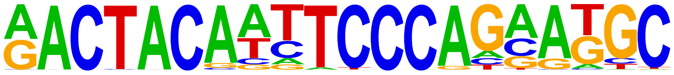	GFY-Staf(?,Zf)/Promoter/Homer	1e-24	-5.585e+01	0.0000	251.0	0.69
162	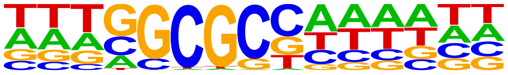	E2FA(E2FDP)/colamp-E2FA-DAP-Seq(GSE60143)/Homer	1e-24	-5.566e+01	0.0000	1035.0	2.87
163	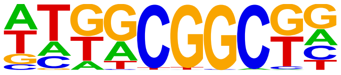	At2g33710(AP2EREBP)/colamp-At2g33710-DAP-Seq(GSE60143)/Homer	1e-24	-5.563e+01	0.0000	7871.0	21.7
164	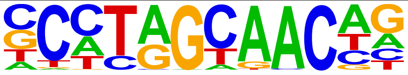	Rfx5(HTH)/GM12878-Rfx5-ChIP-Seq(GSE31477)/Homer	1e-24	-5.549e+01	0.0000	1164.0	3.22
165	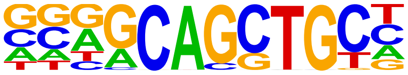	Ascl2(bHLH)/ESC-Ascl2-ChIP-Seq(GSE97712)/Homer	1e-24	-5.541e+01	0.0000	5971.0	16.5
166	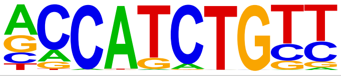	NeuroG2(bHLH)/Fibroblast-NeuroG2-ChIP-Seq(GSE75910)/Homer	1e-23	-5.511e+01	0.0000	7200.0	19.9
167	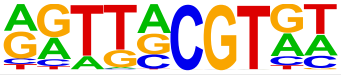	NAM(NAC)/col-NAM-DAP-Seq(GSE60143)/Homer	1e-23	-5.511e+01	0.0000	4261.0	11.8
168	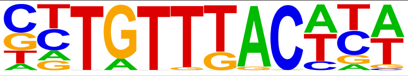	Foxa2(Forkhead)/Liver-Foxa2-ChIP-Seq(GSE25694)/Homer	1e-23	-5.465e+01	0.0000	3249.0	8.99
169	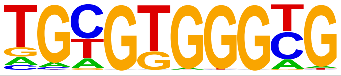	Egr1(Zf)/K562-Egr1-ChIP-Seq(GSE32465)/Homer	1e-23	-5.444e+01	0.0000	2233.0	6.18

170	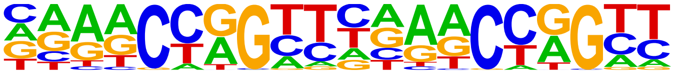	Tcfcp2l1(CP2)/mES-Tcfcp2l1-ChIP-Seq(GSE11431)/Homer	1e-23	-5.444e+01	0.0000	520.0	1.44
171	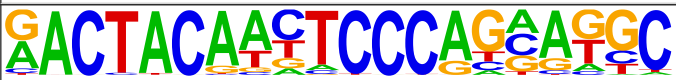	Ronin(THAP)/ES-Thap11-ChIP-Seq(GSE51522)/Homer	1e-23	-5.405e+01	0.0000	179.0	0.50
172	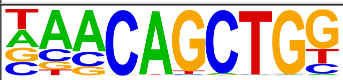	Tcf21(bHLH)/ArterySmoothMuscle-Tcf21-ChIP-Seq(GSE61369)/Homer	1e-23	-5.346e+01	0.0000	5371.0	14.8
173	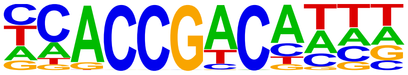	DEAR2(AP2EREBP)/colamp-DEAR2-DAP-Seq(GSE60143)/Homer	1e-23	-5.335e+01	0.0000	3046.0	8.43
174	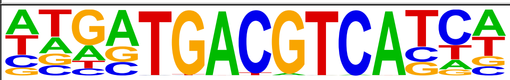	TGA3(bZIP)/colamp-TGA3-DAP-Seq(GSE60143)/Homer	1e-22	-5.274e+01	0.0000	276.0	0.76
175	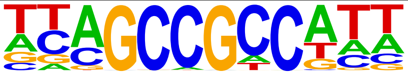	ERF13(AP2EREBP)/colamp-ERF13-DAP-Seq(GSE60143)/Homer	1e-22	-5.249e+01	0.0000	5467.0	15.1
176	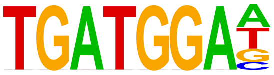	HAP3(CCAATHAP3)/col-HAP3-DAP-Seq(GSE60143)/Homer	1e-22	-5.243e+01	0.0000	1575.0	4.36
177	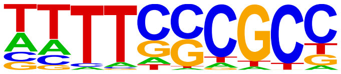	DEL2(E2FDP)/col-DEL2-DAP-Seq(GSE60143)/Homer	1e-22	-5.197e+01	0.0000	1297.0	3.59
178	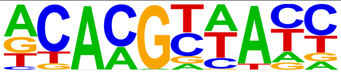	ANAC046(NAC)/colamp-ANAC046-DAP-Seq(GSE60143)/Homer	1e-22	-5.112e+01	0.0000	7723.0	21.3
179	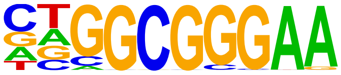	E2F1(E2F)/Hela-E2F1-ChIP-Seq(GSE22478)/Homer	1e-21	-5.052e+01	0.0000	1046.0	2.90
180	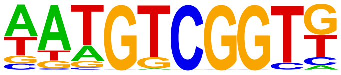	DREB19(AP2EREBP)/colamp-DREB19-DAP-Seq(GSE60143)/Homer	1e-21	-5.043e+01	0.0000	1624.0	4.50
181	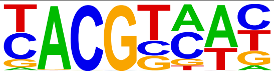	ATAF1(NAC)/col-ATAF1-DAP-Seq(GSE60143)/Homer	1e-21	-4.993e+01	0.0000	9614.0	26.6
182	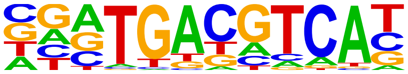	Atf2(bZIP)/3T3L1-Atf2-ChIP-Seq(GSE56872)/Homer	1e-21	-4.972e+01	0.0000	1240.0	3.43
183	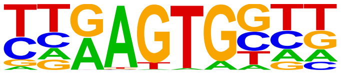	Bapx1(Homeobox)/VertebralCol-Bapx1-ChIP-Seq(GSE36672)/Homer	1e-21	-4.891e+01	0.0000	7819.0	21.6
184	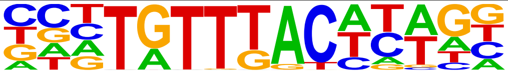	Foxa3(Forkhead)/Liver-Foxa3-ChIP-Seq(GSE77670)/Homer	1e-21	-4.889e+01	0.0000	1270.0	3.52
185	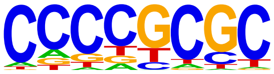	SUT1?/SacCer-Promoters/Homer	1e-21	-4.855e+01	0.0000	20912.0	57.8
186	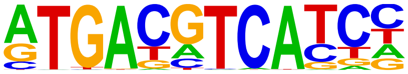	c-Jun-CRE(bZIP)/K562-cJun-ChIP-Seq(GSE31477)/Homer	1e-20	-4.819e+01	0.0000	1143.0	3.16
187	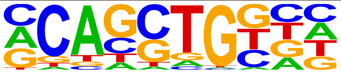	HEB(bHLH)/mES-Heb-ChIP-Seq(GSE53233)/Homer	1e-20	-4.776e+01	0.0000	8747.0	24.2
188	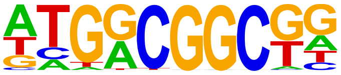	ERF7(AP2EREBP)/col-ERF7-DAP-Seq(GSE60143)/Homer	1e-20	-4.746e+01	0.0000	5686.0	15.7
189	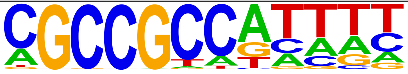	RAP212(AP2EREBP)/col-RAP212-DAP-Seq(GSE60143)/Homer	1e-20	-4.727e+01	0.0000	3735.0	10.3
190	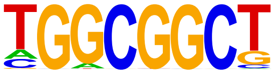	ERF105(AP2EREBP)/colamp-ERF105-DAP-Seq(GSE60143)/Homer	1e-20	-4.685e+01	0.0000	6552.0	18.1
191	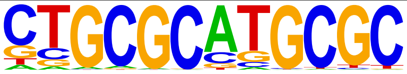	NRF1(NRF)/MCF7-NRF1-ChIP-Seq(Unpublished)/Homer	1e-19	-4.603e+01	0.0000	642.0	1.78
192	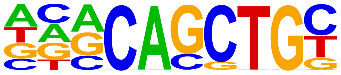	E2A(bHLH)/proBcell-E2A-ChIP-Seq(GSE21978)/Homer	1e-19	-4.570e+01	0.0000	7091.0	19.6
193	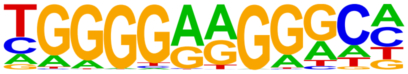	ZNF467(Zf)/HEK293-ZNF467.GFP-ChIP-Seq(GSE58341)/Homer	1e-19	-4.378e+01	0.0000	3227.0	8.93
194	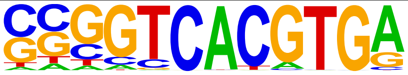	E-box(bHLH)/Promoter/Homer	1e-18	-4.375e+01	0.0000	335.0	0.93

195	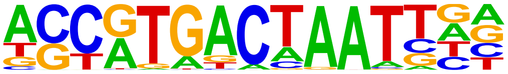	PAX3:FKHR-fusion(Paired,Homeobox)/Rh4-PAX3:FKHR-ChIP-Seq(GSE19063)/Homer	1e-18	-4.372e+01	0.0000	878.0	2.43
196	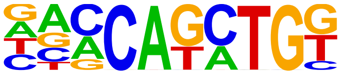	BHLHA15(bHLH)/NIH3T3-BHLHB8.HA-ChIP-Seq(GSE119782)/Homer	1e-18	-4.331e+01	0.0000	6718.0	18.6
197	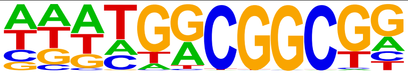	ERF4(AP2EREBP)/colamp-ERF4-DAP-Seq(GSE60143)/Homer	1e-18	-4.329e+01	0.0000	4931.0	13.6
198	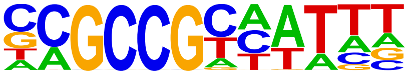	RRTF1(AP2EREBP)/colamp-RRTF1-DAP-Seq(GSE60143)/Homer	1e-18	-4.316e+01	0.0000	1161.0	3.21
199	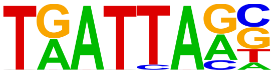	ATHB34(ZFHD)/colamp-ATHB34-DAP-Seq(GSE60143)/Homer	1e-18	-4.249e+01	0.0000	3375.0	9.34
200	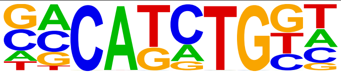	TCF4(bHLH)/SHSY5Y-TCF4-ChIP-Seq(GSE96915)/Homer	1e-18	-4.244e+01	0.0000	6900.0	19.1
201	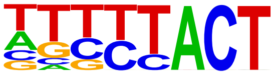	At3g60580(C2H2)/col-At3g60580-DAP-Seq(GSE60143)/Homer	1e-18	-4.225e+01	0.0000	19050.0	52.7
202	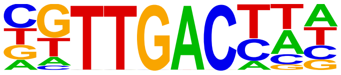	WRKY28(WRKY)/col-WRKY28-DAP-Seq(GSE60143)/Homer	1e-18	-4.205e+01	0.0000	3630.0	10.0
203	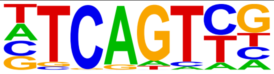	Initiator/Drosophila-Promoters/Homer	1e-18	-4.185e+01	0.0000	9692.0	26.8
204	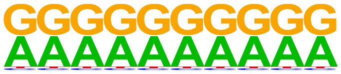	SeqBias: G/A bias	1e-18	-4.169e+01	0.0000	36117.0	99.9
205	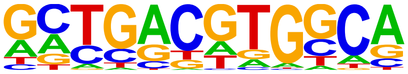	O2(bZIP)/Corn-O2-ChIP-Seq(GSE63991)/Homer	1e-17	-4.141e+01	0.0000	684.0	1.89
206	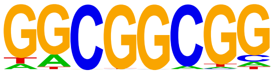	AT1G28160(AP2EREBP)/colamp-AT1G28160-DAP-Seq(GSE60143)/Homer	1e-17	-4.133e+01	0.0000	7686.0	21.2
207	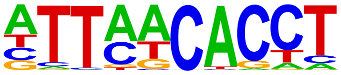	Eomes(T-box)/H9-Eomes-ChIP-Seq(GSE26097)/Homer	1e-17	-4.110e+01	0.0000	8535.0	23.6
208	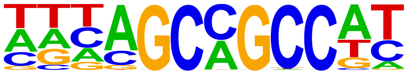	ERF15(AP2EREBP)/colamp-ERF15-DAP-Seq(GSE60143)/Homer	1e-17	-4.086e+01	0.0000	10531.0	29.1
209	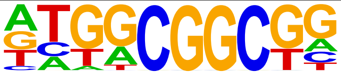	ERF11(AP2EREBP)/col-ERF11-DAP-Seq(GSE60143)/Homer	1e-17	-4.049e+01	0.0000	4449.0	12.3
210	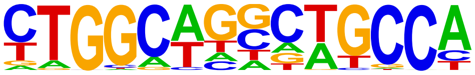	Tlx?(NR)/NPC-H3K4me1-ChIP-Seq(GSE16256)/Homer	1e-17	-4.030e+01	0.0000	1535.0	4.25
211	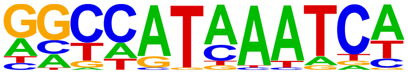	HOXA9(Homeobox)/HSC-Hoxa9-ChIP-Seq(GSE33509)/Homer	1e-17	-4.025e+01	0.0000	2888.0	8.00
212	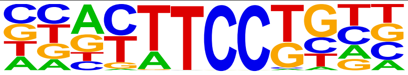	Etv2(ETS)/ES-ER71-ChIP-Seq(GSE59402)/Homer	1e-17	-3.985e+01	0.0000	3219.0	8.91
213	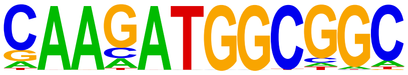	YY1(Zf)/Promoter/Homer	1e-17	-3.957e+01	0.0000	425.0	1.18
214	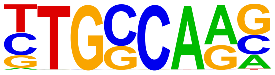	NF1-halfsite(CTF)/LNCaP-NF1-ChIP-Seq(Unpublished)/Homer	1e-17	-3.931e+01	0.0000	7495.0	20.7
215	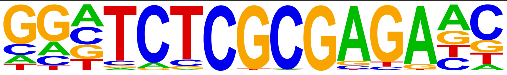	ZBTB33(Zf)/GM12878-ZBTB33-ChIP-Seq(GSE32465)/Homer	1e-16	-3.844e+01	0.0000	194.0	0.54
216	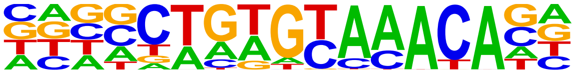	Fox:Ebox(Forkhead,bHLH)/Panc1-Foxa2-ChIP-Seq(GSE47459)/Homer	1e-16	-3.765e+01	0.0000	4143.0	11.4
217	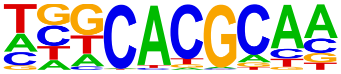	Arnt:Ahr(bHLH)/MCF7-Arnt-ChIP-Seq(Lo_et_al.)/Homer	1e-16	-3.753e+01	0.0000	3293.0	9.12
218	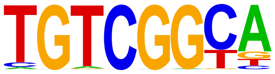	AT1G12630(AP2EREBP)/colamp-AT1G12630-DAP-Seq(GSE60143)/Homer	1e-16	-3.737e+01	0.0000	1453.0	4.02
219	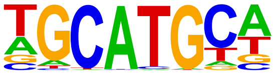	RBFox2(?)/Heart-RBFox2-CLIP-Seq(GSE57926)/Homer	1e-16	-3.698e+01	0.0000	9181.0	25.4

220	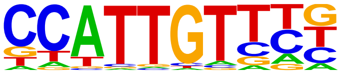	Sox17(HMG)/Endoderm-Sox17-ChIP-Seq(GSE61475)/Homer	1e-16	-3.698e+01	0.0000	2594.0	7.18
221	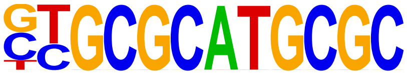	NRF(NRF)/Promoter/Homer	1e-15	-3.664e+01	0.0000	662.0	1.83
222	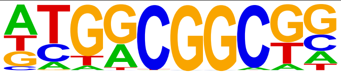	ERF3(AP2EREBP)/colamp-ERF3-DAP-Seq(GSE60143)/Homer	1e-15	-3.660e+01	0.0000	3806.0	10.5
223	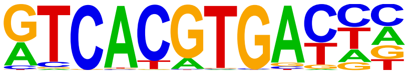	TFE3(bHLH)/MEF-TFE3-ChIP-Seq(GSE75757)/Homer	1e-15	-3.653e+01	0.0000	284.0	0.79
224	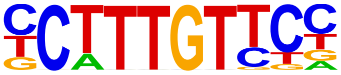	Sox4(HMG)/proB-Sox4-ChIP-Seq(GSE50066)/Homer	1e-15	-3.644e+01	0.0000	3599.0	9.96
225	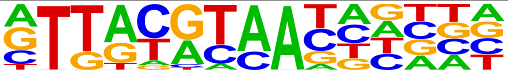	NFIL3(bZIP)/HepG2-NFIL3-ChIP-Seq(Encode)/Homer	1e-15	-3.620e+01	0.0000	2465.0	6.82
226	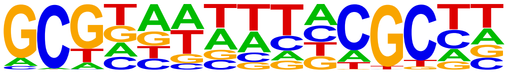	LBD23(LOBAS2)/colamp-LBD23-DAP-Seq(GSE60143)/Homer	1e-15	-3.542e+01	0.0000	4224.0	11.6
227	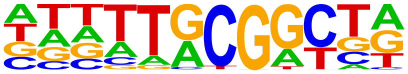	At5g08750(C3H)/col-At5g08750-DAP-Seq(GSE60143)/Homer	1e-15	-3.516e+01	0.0000	1534.0	4.25
228	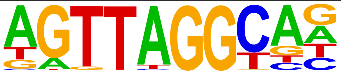	MYB116(MYB)/colamp-MYB116-DAP-Seq(GSE60143)/Homer	1e-15	-3.508e+01	0.0000	2088.0	5.78
229	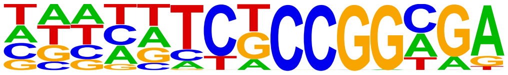	AT5G05550(Trihelix)/col-AT5G05550-DAP-Seq(GSE60143)/Homer	1e-15	-3.504e+01	0.0000	5123.0	14.1
230	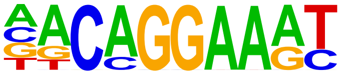	EWS:FLI1-fusion(ETS)/SK_N_MC-EWS:FLI1-ChIP-Seq(SRA014231)/Homer	1e-14	-3.415e+01	0.0000	1973.0	5.46
231	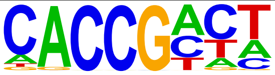	At1g75490(AP2EREBP)/colamp-At1g75490-DAP-Seq(GSE60143)/Homer	1e-14	-3.325e+01	0.0000	6547.0	18.1
232	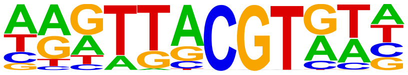	NAP(NAC)/col-NAP-DAP-Seq(GSE60143)/Homer	1e-14	-3.298e+01	0.0000	3224.0	8.93
233	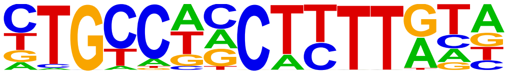	ZNF7(Zf)/HepG2-ZNF7.Flag-ChIP-Seq(Encode)/Homer	1e-13	-3.180e+01	0.0000	2195.0	6.08
234	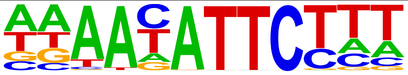	AT2G40260(G2like)/colamp-AT2G40260-DAP-Seq(GSE60143)/Homer	1e-13	-3.166e+01	0.0000	5847.0	16.1
235	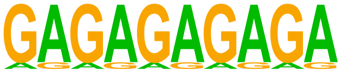	SeqBias: GA-repeat	1e-13	-3.159e+01	0.0000	23284.0	64.4
236	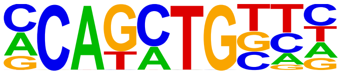	Twist2(bHLH)/Myoblast-Twist2.Ty1-ChIP-Seq(GSE127998)/Homer	1e-13	-3.157e+01	0.0000	8336.0	23.0
237	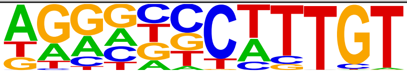	Sox9(HMG)/Limb-SOX9-ChIP-Seq(GSE73225)/Homer	1e-13	-3.149e+01	0.0000	3306.0	9.15
238	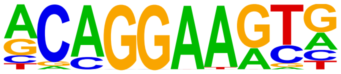	ERG(ETS)/VCaP-ERG-ChIP-Seq(GSE14097)/Homer	1e-13	-3.122e+01	0.0000	5532.0	15.3
239	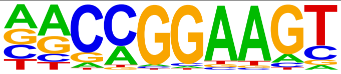	GABPA(ETS)/Jurkat-GABPa-ChIP-Seq(GSE17954)/Homer	1e-13	-3.110e+01	0.0000	2882.0	7.98
240	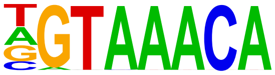	Foxo3(Forkhead)/U2OS-Foxo3-ChIP-Seq(E-MTAB-2701)/Homer	1e-13	-3.084e+01	0.0000	2780.0	7.70
241	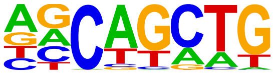	SCL(bHLH)/HPC7-Scl-ChIP-Seq(GSE13511)/Homer	1e-13	-3.049e+01	0.0000	22088.0	61.1
242	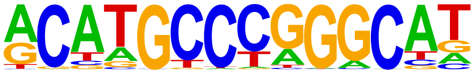	p53(p53)/mES-cMyc-ChIP-Seq(GSE11431)/Homer	1e-12	-2.982e+01	0.0000	78.0	0.22
243	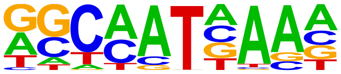	Hoxa9(Homeobox)/ChickenMSG-Hoxa9.Flag-ChIP-Seq(GSE86088)/Homer	1e-12	-2.979e+01	0.0000	11107.0	30.7
244		ERF10(AP2EREBP)/col-ERF10-DAP-Seq(GSE60143)/Homer	1e-12	-2.979e+01	0.0000	3705.0	10.2

	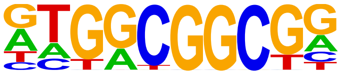						
245	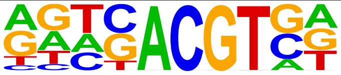	HY5(bZIP)/colamp-HY5-DAP-Seq(GSE60143)/Homer	1e-12	-2.966e+01	0.0000	2931.0	8.11
246	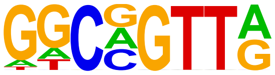	MYB(HTH)/ERMYB-Myb-ChIPSeq(GSE22095)/Homer	1e-12	-2.965e+01	0.0000	8060.0	22.3
247	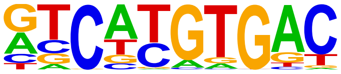	MITF(bHLH)/MastCells-MITF-ChIP-Seq(GSE48085)/Homer	1e-12	-2.932e+01	0.0000	2983.0	8.26
248	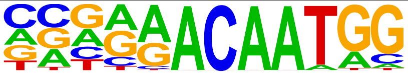	Sox7(HMG)/ESC-Sox7-ChIP-Seq(GSE133899)/Homer	1e-12	-2.924e+01	0.0000	1019.0	2.82
249	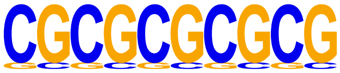	SeqBias: CG-repeat	1e-12	-2.889e+01	0.0000	7138.0	19.7
250	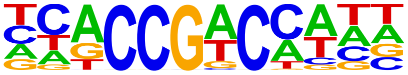	At1g19210(AP2EREBP)/colamp-At1g19210-DAP-Seq(GSE60143)/Homer	1e-12	-2.784e+01	0.0000	3520.0	9.74
251	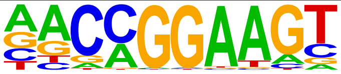	ETV1(ETS)/GIST48-ETV1-ChIP-Seq(GSE22441)/Homer	1e-12	-2.774e+01	0.0000	4490.0	12.4
252	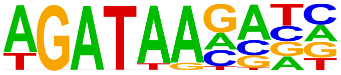	TRPS1(Zf)/MCF7-TRPS1-ChIP-Seq(GSE107013)/Homer	1e-11	-2.739e+01	0.0000	7506.0	20.7
253	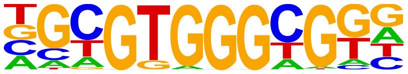	Egr2(Zf)/Thymocytes-Egr2-ChIP-Seq(GSE34254)/Homer	1e-11	-2.685e+01	0.0000	635.0	1.76
254	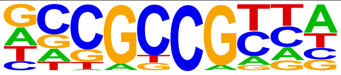	CRF10(AP2EREBP)/col100-CRF10-DAP-Seq(GSE60143)/Homer	1e-11	-2.615e+01	0.0000	6131.0	16.9
255	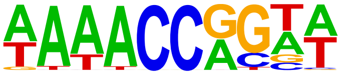	AT1G76870(Trihelix)/col-AT1G76870-DAP-Seq(GSE60143)/Homer	1e-11	-2.603e+01	0.0000	1362.0	3.77
256	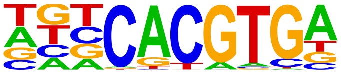	SPCH(bHLH)/Seedling-SPCH-ChIP-Seq(GSE57497)/Homer	1e-11	-2.587e+01	0.0000	4113.0	11.3
257	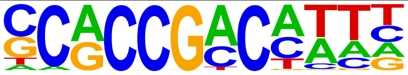	At5g65130(AP2EREBP)/colamp-At5g65130-DAP-Seq(GSE60143)/Homer	1e-11	-2.577e+01	0.0000	1111.0	3.08
258	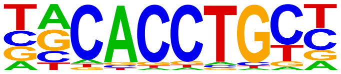	Snail1(Zf)/LS174T-SNAIL1.HA-ChIP-Seq(GSE127183)/Homer	1e-11	-2.569e+01	0.0000	3344.0	9.26
259	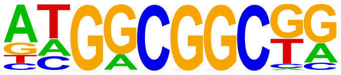	AT4G18450(AP2EREBP)/col-AT4G18450-DAP-Seq(GSE60143)/Homer	1e-11	-2.548e+01	0.0000	2817.0	7.80
260	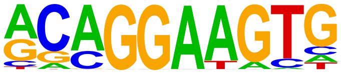	ETS1(ETS)/Jurkat-ETS1-ChIP-Seq(GSE17954)/Homer	1e-10	-2.492e+01	0.0000	3558.0	9.85
261	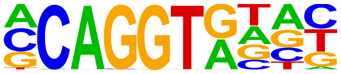	ZEB1(Zf)/PDAC-ZEB1-ChIP-Seq(GSE64557)/Homer	1e-10	-2.471e+01	0.0000	6627.0	18.3
262	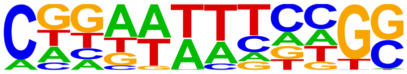	LBD18(LOBAS2)/colamp-LBD18-DAP-Seq(GSE60143)/Homer	1e-10	-2.446e+01	0.0000	9876.0	27.3
263	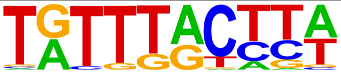	FOXM1(Forkhead)/MCF7-FOXM1-ChIP-Seq(GSE72977)/Homer	1e-10	-2.440e+01	0.0000	3986.0	11.0
264	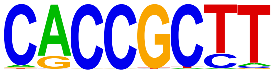	At5g18450(AP2EREBP)/col-At5g18450-DAP-Seq(GSE60143)/Homer	1e-10	-2.415e+01	0.0000	7100.0	19.6
265	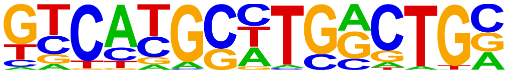	Pax8(Paired,Homeobox)/Thyroid-Pax8-ChIP-Seq(GSE26938)/Homer	1e-10	-2.409e+01	0.0000	1192.0	3.30
266	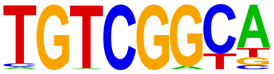	Rap210(AP2EREBP)/col-Rap210-DAP-Seq(GSE60143)/Homer	1e-10	-2.403e+01	0.0000	1534.0	4.25
267	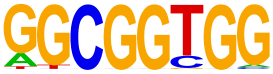	AT3G57600(AP2EREBP)/col-AT3G57600-DAP-Seq(GSE60143)/Homer	1e-10	-2.389e+01	0.0000	4413.0	12.2
268	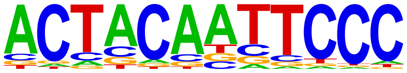	GFY(?)/Promoter/Homer	1e-10	-2.387e+01	0.0000	271.0	0.75

269	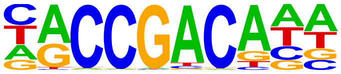	At4g16750(AP2EREBP)/col-At4g16750-DAP-Seq(GSE60143)/Homer	1e-10	-2.378e+01	0.0000	1804.0	4.99
270	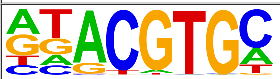	HIF-1b(HLH)/T47D-HIF1b-ChIP-Seq(GSE59937)/Homer	1e-10	-2.367e+01	0.0000	5676.0	15.7
271	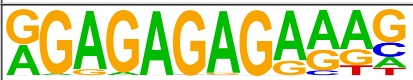	FRS9(ND)/col-FRS9-DAP-Seq(GSE60143)/Homer	1e-10	-2.343e+01	0.0000	799.0	2.21
272	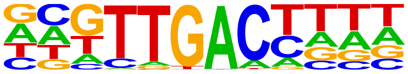	WRKY27(WRKY)/colamp-WRKY27-DAP-Seq(GSE60143)/Homer	1e-10	-2.326e+01	0.0000	2515.0	6.96
273	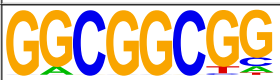	ERF104(AP2EREBP)/col-ERF104-DAP-Seq(GSE60143)/Homer	1e-9	-2.284e+01	0.0000	5303.0	14.6
274	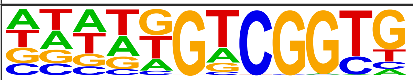	At1g36060(AP2EREBP)/colamp-At1g36060-DAP-Seq(GSE60143)/Homer	1e-9	-2.278e+01	0.0000	1811.0	5.01
275	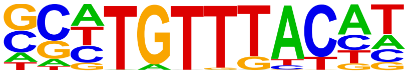	FOXK2(Forkhead)/U2OS-FOXK2-ChIP-Seq(E-MTAB-2204)/Homer	1e-9	-2.205e+01	0.0000	2374.0	6.57
276	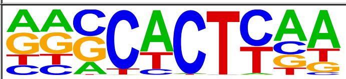	Nkx2.5(Homeobox)/HL1-Nkx2.5.biotin-ChIP-Seq(GSE21529)/Homer	1e-9	-2.178e+01	0.0000	7547.0	20.8
277	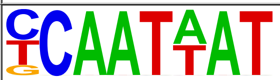	ATHB21(HB)/colamp-ATHB21-DAP-Seq(GSE60143)/Homer	1e-9	-2.173e+01	0.0000	1621.0	4.49
278	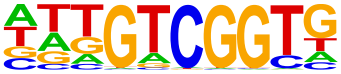	CEJ1(AP2EREBP)/col-CEJ1-DAP-Seq(GSE60143)/Homer	1e-9	-2.171e+01	0.0000	2536.0	7.02
279	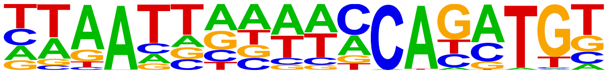	Pitx1:Ebox(Homeobox,bHLH)/Hindlimb-Pitx1-ChIP-Seq(GSE41591)/Homer	1e-9	-2.153e+01	0.0000	815.0	2.26
280	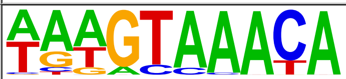	FOXA1(Forkhead)/LNCAP-FOXA1-ChIP-Seq(GSE27824)/Homer	1e-9	-2.137e+01	0.0000	4633.0	12.8
281	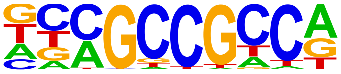	ERF5(AP2EREBP)/colamp-ERF5-DAP-Seq(GSE60143)/Homer	1e-9	-2.133e+01	0.0000	2994.0	8.29
282	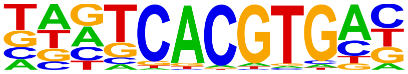	BIM3(bHLH)/col-BIM3-DAP-Seq(GSE60143)/Homer	1e-9	-2.119e+01	0.0000	466.0	1.29
283	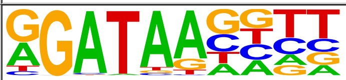	At1g49010(MYBrelated)/col-At1g49010-DAP-Seq(GSE60143)/Homer	1e-9	-2.103e+01	0.0000	6381.0	17.6
284	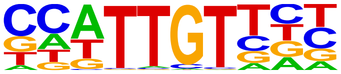	Sox6(HMG)/Myotubes-Sox6-ChIP-Seq(GSE32627)/Homer	1e-9	-2.092e+01	0.0000	6345.0	17.5
285	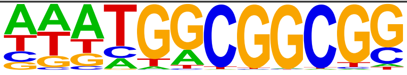	ERF9(AP2EREBP)/colamp-ERF9-DAP-Seq(GSE60143)/Homer	1e-9	-2.092e+01	0.0000	2014.0	5.58
286	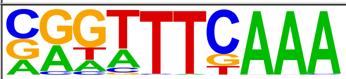	CHR(?)/Hela-CellCycle-Expression/Homer	1e-8	-2.033e+01	0.0000	2240.0	6.20
287	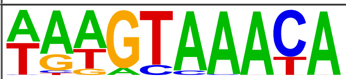	FOXA1(Forkhead)/MCF7-FOXA1-ChIP-Seq(GSE26831)/Homer	1e-8	-1.985e+01	0.0000	3776.0	10.4
288	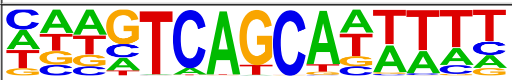	MafF(bZIP)/HepG2-MafF-ChIP-Seq(GSE31477)/Homer	1e-8	-1.956e+01	0.0000	1359.0	3.76
289	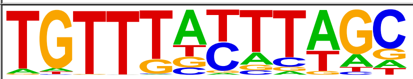	FoxD3(forkhead)/ZebrafishEmbryo-Foxd3.biotin-ChIP-seq(GSE106676)/Homer	1e-8	-1.954e+01	0.0000	3445.0	9.54
290	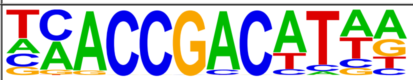	DREB2(AP2EREBP)/col-DREB2-DAP-Seq(GSE60143)/Homer	1e-8	-1.953e+01	0.0000	1165.0	3.23
291	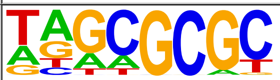	DPL-1(E2F)/cElegans-Adult-ChIP-Seq(modEncode)/Homer	1e-8	-1.909e+01	0.0000	2930.0	8.11
292	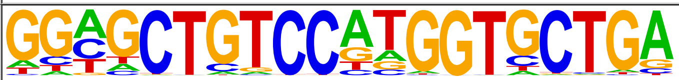	REST-NRSF(Zf)/Jurkat-NRSF-ChIP-Seq/Homer	1e-8	-1.891e+01	0.0000	58.0	0.16
293	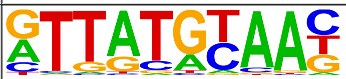	HLF(bZIP)/HSC-HLF.Flag-ChIP-Seq(GSE69817)/Homer	1e-8	-1.886e+01	0.0000	3142.0	8.70

294	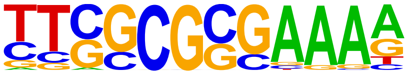	E2F(E2F)/Hela-CellCycle-Expression/Homer	1e-8	-1.877e+01	0.0000	145.0	0.40
295	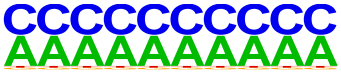	SeqBias: C/A-bias	1e-8	-1.867e+01	0.0000	36108.0	99.9
296	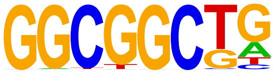	AT5G23930(mTERF)/col-AT5G23930-DAP-Seq(GSE60143)/Homer	1e-8	-1.848e+01	0.0000	8482.0	23.4
297	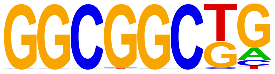	ERF2(AP2EREBP)/colamp-ERF2-DAP-Seq(GSE60143)/Homer	1e-7	-1.840e+01	0.0000	4254.0	11.7
298	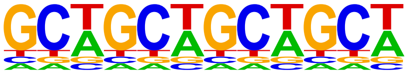	SeqBias: GCW-triplet	1e-7	-1.839e+01	0.0000	36122.0	100.
299	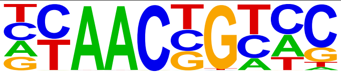	MYB77(MYB)/col-MYB77-DAP-Seq(GSE60143)/Homer	1e-7	-1.836e+01	0.0000	5691.0	15.7
300	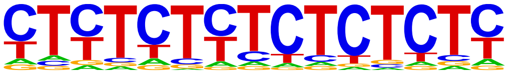	GAGA-repeat/SacCer-Promoters/Homer	1e-7	-1.821e+01	0.0000	14418.0	39.9
301	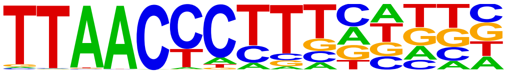	ZNF652/HepG2-ZNF652.Flag-ChIP-Seq(Encode)/Homer	1e-7	-1.820e+01	0.0000	890.0	2.46
302	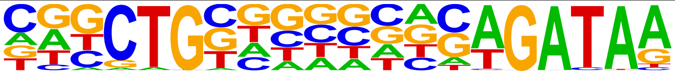	GATA:SCL(Zf,bHLH)/Ter119-SCL-ChIP-Seq(GSE18720)/Homer	1e-7	-1.807e+01	0.0000	509.0	1.41
303	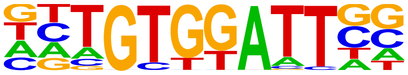	Foxh1(Forkhead)/hESC-FOXH1-ChIP-Seq(GSE29422)/Homer	1e-7	-1.799e+01	0.0000	2098.0	5.81
304	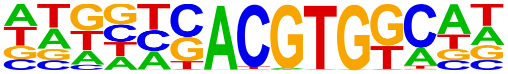	bZIP3(bZIP)/col-bZIP3-DAP-Seq(GSE60143)/Homer	1e-7	-1.780e+01	0.0000	1693.0	4.69
305	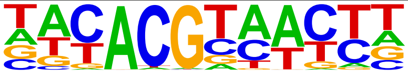	ANAC047(NAC)/colamp-ANAC047-DAP-Seq(GSE60143)/Homer	1e-7	-1.773e+01	0.0000	2428.0	6.72
306	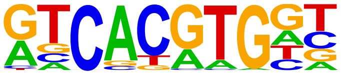	Usf2(bHLH)/C2C12-Usf2-ChIP-Seq(GSE36030)/Homer	1e-7	-1.723e+01	0.0000	1019.0	2.82
307	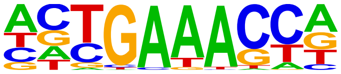	IRF4(IRF)/GM12878-IRF4-ChIP-Seq(GSE32465)/Homer	1e-7	-1.698e+01	0.0000	1811.0	5.01
308	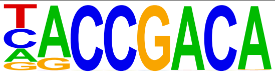	bHLH10(bHLH)/colamp-bHLH10-DAP-Seq(GSE60143)/Homer	1e-7	-1.691e+01	0.0000	787.0	2.18
309	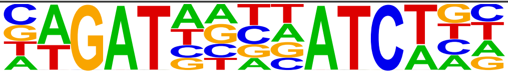	GATA(Zf),IR4/iTreg-Gata3-ChIP-Seq(GSE20898)/Homer	1e-7	-1.685e+01	0.0000	370.0	1.02
310	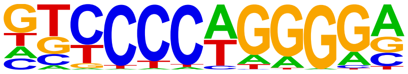	EBF1(EBF)/Near-E2A-ChIP-Seq(GSE21512)/Homer	1e-7	-1.656e+01	0.0000	2998.0	8.30
311	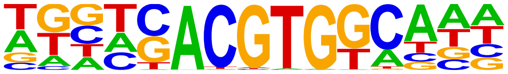	GBF5(bZIP)/colamp-GBF5-DAP-Seq(GSE60143)/Homer	1e-7	-1.639e+01	0.0000	1073.0	2.97
312	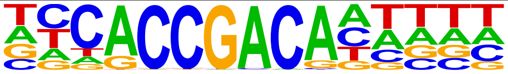	At4g32800(AP2EREBP)/colamp-At4g32800-DAP-Seq(GSE60143)/Homer	1e-7	-1.631e+01	0.0000	304.0	0.84
313	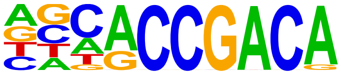	TINY(AP2EREBP)/col-TINY-DAP-Seq(GSE60143)/Homer	1e-7	-1.625e+01	0.0000	732.0	2.03
314	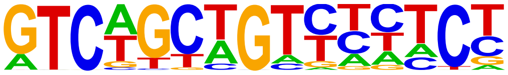	ZNF317(Zf)/HEK293-ZNF317.GFP-ChIP-Seq(GSE58341)/Homer	1e-7	-1.617e+01	0.0000	410.0	1.14
315	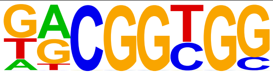	ESE3(AP2EREBP)/col-ESE3-DAP-Seq(GSE60143)/Homer	1e-6	-1.596e+01	0.0000	6182.0	17.1
316	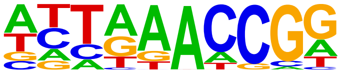	Unknown3/Arabidopsis-Promoters/Homer	1e-6	-1.587e+01	0.0000	467.0	1.29
317	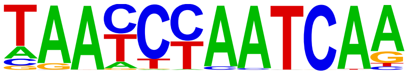	Duxbl(Homeobox)/NIH3T3-Duxbl.HA-ChIP-Seq(GSE119782)/Homer	1e-6	-1.584e+01	0.0000	225.0	0.62
318	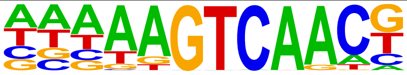	WRKY22(WRKY)/colamp-WRKY22-DAP-Seq(GSE60143)/Homer	1e-6	-1.575e+01	0.0000	1674.0	4.63

319	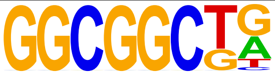	ERF1(AP2EREBP)/colamp-ERF1-DAP-Seq(GSE60143)/Homer	1e-6	-1.554e+01	0.0000	3835.0	10.6	
320	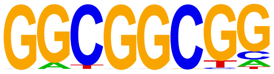	ESE1(AP2EREBP)/col-ESE1-DAP-Seq(GSE60143)/Homer	1e-6	-1.552e+01	0.0000	4504.0	12.4	
321	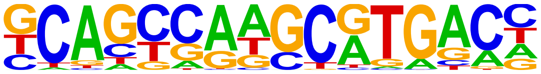	PAX5(Paired,Homeobox)/GM12878-PAX5-ChIP-Seq(GSE32465)/Homer	1e-6	-1.495e+01	0.0000	1565.0	4.33	
322	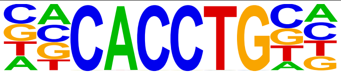	E2A(bHLH),near_PU.1/Bcell-PU.1-ChIP-Seq(GSE21512)/Homer	1e-6	-1.488e+01	0.0000	5271.0	14.5	
323	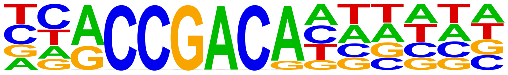	At2g44940(AP2EREBP)/colamp-At2g44940-DAP-Seq(GSE60143)/Homer	1e-6	-1.465e+01	0.0000	548.0	1.52	
324	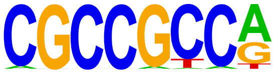	CRF4(AP2EREBP)/colamp-CRF4-DAP-Seq(GSE60143)/Homer	1e-6	-1.420e+01	0.0000	3399.0	9.41	
325	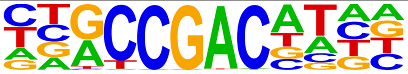	CBF3(AP2EREBP)/colamp-CBF3-DAP-Seq(GSE60143)/Homer	1e-6	-1.405e+01	0.0000	994.0	2.75	
326	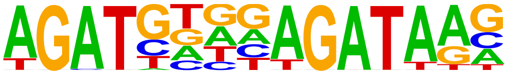	GATA3(Zf),DR4/iTreg-Gata3-ChIP-Seq(GSE20898)/Homer	1e-6	-1.400e+01	0.0000	325.0	0.90	
327	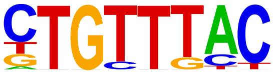	Foxo1(Forkhead)/RAW-Foxo1-ChIP-Seq(Fan_et_al.)/Homer	1e-6	-1.400e+01	0.0000	7248.0	20.0	
328	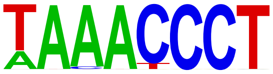	TRP2(MYBrelated)/colamp-TRP2-DAP-Seq(GSE60143)/Homer	1e-6	-1.398e+01	0.0000	553.0	1.53	
329	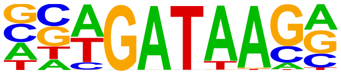	Gata4(Zf)/Heart-Gata4-ChIP-Seq(GSE35151)/Homer	1e-5	-1.351e+01	0.0000	3758.0	10.4	
330	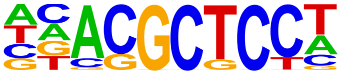	MYB88(MYB)/col-MYB88-DAP-Seq(GSE60143)/Homer	1e-5	-1.345e+01	0.0000	7849.0	21.7	
331	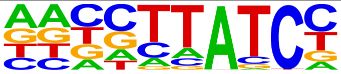	At5g08520(MYBrelated)/colamp-At5g08520-DAP-Seq(GSE60143)/Homer	1e-5	-1.336e+01	0.0000	3506.0	9.71	
332	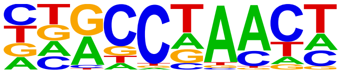	BOS1(MYB)/col-BOS1-DAP-Seq(GSE60143)/Homer	1e-5	-1.316e+01	0.0000	2710.0	7.50	
333	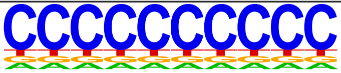	SeqBias: polyC-repeat	1e-5	-1.308e+01	0.0000	35785.0	99.0	
334	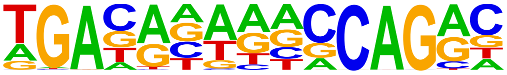	Hand2(bHLH)/Mesoderm-Hand2-ChIP-Seq(GSE61475)/Homer	1e-5	-1.299e+01	0.0000	1509.0	4.18	
335	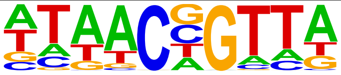	MYB70(MYB)/col-MYB70-DAP-Seq(GSE60143)/Homer	1e-5	-1.261e+01	0.0000	6407.0	17.7	
336	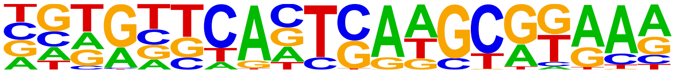	PAX6(Paired,Homeobox)/Forebrain-Pax6-ChIP-Seq(GSE66961)/Homer	1e-5	-1.260e+01	0.0000	378.0	1.05	
337	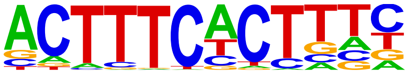	PRDM1(Zf)/Hela-PRDM1-ChIP-Seq(GSE31477)/Homer	1e-5	-1.258e+01	0.0000	1817.0	5.03	
338	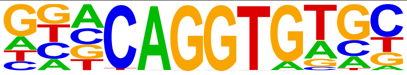	ZEB2(Zf)/SNU398-ZEB2-ChIP-Seq(GSE103048)/Homer	1e-5	-1.242e+01	0.0000	3343.0	9.25	
339	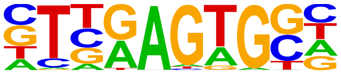	Nkx2.2(Homeobox)/NPC-Nkx2.2-ChIP-Seq(GSE61673)/Homer	1e-5	-1.230e+01	0.0000	6672.0	18.4	
340	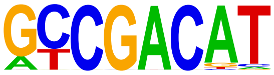	DDF1(AP2EREBP)/col-DDF1-DAP-Seq(GSE60143)/Homer	1e-5	-1.229e+01	0.0000	905.0	2.51	
341	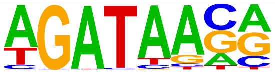	GATA3(Zf)/iTreg-Gata3-ChIP-Seq(GSE20898)/Homer	1e-5	-1.216e+01	0.0000	5440.0	15.0	
342	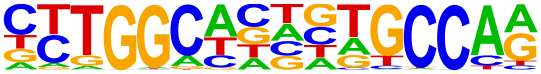	NF1(CTF)/LNCAP-NF1-ChIP-Seq(Unpublished)/Homer	1e-5	-1.211e+01	0.0000	1114.0	3.08	
343		AT5G61620(MYBrelated)/colamp-AT5G61620-DAP-	1e-5	-1.200e+01	0.0000	4594.0	12.7	

	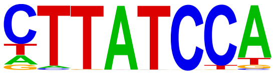	Seq(GSE60143)/Homer					
344	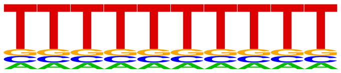	VRN1(ABI3VP1)/col-VRN1-DAP-Seq(GSE60143)/Homer	1e-5	-1.199e+01	0.0000	993.0	2.75
345	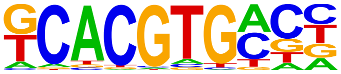	bHLHE41(bHLH)/proB-Bhlhe41-ChIP-Seq(GSE93764)/Homer	1e-5	-1.192e+01	0.0000	4640.0	12.8
346	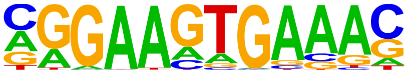	PU.1-IRF(ETS:IRF)/Bcell-PU.1-ChIP-Seq(GSE21512)/Homer	1e-5	-1.181e+01	0.0000	4780.0	13.2
347	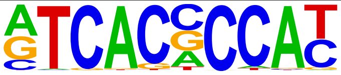	Srebp1a(bHLH)/HepG2-Srebp1a-ChIP-Seq(GSE31477)/Homer	1e-5	-1.171e+01	0.0000	684.0	1.89
348	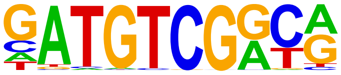	DDF2(AP2EREBP)/col-DDF2-DAP-Seq(GSE60143)/Homer	1e-5	-1.159e+01	0.0000	116.0	0.32
349	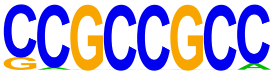	ERF73(AP2EREBP)/col-ERF73-DAP-Seq(GSE60143)/Homer	1e-4	-1.151e+01	0.0000	4260.0	11.7
350	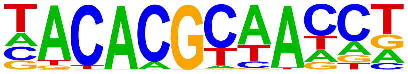	ANAC079(NAC)/colamp-ANAC079-DAP-Seq(GSE60143)/Homer	1e-4	-1.139e+01	0.0000	1481.0	4.10
351	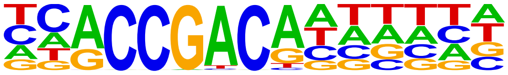	AT3G16280(AP2EREBP)/colamp-AT3G16280-DAP-Seq(GSE60143)/Homer	1e-4	-1.109e+01	0.0000	848.0	2.35
352	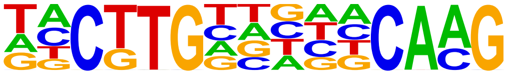	ANAC017(NAC)/colamp-ANAC017-DAP-Seq(GSE60143)/Homer	1e-4	-1.108e+01	0.0000	297.0	0.82
353	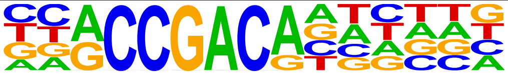	AT3G60490(AP2EREBP)/colamp-AT3G60490-DAP-Seq(GSE60143)/Homer	1e-4	-1.100e+01	0.0000	754.0	2.09
354	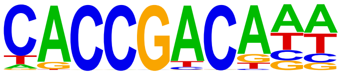	At4g31060(AP2EREBP)/colamp-At4g31060-DAP-Seq(GSE60143)/Homer	1e-4	-1.083e+01	0.0001	1020.0	2.82
355	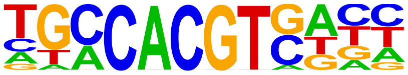	bZIP28(bZIP)/col-bZIP28-DAP-Seq(GSE60143)/Homer	1e-4	-1.079e+01	0.0001	927.0	2.57
356	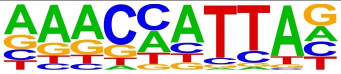	Barx1(Homeobox)/Stomach-Barx1.3xFlag-ChIP-Seq(GSE69483)/Homer	1e-4	-1.077e+01	0.0001	1917.0	5.31
357	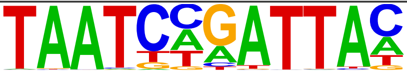	Pax7(Paired,Homeobox),long/Myoblast-Pax7-ChIP-Seq(GSE25064)/Homer	1e-4	-1.071e+01	0.0001	114.0	0.32
358	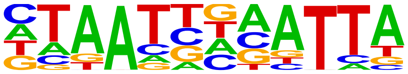	Prop1(Homeobox)/GHFT1-PROP1.biotin-ChIP-Seq(GSE77302)/Homer	1e-4	-1.035e+01	0.0001	2230.0	6.17
359	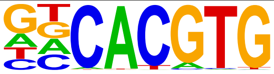	BMAL1(bHLH)/Liver-Bmal1-ChIP-Seq(GSE39860)/Homer	1e-4	-1.032e+01	0.0001	6295.0	17.4
360	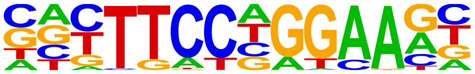	Stat3+il21(Stat)/CD4-Stat3-ChIP-Seq(GSE19198)/Homer	1e-4	-1.029e+01	0.0001	2274.0	6.30
361	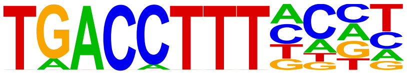	Nur77(NR)/K562-NR4A1-ChIP-Seq(GSE31363)/Homer	1e-4	-1.021e+01	0.0001	640.0	1.77
362	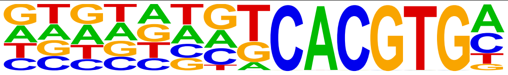	BIM1(bHLH)/colamp-BIM1-DAP-Seq(GSE60143)/Homer	1e-4	-1.017e+01	0.0001	429.0	1.19
363	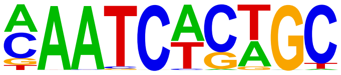	Gfi1b(Zf)/HPC7-Gfi1b-ChIP-Seq(GSE22178)/Homer	1e-4	-1.012e+01	0.0001	3180.0	8.80
364	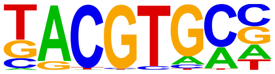	HIF-1a(bHLH)/MCF7-HIF1a-ChIP-Seq(GSE28352)/Homer	1e-4	-1.007e+01	0.0001	1358.0	3.76
365	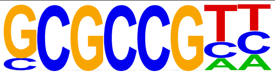	PUCHI(AP2EREBP)/colamp-PUCHI-DAP-Seq(GSE60143)/Homer	1e-4	-1.001e+01	0.0001	4467.0	12.3
366	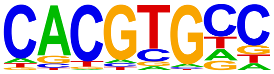	IBL1(bHLH)/Seedling-IBL1-ChIP-Seq(GSE51120)/Homer	1e-4	-9.901e+00	0.0001	6743.0	18.6
367		WRKY7(WRKY)/colamp-WRKY7-	1e-4	-9.805e+00	0.0002	10.0	0.03

	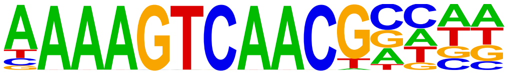	DAP-Seq(GSE60143)/Homer					
368	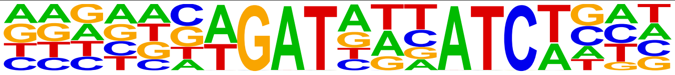	GATA(Zf),IR3/iTreg-Gata3-ChIP-Seq(GSE20898)/Homer	1e-4	-9.801e+00	0.0002	569.0	1.58
369	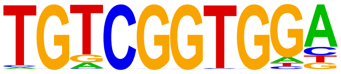	At1g77640(AP2EREBP)/col-At1g77640-DAP-Seq(GSE60143)/Homer	1e-4	-9.636e+00	0.0002	537.0	1.49
370	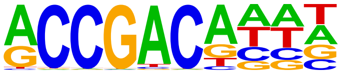	AT1G77200(AP2EREBP)/colamp-AT1G77200-DAP-Seq(GSE60143)/Homer	1e-4	-9.283e+00	0.0003	1753.0	4.85
371	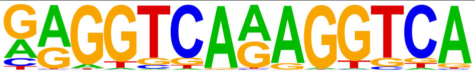	TR4(NR),DR1/Hela-TR4-ChIP-Seq(GSE24685)/Homer	1e-4	-9.259e+00	0.0003	238.0	0.66
372	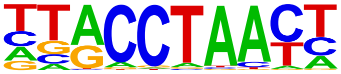	MYB57(MYB)/col-MYB57-DAP-Seq(GSE60143)/Homer	1e-3	-9.201e+00	0.0003	1229.0	3.40
373	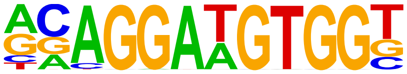	ETS:RUNX(ETS,Runt)/Jurkat-RUNX1-ChIP-Seq(GSE17954)/Homer	1e-3	-9.111e+00	0.0003	227.0	0.63
374	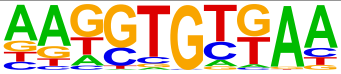	Tbr1(T-box)/Cortex-Tbr1-ChIP-Seq(GSE71384)/Homer	1e-3	-9.008e+00	0.0003	5362.0	14.8
375	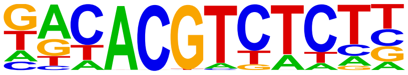	bHLH157(bHLH)/col-bHLH157-DAP-Seq(GSE60143)/Homer	1e-3	-8.974e+00	0.0003	875.0	2.42
376	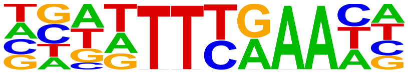	TCX2(CPP)/colamp-TCX2-DAP-Seq(GSE60143)/Homer	1e-3	-8.768e+00	0.0004	5110.0	14.1
377	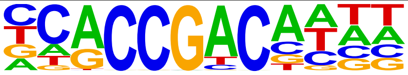	RAP21(AP2EREBP)/colamp-RAP21-DAP-Seq(GSE60143)/Homer	1e-3	-8.722e+00	0.0004	414.0	1.15
378	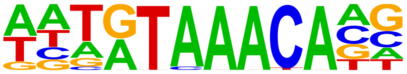	FoxL2(Forkhead)/Ovary-FoxL2-ChIP-Seq(GSE60858)/Homer	1e-3	-8.701e+00	0.0004	2893.0	8.01
379	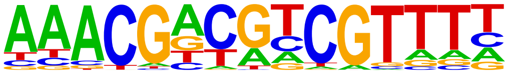	Unknown2/Arabidopsis-Promoters/Homer	1e-3	-8.691e+00	0.0004	19.0	0.05
380	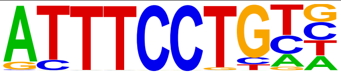	EWS:ERG-fusion(ETS)/CADO_ES1-EWS:ERG-ChIP-Seq(SRA014231)/Homer	1e-3	-8.638e+00	0.0005	2325.0	6.44
381	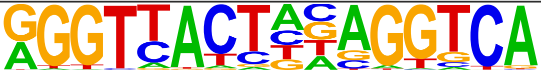	LXRE(NR),DR4/RAW-LXRb.biotin-ChIP-Seq(GSE21512)/Homer	1e-3	-8.600e+00	0.0005	132.0	0.37
382	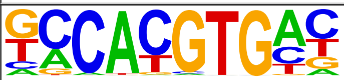	NPAS2(bHLH)/Liver-NPAS2-ChIP-Seq(GSE39860)/Homer	1e-3	-8.586e+00	0.0005	3756.0	10.4
383	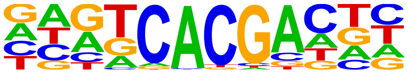	Npas4(bHLH)/Neuron-Npas4-ChIP-Seq(GSE127793)/Homer	1e-3	-8.301e+00	0.0007	3558.0	9.85
384	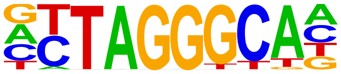	TBP3(MYBrelated)/col-TBP3-DAP-Seq(GSE60143)/Homer	1e-3	-8.247e+00	0.0007	1808.0	5.01
385	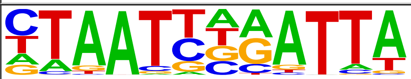	Phox2a(Homeobox)/Neuron-Phox2a-ChIP-Seq(GSE31456)/Homer	1e-3	-8.028e+00	0.0009	1348.0	3.73
386	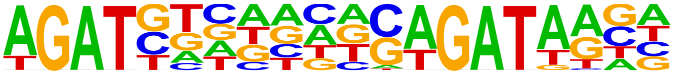	GATA3(Zf),DR8/iTreg-Gata3-ChIP-Seq(GSE20898)/Homer	1e-3	-8.002e+00	0.0009	306.0	0.85
387	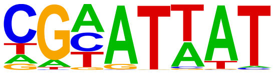	AtHB32(ZFHD)/col200-AtHB32-DAP-Seq(GSE60143)/Homer	1e-3	-7.793e+00	0.0011	4320.0	11.9
388	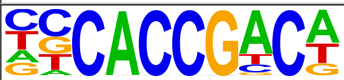	AT1G44830(AP2EREBP)/col-AT1G44830-DAP-Seq(GSE60143)/Homer	1e-3	-7.662e+00	0.0012	1019.0	2.82
389	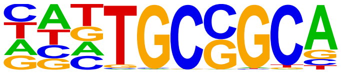	Zfp57(Zf)/H1-ZFP57.HA-ChIP-Seq(GSE115387)/Homer	1e-3	-7.645e+00	0.0012	2185.0	6.05
390	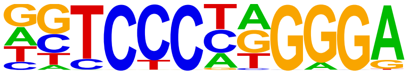	EBF(EBF)/proBcell-EBF-ChIP-Seq(GSE21978)/Homer	1e-3	-7.576e+00	0.0013	537.0	1.49
391		CBF2(AP2EREBP)/colamp-CBF2-DAP-	1e-3	-7.509e+00	0.0014	965.0	2.67

	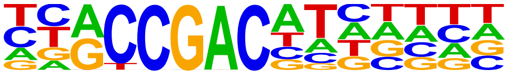	Seq(GSE60143)/Homer					
392	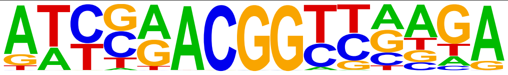	ZIM(C2C2gata)/col-ZIM-DAP-Seq(GSE60143)/Homer	1e-3	-7.398e+00	0.0016	27.0	0.07
393	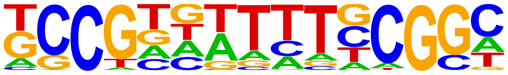	LBD13(LOBAS2)/colamp-LBD13-DAP-Seq(GSE60143)/Homer	1e-3	-7.375e+00	0.0016	3036.0	8.40
394	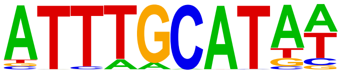	Oct4(POU,Homeobox)/mES-Oct4-ChIP-Seq(GSE11431)/Homer	1e-3	-7.335e+00	0.0017	1786.0	4.94
395	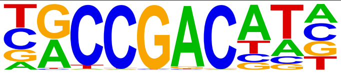	CBF1(AP2EREBP)/colamp-CBF1-DAP-Seq(GSE60143)/Homer	1e-3	-7.320e+00	0.0017	1491.0	4.13
396	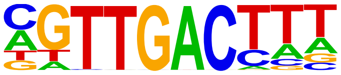	WRKY29(WRKY)/colamp-WRKY29-DAP-Seq(GSE60143)/Homer	1e-3	-7.267e+00	0.0018	2620.0	7.25
397	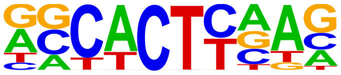	Nkx2.1(Homeobox)/LungAC-Nkx2.1-ChIP-Seq(GSE43252)/Homer	1e-3	-7.216e+00	0.0019	9936.0	27.5
398	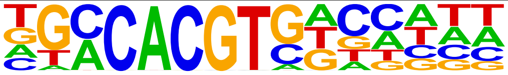	ABF2(bZIP)/col-ABF2-DAP-Seq(GSE60143)/Homer	1e-3	-7.132e+00	0.0020	717.0	1.98
399	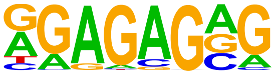	Trl(Zf)/S2-GAGAfactor-ChIP-Seq(GSE40646)/Homer	1e-3	-7.094e+00	0.0021	10036.0	27.7
400	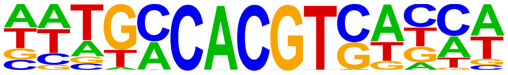	GBF6(bZIP)/colamp-GBF6-DAP-Seq(GSE60143)/Homer	1e-3	-7.058e+00	0.0022	737.0	2.04
401	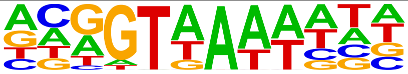	GT2(Trihelix)/colamp-GT2-DAP-Seq(GSE60143)/Homer	1e-3	-7.041e+00	0.0022	3891.0	10.7
402	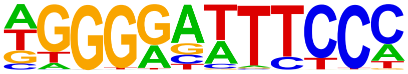	NFkB-p65(RHD)/GM12787-p65-ChIP-Seq(GSE19485)/Homer	1e-3	-6.943e+00	0.0024	1420.0	3.93
403	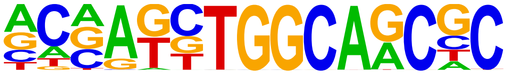	Unknown3/Drosophila-Promoters/Homer	1e-2	-6.900e+00	0.0025	446.0	1.23
404	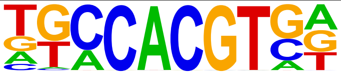	bZIP16(bZIP)/colamp-bZIP16-DAP-Seq(GSE60143)/Homer	1e-2	-6.894e+00	0.0025	921.0	2.55
405	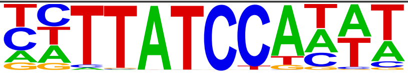	At1g74840(MYBrelated)/col100-At1g74840-DAP-Seq(GSE60143)/Homer	1e-2	-6.881e+00	0.0026	2273.0	6.29
406	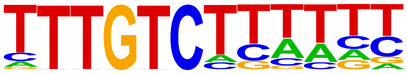	SGR5(C2H2)/colamp-SGR5-DAP-Seq(GSE60143)/Homer	1e-2	-6.813e+00	0.0027	2732.0	7.56
407	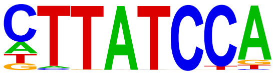	At5g47390(MYBrelated)/col-At5g47390-DAP-Seq(GSE60143)/Homer	1e-2	-6.772e+00	0.0028	3952.0	10.9
408	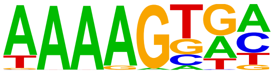	CDF3(C2C2dof)/colamp-CDF3-DAP-Seq(GSE60143)/Homer	1e-2	-6.710e+00	0.0030	6895.0	19.0
409	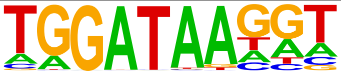	AT5G56840(MYBrelated)/colamp-AT5G56840-DAP-Seq(GSE60143)/Homer	1e-2	-6.652e+00	0.0032	4003.0	11.0
410	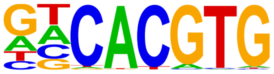	CLOCK(bHLH)/Liver-Clock-ChIP-Seq(GSE39860)/Homer	1e-2	-6.631e+00	0.0032	1939.0	5.37
411	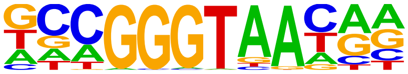	REB1/SacCer-Promoters/Homer	1e-2	-6.606e+00	0.0033	523.0	1.45
412	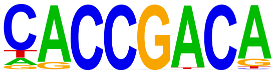	ERF38(AP2EREBP)/col-ERF38-DAP-Seq(GSE60143)/Homer	1e-2	-6.531e+00	0.0036	1237.0	3.42
413	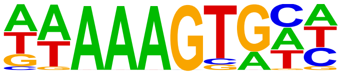	AT1G69570(C2C2dof)/col-AT1G69570-DAP-Seq(GSE60143)/Homer	1e-2	-6.512e+00	0.0036	4685.0	12.9
414	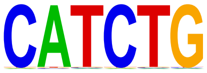	Tal1	1e-2	-6.472e+00	0.0038	5895.0	16.3
415	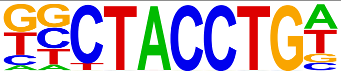	Zelda(Zf)/Embryo-zld-ChIP-Seq(GSE65441)/Homer	1e-2	-6.418e+00	0.0040	1664.0	4.61

416	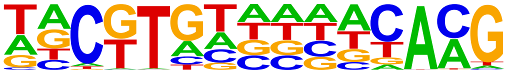	CUC2(NAC)/colamp-CUC2-DAP-Seq(GSE60143)/Homer	1e-2	-6.300e+00	0.0044	936.0	2.59
417	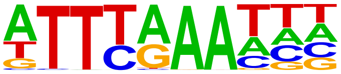	SOL1(CPP)/colamp-SOL1-DAP-Seq(GSE60143)/Homer	1e-2	-6.206e+00	0.0049	4924.0	13.6
418	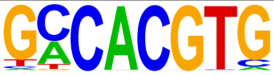	E-box/Arabidopsis-Promoters/Homer	1e-2	-6.130e+00	0.0052	1767.0	4.89
419	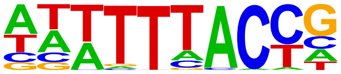	AT5G47660(Trihelix)/colamp-AT5G47660-DAP-Seq(GSE60143)/Homer	1e-2	-6.038e+00	0.0057	5184.0	14.3
420	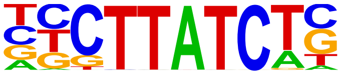	Gata2(Zf)/K562-GATA2-ChIP-Seq(GSE18829)/Homer	1e-2	-6.028e+00	0.0058	2347.0	6.50
421	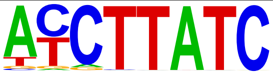	At5g05790(MYBrelated)/col-At5g05790-DAP-Seq(GSE60143)/Homer	1e-2	-5.994e+00	0.0060	2964.0	8.21
422	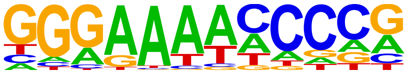	Dorsal(RHD)/Embryo-dl-ChIP-Seq(GSE65441)/Homer	1e-2	-5.993e+00	0.0060	626.0	1.73
423	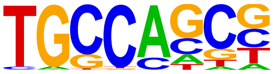	HIC1(Zf)/Treg-ZBTB29-ChIP-Seq(GSE99889)/Homer	1e-2	-5.925e+00	0.0064	8804.0	24.3
424	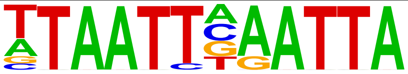	Phox2b(Homeobox)/CLBGA-PHOX2B-ChIP-Seq(GSE90683)/Homer	1e-2	-5.915e+00	0.0064	675.0	1.87
425	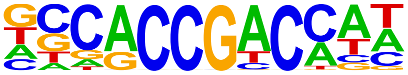	At4g28140(AP2EREBP)/colamp-At4g28140-DAP-Seq(GSE60143)/Homer	1e-2	-5.819e+00	0.0070	909.0	2.52
426	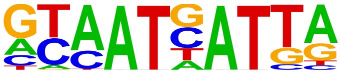	ATHB15(HB)/col-ATHB15-DAP-Seq(GSE60143)/Homer	1e-2	-5.817e+00	0.0070	1005.0	2.78
427	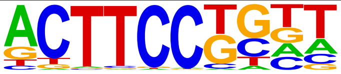	Elf4(ETS)/BMDM-Elf4-ChIP-Seq(GSE88699)/Homer	1e-2	-5.809e+00	0.0071	2987.0	8.27
428	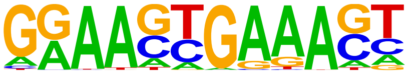	IRF8(IRF)/BMDM-IRF8-ChIP-Seq(GSE77884)/Homer	1e-2	-5.770e+00	0.0073	996.0	2.76
429	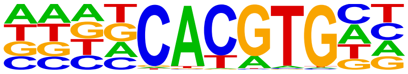	BIM2(bHLH)/col-BIM2-DAP-Seq(GSE60143)/Homer	1e-2	-5.717e+00	0.0077	3119.0	8.63
430	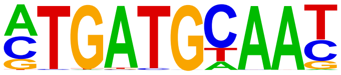	Atf4(bZIP)/MEF-Atf4-ChIP-Seq(GSE35681)/Homer	1e-2	-5.702e+00	0.0078	1299.0	3.60
431	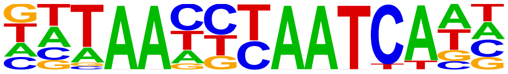	DUX4(Homeobox)/Myoblasts-DUX4.V5-ChIP-Seq(GSE75791)/Homer	1e-2	-5.689e+00	0.0079	110.0	0.30
432	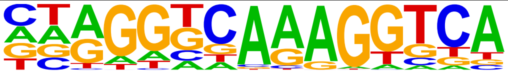	PPARa(NR),DR1/Liver-Ppara-ChIP-Seq(GSE47954)/Homer	1e-2	-5.685e+00	0.0079	2776.0	7.69
433	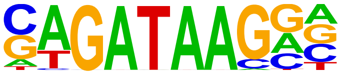	Gata1(Zf)/K562-GATA1-ChIP-Seq(GSE18829)/Homer	1e-2	-5.666e+00	0.0080	2114.0	5.85
434	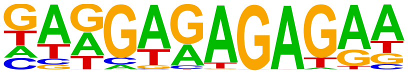	BPC1(BBRBPC)/colamp-BPC1-DAP-Seq(GSE60143)/Homer	1e-2	-5.602e+00	0.0086	1902.0	5.27
435	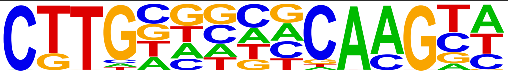	AT1G19040(NAC)/col-AT1G19040-DAP-Seq(GSE60143)/Homer	1e-2	-5.394e+00	0.0105	285.0	0.79
436	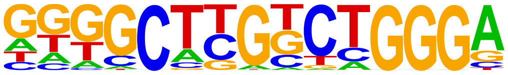	Zfp809(Zf)/ES-Zfp809-ChIP-Seq(GSE70799)/Homer	1e-2	-5.390e+00	0.0105	736.0	2.04
437	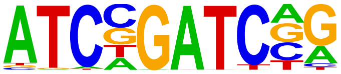	GATA19(C2C2gata)/colamp-GATA19-DAP-Seq(GSE60143)/Homer	1e-2	-5.277e+00	0.0118	260.0	0.72
438	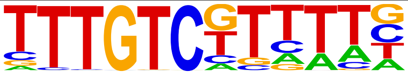	IDD7(C2H2)/col-IDD7-DAP-Seq(GSE60143)/Homer	1e-2	-5.238e+00	0.0122	1455.0	4.03
439	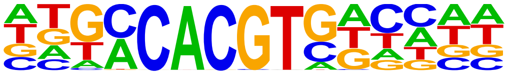	AREB3(bZIP)/col-AREB3-DAP-Seq(GSE60143)/Homer	1e-2	-5.176e+00	0.0130	993.0	2.75
440	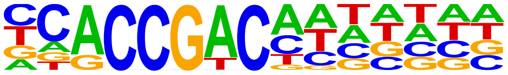	DEAR3(AP2EREBP)/colamp-DEAR3-DAP-Seq(GSE60143)/Homer	1e-2	-5.104e+00	0.0139	697.0	1.93

441	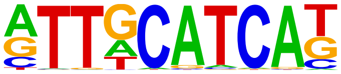	Chop(bZIP)/MEF-Chop-ChIP-Seq(GSE35681)/Homer	1e-2	-5.065e+00	0.0144	978.0	2.71
442	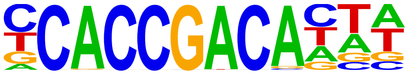	AT1G01250(AP2EREBP)/col-AT1G01250-DAP-Seq(GSE60143)/Homer	1e-2	-5.064e+00	0.0144	230.0	0.64
443	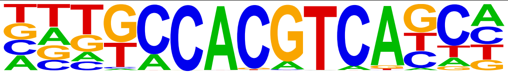	bZIP44(bZIP)/colamp-bZIP44-DAP-Seq(GSE60143)/Homer	1e-2	-5.007e+00	0.0152	89.0	0.25
444	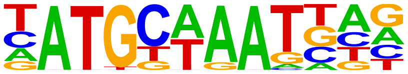	Brn1(POU,Homeobox)/NPC-Brn1-ChIP-Seq(GSE35496)/Homer	1e-2	-4.991e+00	0.0154	1097.0	3.04
445	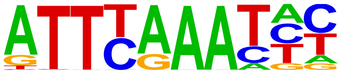	AT2G20110(CPP)/colamp-AT2G20110-DAP-Seq(GSE60143)/Homer	1e-2	-4.973e+00	0.0157	4864.0	13.4
446	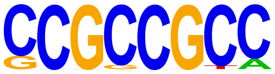	SHN3(AP2EREBP)/col-SHN3-DAP-Seq(GSE60143)/Homer	1e-2	-4.919e+00	0.0165	2844.0	7.87
447	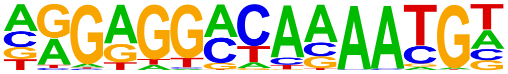	ZNF675(Zf)/HEK293-ZNF675.GFP-ChIP-Seq(GSE58341)/Homer	1e-2	-4.737e+00	0.0197	425.0	1.18
448	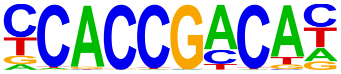	DREB26(AP2EREBP)/col-DREB26-DAP-Seq(GSE60143)/Homer	1e-2	-4.713e+00	0.0202	784.0	2.17
449	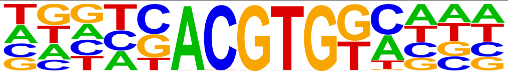	bZIP53(bZIP)/colamp-bZIP53-DAP-Seq(GSE60143)/Homer	1e-2	-4.686e+00	0.0207	1031.0	2.85
450	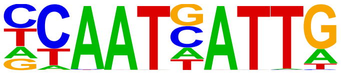	ATHB18(Homeobox)/colamp-ATHB18-DAP-Seq(GSE60143)/Homer	1e-2	-4.681e+00	0.0207	541.0	1.50
451	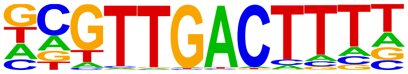	WRKY21(WRKY)/colamp-WRKY21-DAP-Seq(GSE60143)/Homer	1e-2	-4.672e+00	0.0209	250.0	0.69
