# Supplementary material for: PBX1 and PBX3 transcription factors regulate SHH expression in the Frontonasal Ectodermal Zone through complementary mechanisms
Source: PLoS Genet. 2025 May 21;21(5):e1011315. doi: 10.1371/journal.pgen.1011315 (PMC12140432; doi:10.1371/journal.pgen.1011315)
Supplement: S6 Table — (PDF) [file pgen.1011315.s012.pdf]

S6 Table. Full list of de novo motif discovery from ChIP-seq data targeting PBX3.

Homer *de novo* Motif Results (/wynton/group/marcucio/2022CHM/Data2022Mar/Motif/HomerPBX3IDR/)

Known Motif Enrichment Results
Gene Ontology Enrichment Results
If Homer is having trouble matching a motif to a known motif, try copy/pasting the matrix file into STAMP
More information on motif finding results: HOMER | Description of Results | Tips
Total target sequences = 36123
Total background sequences = 35367
* - possible false positive

Rank	Motif	P-value	log P-pvalue	% of Targets	% of Background	STD(Bg STD)	Best Match/Details	Motif File
1	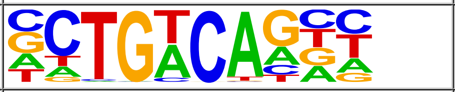	1e-3291	-7.579e+03	49.34%	20.25%	49.3bp (64.6bp)	Meis1(Homeobox)/MastCells-Meis1-ChIP-Seq(GSE48085)/Homer(0.962) More Information | Similar Motifs Found	motif file (matrix)
2	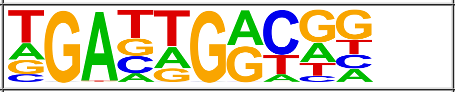	1e-1664	-3.832e+03	18.73%	5.54%	51.6bp (62.7bp)	Pknox1(Homeobox)/ES-Prep1-ChIP-Seq(GSE63282)/Homer(0.860) More Information | Similar Motifs Found	motif file (matrix)
3	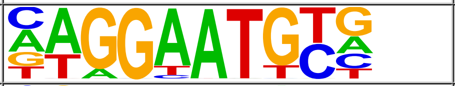	1e-618	-1.425e+03	12.68%	5.33%	53.2bp (59.8bp)	TEAD3/MA0808.1/Jaspar(0.964) More Information | Similar Motifs Found	motif file (matrix)
4	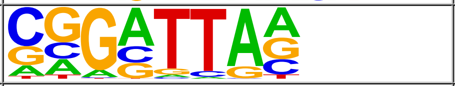	1e-457	-1.054e+03	31.30%	20.98%	55.8bp (61.0bp)	OTX1/MA0711.1/Jaspar(0.959) More Information | Similar Motifs Found	motif file (matrix)
5	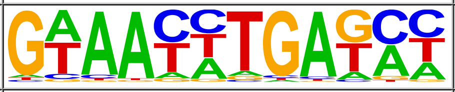	1e-262	-6.044e+02	6.41%	2.88%	53.9bp (58.5bp)	Six1(Homeobox)/Myoblast-Six1-ChIP-Chip(GSE20150)/Homer(0.975) More Information | Similar Motifs Found	motif file (matrix)
6	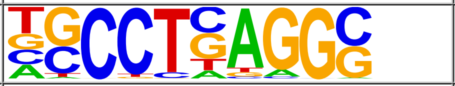	1e-252	-5.808e+02	7.94%	3.99%	53.5bp (60.7bp)	TFAP2A/MA0003.4/Jaspar(0.948) More Information | Similar Motifs Found	motif file (matrix)
7	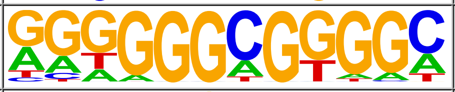	1e-239	-5.520e+02	11.50%	6.73%	53.5bp (60.9bp)	KLF14(Zf)/HEK293-KLF14.GFP-ChIP-Seq(GSE58341)/Homer(0.955) More Information | Similar Motifs Found	motif file (matrix)
8	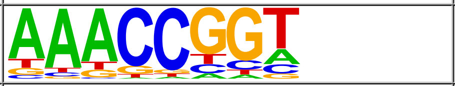	1e-233	-5.387e+02	28.96%	21.62%	56.4bp (60.8bp)	TFCP2/MA0145.3/Jaspar(0.908) More Information | Similar Motifs Found	motif file (matrix)
9	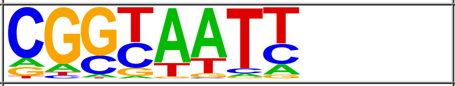	1e-196	-4.526e+02	23.52%	17.31%	55.6bp (62.2bp)	PH0024.1_Dlx5/Jaspar(0.779) More Information | Similar Motifs Found	motif file (matrix)
10	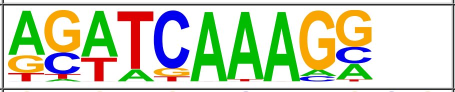	1e-166	-3.844e+02	5.85%	3.05%	55.1bp (60.2bp)	LEF1(HMG)/H1-LEF1-ChIP-Seq(GSE64758)/Homer(0.988) More Information | Similar Motifs Found	motif file (matrix)
11	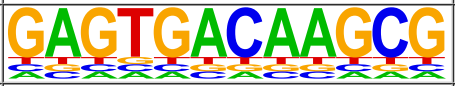	1e-132	-3.054e+02	2.19%	0.80%	53.0bp (66.2bp)	PBX3/MA1114.1/Jaspar(0.771) More Information | Similar Motifs Found	motif file (matrix)
12	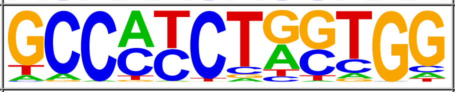	1e-125	-2.901e+02	2.07%	0.75%	50.7bp (59.8bp)	BORIS(Zf)/K562-CTCFL-ChIP-Seq(GSE32465)/Homer(0.923) More Information | Similar Motifs Found	motif file (matrix)
13	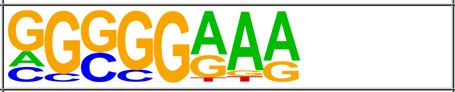	1e-94	-2.168e+02	28.68%	23.95%	56.9bp (63.1bp)	RO3G_00049(RRM)/Rhizopus_oryzae-RNCMPT00205-PBM/HughesRNA(0.817) More Information | Similar Motifs Found	motif file (matrix)
14	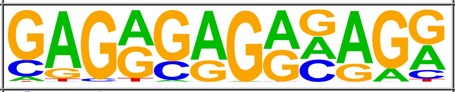	1e-80	-1.842e+02	6.44%	4.28%	54.7bp (55.3bp)	FRS9(ND)/col-FRS9-DAP-Seq(GSE60143)/Homer(0.848) More Information | Similar Motifs Found	motif file (matrix)
15	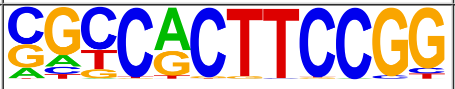	1e-74	-1.710e+02	1.20%	0.43%	53.4bp (56.7bp)	ZBTB7A/MA0750.2/Jaspar(0.945) More Information | Similar Motifs Found	motif file (matrix)
16	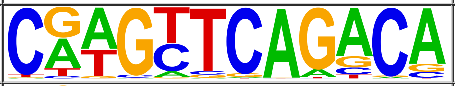	1e-65	-1.512e+02	0.17%	0.01%	55.4bp (42.3bp)	PB0060.1_Smad3_1/Jaspar(0.707) More Information | Similar Motifs Found	motif file (matrix)
17	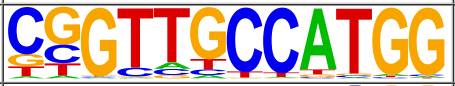	1e-54	-1.255e+02	0.73%	0.23%	52.8bp (55.6bp)	RFX3/MA0798.2/Jaspar(0.929) More Information | Similar Motifs Found	motif file (matrix)
18	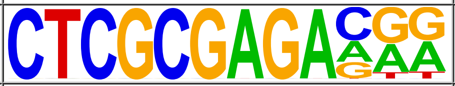	1e-51	-1.180e+02	0.14%	0.01%	55.7bp (0.0bp)	GFX(?)/Promoter/Homer(0.907) More Information | Similar Motifs Found	motif file (matrix)
							FEA4(bZIP)/Corn-FEA4-ChIP-	

19	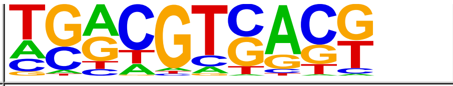	1e-45	-1.036e+02	0.71%	0.25%	52.5bp (65.1bp)	Seq(GSE61954)/Homer(0.872) More Information | Similar Motifs Found	motif file (matrix)
20	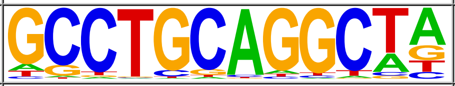	1e-39	-9.116e+01	1.45%	0.77%	53.8bp (62.9bp)	SOK2/MA0385.1/Jaspar(0.768) More Information | Similar Motifs Found	motif file (matrix)
21	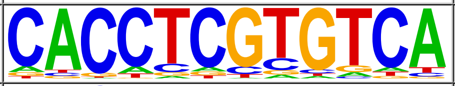	1e-38	-8.976e+01	0.21%	0.03%	51.8bp (28.5bp)	HLHm5/dmmpmm(Pollard)/fly(0.631) More Information | Similar Motifs Found	motif file (matrix)
22	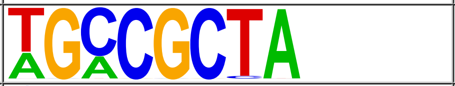	1e-34	-7.916e+01	3.45%	2.39%	57.1bp (67.1bp)	STP3/MA0396.1/Jaspar(0.780) More Information | Similar Motifs Found	motif file (matrix)
23	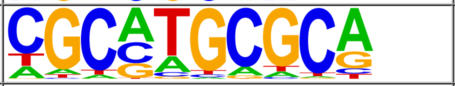	1e-33	-7.647e+01	1.05%	0.53%	53.6bp (69.3bp)	NRF1/MA0506.1/Jaspar(0.932) More Information | Similar Motifs Found	motif file (matrix)
24	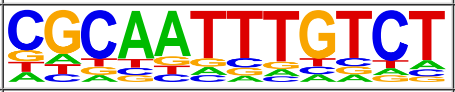	1e-32	-7.376e+01	0.12%	0.01%	47.9bp (49.5bp)	SGR5(C2H2)/colamp-SGR5-DAP-Seq(GSE60143)/Homer(0.730) More Information | Similar Motifs Found	motif file (matrix)
25	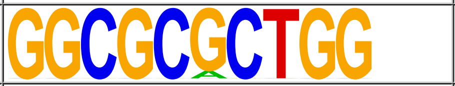	1e-23	-5.348e+01	0.11%	0.01%	58.4bp (57.7bp)	POL006.1_BREu/Jaspar(0.797) More Information | Similar Motifs Found	motif file (matrix)
26	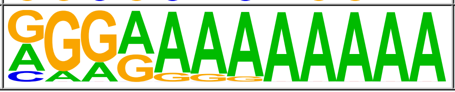	1e-15	-3.503e+01	2.15%	1.60%	57.0bp (57.0bp)	REM19(REM)/colamp-REM19-DAP-Seq(GSE60143)/Homer(0.859) More Information | Similar Motifs Found	motif file (matrix)
